# ENO2 sustains cancer stemness and metastatic competence through a phosphoenolpyruvate-dependent metabolic axis in triple-negative breast cancer

**DOI:** 10.1038/s41419-026-09106-0

**Published:** 2026-07-11

**Authors:** Cing-Hong Wang, Ru-Tsun Mai, Ke Jia Khoo, Chi-Feng Hung, Cheng-Kuang Yang, Meng-Ju Wu, Chi-Hong Chao

**Affiliations:** 1 Institute of Molecular Medicine and Bioengineering, National Yang Ming Chiao Tung University, Hsinchu, Taiwan, ROC; 2 Center for Intelligent Drug Systems and Smart Bio-devices (IDS2B), National Yang Ming Chiao Tung University, Hsinchu, Taiwan, ROC; 3 Department of Biological Science and Technology, National Yang Ming Chiao Tung University, Hsinchu, Taiwan, ROC; 4 Division of Urology, Department of Surgery, Ditmanson Medical Foundation Chia-Yi Christian Hospital, Chiayi, Taiwan, ROC; 5 Division of Onco-Urology, Department of Urology & Surgery, Taichung Veterans General Hospital, Taichung, Taiwan, ROC; 6 Division of Gastroenterology, Department of Medicine, University of Massachusetts Chan Medical School, Worcester, MA USA

**Keywords:** Cancer metabolism, Cancer stem cells, Cancer stem cells, Mechanisms of disease, Breast cancer

## Abstract

Enolase 2 (ENO2) is a neuron-specific glycolytic enzyme whose expression is elevated in aggressive breast cancers, yet its enzymatic and biological contributions to triple-negative breast cancer (TNBC) progression remain incompletely defined. Here, we demonstrate that ENO2 sustains cancer stem cell (CSC) properties and metastatic competence through a phosphoenolpyruvate (PEP)-dependent metabolic axis. Elevated ENO2 expression correlated with advanced tumor grade and poor clinical outcomes in TNBC cohorts, underscoring its clinical relevance. Genetic depletion of ENO2 impaired aerobic glycolysis and oxidative phosphorylation, reduced migration, invasion, and CSC frequency, and suppressed tumor growth and pulmonary metastasis in orthotopic models. Mechanistically, exogenous PEP or pyruvate restored CSC-associated traits and invasiveness in ENO2-deficient cells, supporting the functional involvement of ENO2-derived metabolites in CSC maintenance. Reconstitution with wild-type ENO2, but not a catalytically impaired mutant, restored CSC properties, invasiveness, and metastatic colonization, establishing that ENO2 catalytic activity is required for these malignant traits. Further analysis revealed that PKM2 perturbation preferentially attenuated PEP-mediated rescue while largely sparing pyruvate-mediated rescue, supporting a functional PEP-PKM2-pyruvate axis in CSC regulation. Consistently, PKM2 depletion partially blunted ENO2-mediated rescue; however, residual rescue despite PKM2 perturbation suggested additional PKM2-independent PEP-responsive mechanisms. Importantly, pharmacological inhibition of enolase with POMHEX phenocopied genetic ENO2 loss and suppressed CSC maintenance in vitro and tumor growth in vivo. Taken together, these findings identify the ENO2-driven PEP-dependent metabolic axis as a mechanistic link between metabolic reprogramming, cancer stemness, and metastasis, revealing a therapeutically actionable metabolic vulnerability in TNBC.

## Introduction

Enolase, the dimeric protein that converts 2-phosphoglycerate (2-PGA) to phosphoenolpyruvate (PEP), is a critical glycolytic enzyme involved in a wide spectrum of biological functions and plays a pivotal role in the tumorigenesis of multiple types of cancer [1]. Three *ENO* genes have been identified in the human genome, *ENO1*, *ENO2*, and *ENO3*, which encode the α-, β-, and γ-subunits of enolase, respectively. The dimerization between these three subunits results in five different isoforms of enolase: αα, ββ, γγ, αβ, and αγ [1, 2]. Among these isoforms, ENO1, also named α-enolase, ENO1 exhibits ubiquitous expression across a wide range of vertebrate tissues, spanning from embryonic stages to adulthood [3], and its elevated expression has been observed in a wide array of cancer types, including lung, liver, breast, etc [4]. Aberrant ENO1 expression facilitates cancer progression via both its enzymatic activity and non-canonical “moonlighting” functions, thereby highlighting ENO1 as a potential therapeutic target [4].

Unlike ENO1, which is ubiquitously expressed, ENO2 (γ-enolase) exhibits neuron-specific expression under physiological conditions. Although the aberrant ENO2 expression is observed in a narrow spectrum of cancers compared to ENO1, ENO2 has been shown to play critical roles in the progression of lung, pancreatic, colon, kidney and head-and-neck cancers via both its glycolytic activity and moonlighting functions [5–9] and serves as a therapeutic target in gliomas with ENO1 deficiency [10].

Emergent evidence also links ENO2 to breast cancer pathogenesis. While ENO1 is the predominant enolase isoform in normal human breast tissue, ENO2 expression is typically low or undetectable but becomes markedly upregulated in breast malignancies [11]. Furthermore, both acute and chronic As^+3^ and Cd^+2^ exposure have been observed to stimulate ENO2 expression. This suggests a possible involvement of ENO2 in As^3+^ and Cd^2+^-mediated transformation of normal mammary epithelial cells [12]. Elevated ENO2 expression has also been noted in 3D-cultured lung and breast cancer cells, suggesting a role of ENO2-enhanced glycolysis in supporting tumor spheroid growth under three-dimensional conditions [13]. Moreover, the upregulated expression and increased copy number of *ENO2*, attributed to the amplification or gain of chromosome regions 12p 12.1-13.3, are regarded as distinguishing features of the metabolic-pathway-based subtype 2 (MPS2)-triple-negative breast cancer (TNBC). This subtype accounts for 36.9% of the 360 TNBC cases and represents the most glycolytic and clinically aggressive subset among the MPS1-3 classifications, suggesting that ENO2 may contribute to metabolic reprogramming in aggressive TNBC [14]. Collectively, these observations support a plausible role for ENO2 in shaping TNBC metabolism and facilitating metastatic progression by enabling tumor cells to overcome metabolic stress during the metastasis cascade. However, the precise biological functions and mechanistic contributions of ENO2-mediated metabolism in TNBC progression remain incompletely understood.

TNBC is a clinical subtype pathologically defined by the absence of estrogen receptor (ER), progesterone receptor (PR), and human epidermal growth factor receptor 2 (HER2) expressions, accounting for ~15–20% of all breast cancers and disproportionately affecting younger patients. TNBC largely overlaps with the basal-like breast cancer (BLBC) molecular subtype classified by gene expression profiling, and both are characterized by high histological grade, genomic instability, poor prognosis, and a lack of effective targeted therapies. Current treatment strategies rely primarily on chemotherapy, yet high rates of relapse and therapeutic resistance underscore an urgent need for new treatment approaches [15–18].

In this study, we demonstrated that ENO2 expression strongly correlates with advanced tumor grade and poor clinical outcomes in TNBC patients. Mechanistically, ENO2 promotes tumor growth, pulmonary metastasis, and the maintenance of cancer stem cell (CSC) traits through its metabolic activity. Importantly, pharmacological inhibition of ENO2 using the selective enolase inhibitor POMHEX significantly impairs cell proliferation, CSC self-renewal, and in vivo tumor growth, establishing ENO2 as both a prognostic biomarker and a promising therapeutic target in TNBC.

## Materials and methods

### Cell lines and culture

Human normal mammary epithelial cell lines, MCF10A (ATCC Cat. No. CRL-10317, RRID: CVCL_0598) and MCF12A (ATCC Cat. No. CRL-10782, RRID: CVCL_3744), human luminal breast cancer cell lines, MCF-7 (ATCC Cat. No. HTB-22, RRID: CVCL_0031) and MDA-MB-436 (ATCC Cat. No. HTB-130, RRID: CVCL_0623), human basal-like breast cancer cell lines, BT-20 (ATCC Cat. No. HTB-19, RRID: CVCL_0178), BT549 (ATCC Cat. No. HTB-122, RRID: CVCL_1092), MDA-MB-231 (ATCC Cat. No. HTB-26, RRID: CVCL_0062), and HS578T (ATCC Cat. No. HTB-126, RRID: CVCL_0332); as well as murine breast cancer cell line 4T1 (ATCC Cat. No. CRL-2539, RRID: CVCL_0125), were purchased from American Type Culture Collection (ATCC) during 2016-2017. ATCC has declared that all of the cell lines are mycoplasma-free. Cryopreservation of cell lines was performed at passages 3 (grown for 7–8 days) after receipt. Cells have been cultured for more than 10–15 passages (30 passages for CRISPR-mediated KO clones) and discarded to ensure cell authentication and avoid mycoplasma contamination. For animal studies, all cell lines were additionally tested for mycoplasma contamination using the Mycoplasma Detection Kit (BSMP101, Bio Smart Co., LTD, Taiwan) and confirmed negative. All of the cell lines were cultured according to the guidelines provided by ATCC.

### Chemicals

POMHEX (Cat. No. HY-131904, MedChemExpress) and phosphoenolpyruvate (PEP; CAS No. 5541-93-5, Sigma-Aldrich) were purchased from the indicated suppliers.

### Plasmid construction

Lentiviral CRISPR/Cas9 vectors (pAll-Cas9.Ppuro-control and pAll-Cas9.Ppuro-ENO2-sg3) and ENO2 shRNA constructs (shENO2#1, shENO2#2, and shENO2#3) were obtained from the National RNAi Core Facility (Academia Sinica, Taiwan). Oligonucleotide sequences and plasmid maps are available upon request.

To generate the wild-type ENO2 expression vector (pCDH-FLAG-ENO2), FLAG-tagged ENO2 cDNA was amplified by PCR and cloned into the pCDH-CMV-MCS-EF1-Puro lentiviral backbone (System Biosciences). The ENO2-K394R mutant was generated by site-directed mutagenesis using primers K394R-F (GTGCACAGGCCAGATCCGGACTGGTGCCCCGTGCC) and K394R-R (GGCACGGGGCACCAGTCCGGATCTGGCCTGTGCAC).

### Generation of stably expressed, knocked-down (knockdown, KD) or knocked-out (knockout, KO) cell lines

BT-549 and MDA-MB-231 cells were transduced with lentiviral vectors for ENO2 overexpression, knockdown, or knockout as previously described [19]. Puromycin (2 micrograms per milliliter) or blasticidin (10 micrograms per milliliter) selection for two weeks yielded stable pools.

For bioluminescence imaging, MDA-MB-231 cells were infected with pLenti6/V5-Luc2 [20] and selected with blasticidin for two weeks to obtain MDA-MB-231-Luc2. CRISPR knockout clones (MDA-MB-231-ENO2-KO; MDA-MB-231-Luc2-ENO2-KO) were generated using pAll-Cas9.Ppuro-ENO2-sg3, followed by six weeks of puromycin selection. Single clones were isolated, expanded, and validated by immunoblotting.

### Cell proliferation and colony formation assays

Cell proliferation and colony formation assays were performed as previously described [21] with minor modifications. Cell proliferation was determined by MTS assays or direct cell counting。 For direct cell counting, MDA-MB-231-derived cells were seeded in 6-well plates at a density of 1 × 10⁵ cells per well. Three days after seeding, viable cells were harvested and counted by flow cytometer. For colony formation assays, cells (5 × 10³) were plated in 6-cm dishes and cultured for 14 days. Colonies were fixed with methanol, stained with crystal violet, and counted to determine clonogenic efficiency.

### Cell survival assay and IC₅₀ determination

Cell viability was assessed by MTS assay (Promega). BT-549 (8 × 10³ cells per well) and MDA-MB-231 (6 × 10³ cells per well) cells were seeded into 96-well plates, treated with the indicated drug concentrations for two days, and measured at 490 nm using a CLARIOstar reader (BMG LABTECH). IC50 values were calculated using GraphPad Prism version 6.

### Mammosphere formation and transwell migration/invasion assays

Mammosphere assays were performed as described [19]. Cells were grown in non-adherent conditions for 6 to 7 days. Sphere-forming efficiency was calculated as the sphere number divided by the seeded cell number. For secondary sphere assays, dissociated primary spheres (5 × 10⁴ cells per well) were reseeded for 6 to 7 days.

For migration assays, 5 × 10⁴ cells were seeded into 8-µm transwell inserts (Corning). For invasion assays, inserts were precoated with 100 microliters of 2 percent Matrigel. Migrated or invaded cells were fixed and crystal violet-stained after 24 h for BT-549 and MDA-MB-231 migration assays and after 40 h for MDA-MB-231 invasion assays.

### Total RNA extraction and real-time-PCR (RT-PCR)

Total RNA was extracted using the RNeasy Mini Kit (Qiagen) and reverse-transcribed using the Transcriptor First Strand cDNA Synthesis Kit (Roche). qRT-PCR was performed using KAPA SYBR FAST (Merck) as previously described [19]. Fluorescence was acquired using a CFX Connect system (Bio-Rad). All reactions were performed in triplicate. Primer sequences are listed below: ACTB-F (CACCATTGGCAATGAGCGGTTC); ACTB-R (AGGTCTTTGCGGATGTCCACGT); ENO2-F (CGTTACTTAGGCAAAGGTGTCC), ENO2-R (CTCCAGCATCAGGTTGTCCAGT); SOX4-F (GACATGCACAACGCCGAGATCT); SOX4-R (GTAGTCAGCCATGTGCTTGAGG); SOX2-F (GCTACAGCATGATGCAGGACCA); SOX2-R (TCTGCGAGCTGGTCATGGAGTT); OCT3/4-F (CCTGAAGCAGAAGAGGATCACC); OCT3/4-R (AAAGCGGCAGATGGTCGTTTGG). E-Cadherin-F (GCCTCCTGAAAAGAGAGTGGAAG); E-Cadherin-R (TGGCAGTGTCTCTCCAAATCCG); N-Cadherin-F (CCTCCAGAGTTTACTGCCATGAC); N-Cadherin-R (GTAGGATCTCCGCCACTGATTC); β-catenin-F (CACAAGCAGAGTGCTGAAGGTG); β-catenin-R (GATTCCTGAGAGTCCAAAGACAG); VEGF-F (CCTGCAAGATTCAGGCACCTATG); VEGF-R (GTTTCGCAGGAGGTATGGTGCT).

### Antibodies for immunoblotting

The following antibodies were used: anti-ENO2 (1:2000, Santa Cruz, sc-21738), anti-N-cadherin (1:250, Santa Cruz, sc-59987), anti-vimentin (1:500, Santa Cruz, sc-6260), and anti-Actin (1:10,000, Sigma, A5441), anti-Caspase-3 p17/p19 antibody (1:1000, iReal, IR96-401), anti-PARP1 antibody (1:1000, iReal, IR101-421), anti-Cyclin D1 antibody (1:1000, iReal, IR117-294), anti-beta-catenin/CTNNB1 antibody (1:2000, iReal, IR49-149), and anti-PKM2 antibody (1:2000, cell signaling, #3198). Uncropped original Western blot images were shown in the supplementary materials.

### Immunohistochemistry (IHC) and staining evaluation

IHC was performed on commercial TNBC tissue microarrays (BR1301a and BRE1201; TissueArray.com). Staining was conducted on a Ventana DISCOVERY ULTRA system (Roche Tissue Diagnostics) using OptiView DAB Detection (Cat. No. 760-700, Roche Diagnostics) and OptiView Amplification kits (Cat. No. 760-099, Roche Diagnostics).

Heat-mediated antigen retrieval was performed for 32 min at 100 °C using ULTRA cell conditioning solution (CC1, pH 8.5). ENO2 was detected using anti-NSE/Enolase-2 (IR31-90, 1:200; iReal Biotechnology). ENO2 immunohistochemical staining was quantified using an optical-density-based digital image analysis approach in ImageJ/Fiji. Digital images of immunostained tumor sections were subjected to color deconvolution to isolate the DAB chromogenic signal. For each case, the DAB signal within the tumor area was quantified, and the mean optical density (OD) value was calculated as an objective measure of ENO2 staining intensity.

To classify ENO2 staining intensity, all cases were ranked in ascending order according to their mean OD values and divided into three approximately equal tertiles. Cases in the lowest tertile were classified as the negative/weak-staining group, cases in the middle tertile as the moderate-staining group, and cases in the highest tertile as the strong-staining group. After classification, the grade and stage of each case within the three OD-based staining groups were recorded. Associations between ENO2 staining intensity and tumor grade were analyzed using Fisher’s exact test.

### FACS analysis of breast cancer stem cell-enriched populations

CSC-enriched subpopulations were analyzed by flow cytometry (NovoCyte, ACEA). CD44⁺/CD24⁻ populations were detected using FITC-conjugated anti-CD44 (1:100; BD Biosciences, AB_395870) and PE-conjugated anti-CD24 (1:100; BD Biosciences, AB_395822). ALDH activity was measured using the ALDEFLUOR kit (STEMCELL Technologies, 01700) according to the manufacturer’s instructions. ALDH⁺ CSC populations were identified by flow cytometry based on ALDEFLUOR fluorescence, with the DEAB-treated sample used as a negative control to define the ALDH⁺ gate.

### Measurement of oxygen consumption rate (OCR) and extracellular acidification rate (ECAR)

OCR and ECAR were measured using the Seahorse XFp Analyzer (Agilent) as described [19]. Cells were seeded at 5 × 10⁴ (MDA-MB-231) or 6 × 10⁴ (BT-549) per well and incubated for 16 to 20 h. OCR was measured in XF Assay Medium supplemented with 10 mM glucose and GlutaMAX. Oligomycin, FCCP, and rotenone/antimycin A were sequentially injected. ECAR was measured in glucose-free medium after sequential addition of glucose, oligomycin, and 2-deoxyglucose. Data were analyzed using Wave software (Agilent).

### In vitro extreme limiting dilution assay (ELDA)

BT-549 cells were plated at 50, 100, 500, and 2000 cells per well; MDA-MB-231 cells were plated at 100, 500, 2000, and 5000 cells per well (24 wells per dilution). After seven days, sphere-containing wells were quantified, and CSC frequency was calculated using the ELDA webtool (https://bioinf.wehi.edu.au/software/elda/) [22].

### In vivo studies

For the orthotopic xenograft TNBC model, female NOD-SCID mice (NOD.CB17-Prkdcscid/JNarl; 8–10 weeks old; purchased from the National Center for Biomodels, Taipei, Taiwan) were inoculated with TNBC cells (3 × 10⁶ MDA-MB-231-Luc2 or BT-549 cells) mixed with Matrigel (Matrigel: PBS = 1:1) into the fourth mammary fat pad. Tumor size was measured every three days with a caliper, and tumor volume was calculated using the formula: (d1 × d2²)/2, where d1 is the larger diameter and d2 is the smaller diameter.

For the in vivo extreme limiting dilution assay, BT-549-shENO2#1, BT-549-shENO2#3, and control cells were injected orthotopically into the mammary fat pads at four doses (5 × 10⁴, 5 × 10⁵, 1 × 10⁶, and 3 × 10⁶ cells). Tumor incidence was determined 44 days post-inoculation, and stem cell frequency was calculated using ELDA software.

For the experimental metastasis model, 1 × 10⁶ MDA-MB-231-Luc2 control, shENO2, or ENO2-KO cells were suspended in 100 μL PBS and injected into the tail vein of NOD-SCID mice. Pulmonary metastases in both orthotopic and tail-vein injection models were monitored by bioluminescence imaging using the in vivo imaging system (IVIS), as described previously [20].

To evaluate therapeutic efficacy, POMHEX (Cat. No. HY-131904) was dissolved in PBS containing 0.1% DMSO and administered by intraperitoneal injection (30 mg/kg). Treatment was initiated either at the time of tumor inoculation or when tumor volumes reached 0.1 cm³.

All animal experiments and husbandry procedures were performed in accordance with protocols approved by the Institutional Animal Care and Use Committee (IACUC) of National Yang Ming Chiao Tung University, Taiwan (IACUC No. 112051 A and No. 111045). According to the approved animal care and use protocol, the humane endpoint for tumor-bearing animals was defined as a maximum tumor diameter of 2 cm. Throughout the study, all tumors remained below this predefined limit. For animal studies, sample size was estimated using an expected standard deviation of 20%, a statistical power of 80%, and *P* < 0.05 as the threshold for statistical significance. All mice were included in the analysis, and no animals were excluded. Mice were randomly assigned to experimental groups before treatment or tumor implantation, as appropriate. The studies were not conducted or analyzed in a blinded manner.

### Statistical analysis

All experiments were performed with at least three independent biological replicates unless otherwise indicated. Data from independent experiments are presented as mean ± SD unless otherwise stated. For representative experiments, data are presented as mean ± SD from three technical replicates (*n* = 3), with consistent results observed across independent biological replicates.

For pairwise comparisons between two groups, equality of variance was first assessed using an F-test. If variances were not significantly different (*p* > 0.05), statistical significance was determined using an unpaired two-tailed Student’s *t*-test. If variances were significantly different (*p* < 0.05), Welch’s correction was applied. For non-parametric comparisons, the Mann-Whitney U test was used where appropriate. For comparisons among multiple groups, ANOVA was used as indicated. Statistical tests used for individual experiments are specified in the corresponding figure legends.

For analyses integrating multiple independent experiments, statistical evaluation was performed using two-way ANOVA, with biological replicates, defined as independent experiments, used as the unit of analysis. Data from independent experiments are presented as mean ± SD. Interaction effects were assessed between metabolite treatment, PEP or pyruvate, and PKM2 perturbation, compound 3K-mediated PKM2 inhibition or shPKM2-mediated PKM2 knockdown, as well as between ENO2 reconstitution and PKM2 depletion, where indicated.

For analyses in Fig. 6F, G, J, K, N, sphere-forming efficiencies were normalized to the corresponding untreated or control condition within each independent experiment to generate relative values. These normalized values were then used for two-way ANOVA to assess interaction effects across independent experiments.

For interaction plot analyses in Fig. 6H, L, O, data from independent experiments were used. In Fig. 6H, L, metabolite-induced rescue effects were quantified as fold changes, calculated as treated/untreated values within each PKM2 condition. These values were analyzed by two-way ANOVA to evaluate metabolite × PKM2 interaction effects. In Fig. 6O, relative sphere-forming efficiencies were analyzed by two-way ANOVA to evaluate the interaction between ENO2 reconstitution and PKM2 depletion.

A *p* value < 0.05 was considered statistically significant. Statistical significance is indicated in the figures as follows: *p < 0.05, **p < 0.01, ***p < 0.001; ns, not significant.

The studies were not conducted or analyzed in a blinded manner.

## Results

### Higher ENO2 expression predicts adverse prognosis and associates with advanced tumor grade in TNBC

Analysis of 122 paired tissues from TNMplot [23] revealed significantly elevated ENO2 expression in breast tumors compared with normal tissues (Supplementary Fig. S1A), consistent with higher ENO2 protein levels in multiple breast cancer cell lines (Supplementary Fig. S1B). Analysis of PAM50-stratified datasets from bc-GenExMiner [24] and GENT2 [25] further demonstrated subtype-specific differences in ENO2 expression, with luminal B tumors showing the highest expression among these molecular subtypes (Supplementary Fig. S1C).

**Fig. 1:**
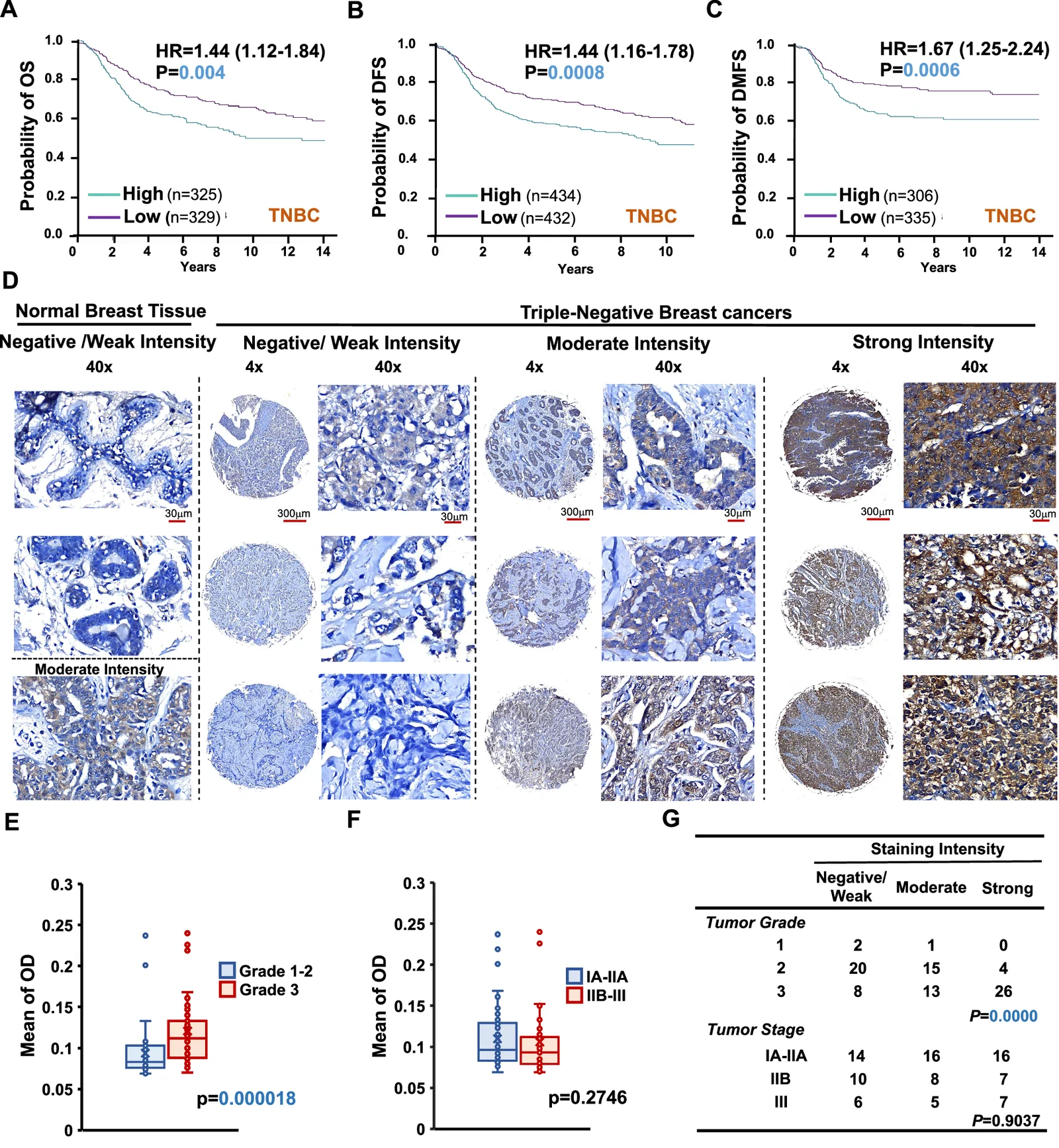
High ENO2 expression predicts an unfavorable prognosis in TNBC patients. Kaplan–Meier survival analyses of triple-negative breast cancer (TNBC) patients stratified by ENO2 expression. Overall survival (OS; **A** *n* = 654), disease-free survival (DFS; **B** *n* = 866), and distant metastasis–free survival (DMFS; **C** *n* = 641) were analyzed using bc-GenExMiner v4.8 across total microarray datasets. Patients were classified into ENO2-high (blue; *n* = 325 for OS, *n* = 434 for DFS, *n* = 306 for DMFS) and ENO2-low cohorts (purple; *n* = 329 for OS, *n* = 432 for DFS, *n* = 335 for DMFS) based on median expression. Hazard ratios (HRs) with 95% confidence intervals (CIs) are indicated, and statistical significance was determined using the log-rank test. **D** Representative immunohistochemistry (IHC) images (4× and 40× magnification) from a TNBC tissue array (BRE1201) illustrating negative/weak, moderate, and strong ENO2 staining intensities. Box-and-whisker plots showing the distribution of mean optical density (OD) values of IHC staining across tumor grades (**E** grades 1–2, *n* = 43; grade 3, *n* = 77) and stages (**F** stages IA–IIA, *n* = 64; stages IIB–III, *n* = 56). Each dot represents the average OD for a single tumor section. Data are presented as mean ± SD, and statistical comparisons were made using two-tailed Mann-Whitney U tests. **G** Statistical association of ENO2 expression with tumor grade and stage was examined by Fisher’s exact test and the chi-square test, respectively.

Despite lower expression in BLBC compared with other subtypes, ENO2 showed a stronger prognostic association in this group. High ENO2 expression correlated with worse overall survival (OS) and distant metastasis-free survival (DMFS) in BLBC across both KM Plotter and bc-GenExMiner databases (Supplementary Figs. S2–4). Given the overlap between BLBC and TNBC [26], we further examined ENO2 prognostic significance specifically in TNBC. Elevated ENO2 expression significantly predicted inferior OS, disease-free survival (DFS), and DMFS in TNBC cohorts (Fig. 1A–C and Supplementary Fig. S5).

IHC analyses of two independent TNBC tissue arrays (Fig. 1D, *n* = 89, Supplementary Table 1 and Supplementary Fig. S6A, *n* = 120; Supplementary Table 2) revealed consistently higher ENO2 expression in high-grade (grade 3) compared with low-grade (grades 1-2) tumors. In contrast, ENO2 expression did not differ significantly between early-stage (stage IA-IIA) and late-stage (stage IIB-III) tumors (Fig. 1E–G and Supplementary Fig. S6B, E). Together, these results demonstrate that ENO2 is aberrantly upregulated in breast cancer and confers its strongest prognostic disadvantage in BLBC and TNBC, where it is closely associated with high tumor grade and poor clinical outcomes.

### ENO2 is required for TNBC tumor growth and metastasis in vivo

**Fig. 2:**
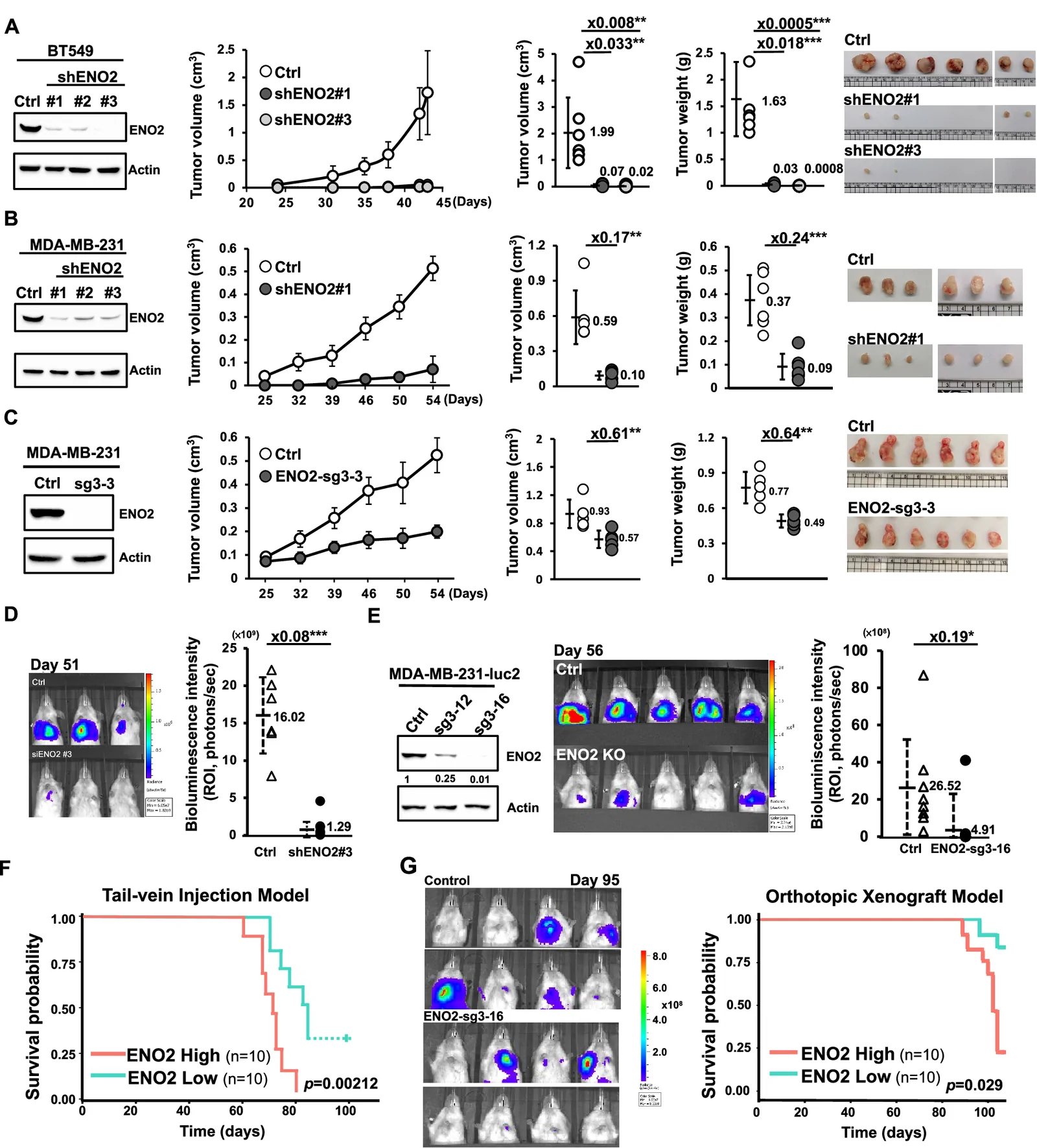
ENO2 depletion suppresses tumor growth, pulmonary metastasis, and improves survival in TNBC xenograft models. Orthotopic xenograft assays using ENO2-deficient TNBC cells. BT-549-ENO2-KD (**A** *n* = 7), MDA-MB-231-ENO2-KD (**B** *n* = 6), and MDA-MB-231-ENO2-KO (**C** *n* = 6) cells (3 × 10⁶) were inoculated into the fourth mammary fat pad of female NOD-SCID mice and monitored for 8 weeks. Left: decreased ENO2 expression was confirmed by Western blotting. Right: Tumor sizes were measured every 3 days and plotted as growth curves. At the endpoint, tumors were excised, and tumor volumes were calculated using the formula: (d₁ × d₂²)/2 (d₁, larger diameter; d₂, smaller diameter). Tumor volumes and weights are presented as dot plots (mean ± SD), with fold-change values and statistical significance indicated above comparison lines. Data were analyzed using an unpaired Student’s *t*test (**p < 0.01; ***p < 0.001). Pulmonary metastasis assays following lateral tail-vein injection of 1 × 10⁶ MDA-MB-231-Luc2 ENO2-deficient cells (**D** shENO2#3, *n* = 6; **E** ENO2-sg3-16, *n* = 10) or controls (**D** *n* = 6; **E** *n* = 10). Metastatic burden was quantified by in vivo bioluminescence imaging (IVIS). Left: representative images; Right: chest bioluminescence intensities (photons/sec) plotted as mean ± SD. Unpaired *t*-test: *p < 0.05, ***p < 0.001. Kaplan–Meier survival curves of NOD-SCID mice inoculated with MDA-MB-231-Luc2-ENO2-KO (ENO2-sg3-16, *n* = 10) or control cells (*n* = 10) via tail-vein injection (**F**) or orthotopic xenografting (**G**). Survival was recorded from first to last death and plotted with IBM SPSS Statistics 24. Statistical significance was determined by the log-rank test (*p* < 0.05). Representative IVIS images of pulmonary metastases at day 95 post-inoculation are shown in (**G**, left).

To functionally validate the clinical association between ENO2 and aggressive TNBC behavior, ENO2 was depleted in MDA-MB-231 and BT-549 cells using either RNA interference (Fig. 2A, B) or CRISPR/Cas9-mediated knockout (Fig. 2C). Orthotopic implantation of ENO2-deficient cells into the mammary fat pads of immunodeficient mice revealed a marked suppression of tumor growth, as evidenced by significantly slower kinetics, reduced tumor volumes, and decreased endpoint weights compared with control tumors (Fig. 2A–C). These data demonstrate that ENO2 is required for efficient primary TNBC tumor outgrowth in vivo.

We next examined whether ENO2 influences metastatic progression. In experimental tail-vein metastasis models, both ENO2-knockdown and ENO2-knockout MDA-MB-231 cells formed significantly fewer pulmonary metastatic lesions, as quantified by IVIS bioluminescence imaging (Fig. 2D, E). Mice injected with ENO2-knockout cells also exhibited significantly prolonged survival (Fig. 2F). Consistent survival benefits were observed in orthotopic xenograft models, in which ENO2 knockout significantly extended overall survival (Fig. 2G), with a similar but less pronounced trend in ENO2-knockdown tumors (Supplementary Fig. S7).

Together, these in vivo studies demonstrate that ENO2 is functionally required for both primary tumor growth and metastatic dissemination in TNBC, supporting ENO2 as an important contributor to TNBC progression.

### ENO2 depletion impairs migration, invasion, and cancer stemness of TNBC cells

**Fig. 3:**
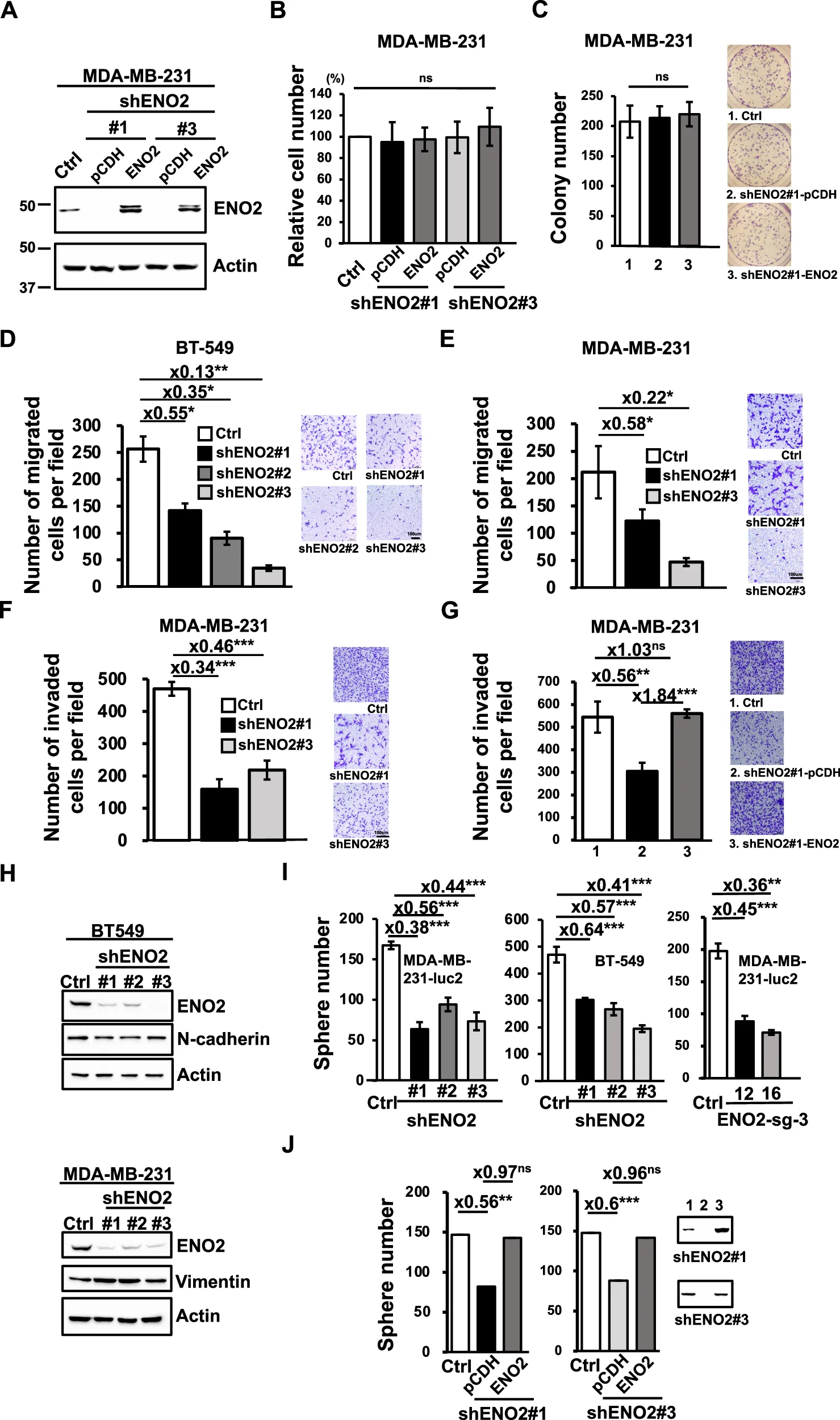
Diminishing ENO2 expression suppresses metastatic traits, including migration, invasion and cancer stemness, without affecting EMT. **A** ENO2 expression in MDA-MB-231-control (Ctrl), ENO2-KD clones (shENO2#1 and #3), and ENO2-KD clones expressing exogenous ENO2 were examined by western blotting. Growth curves of ENO2-deficient TNBC cells determined by MTS assay over a 3-day culture period (**B**) or colony formation assay over a 14-day culture period **(C).** Cell migration (**D, E**) and invasion (**F**) were evaluated by transwell assays using ENO2-knockdown and control BT-549 (**D**) or MDA-MB-231 (**E, F**) cells. **G** MDA-MB-231-ENO2-KD cells reconstituted with exogenous ENO2 were subjected to an invasion assay. Migrated/invaded cells on the lower membrane surface were fixed, crystal violet–stained, and quantified by averaging counts from 5 random microscopic fields per well. Representative micrographs are provided. **H** Expression of EMT markers examined by immunoblotting. The expression of ENO2, Vimentin, N-cadherin, and Actin (internal control) in ENO2-deficient TNBC cells and their controls was analyzed by Western blotting. Representative cropped blots are shown. **I** Mammosphere formation in control and ENO2-deficient TNBC cells. ENO2-deficient BT-549 and MDA-MB-231 cells were cultured in suspension for 7–8 days. Spheres >40 μm were quantified. **J** Rescue of mammosphere-forming efficiency by ENO2 reintroduction in MDA-MD-231-shENO2 cells. MDA-MD-231-shENO2 cells (shENO2#1 and #3) were transduced with ENO2-expressing or parental pCDH constructs and subjected to sphere formation assay. ENO2 protein expression was confirmed by immunoblotting. All quantitative data in this figure are shown from one representative experiment and presented as mean ± SD from three technical replicates (*n* = 3). Similar trends were observed across at least three independent experiments. Statistical significance was determined using an unpaired two-tailed Student’s t-test: ns, not significant; *p < 0.05; **p < 0.01; ***p < 0.001.

To elucidate the cellular mechanisms underlying the impaired tumor progression observed in vivo, we next assessed the impact of ENO2 loss on proliferation, motility, and cancer stemness (Fig. 3). Wild-type ENO2 was re-expressed in ENO2-knockdown TNBC cells to confirm the specificity of ENO2-dependent phenotypes (Fig. 3A). ENO2 depletion did not significantly affect proliferative capacity in MDA-MB-231 cells, as assessed by MTS assay, and clonogenic growth (colony formation), and re-expression of ENO2 had no detectable impact on these parameters (Fig. 3B, C), suggesting that ENO2 loss does not substantially trigger apoptotic cell death or overt cell-cycle perturbation under these conditions.

In contrast, ENO2 depletion had a pronounced impact on cell motility. Transwell assays revealed significant reductions in both migration (Fig. 3D, E) and invasion (Fig. 3F) following ENO2 knockdown, whereas re-expression of wild-type ENO2 fully restored invasive capacity (Fig. 3G). Although epithelial-mesenchymal transition (EMT) is a well-established driver of metastatic behavior, expression levels of canonical mesenchymal markers, including vimentin and N-cadherin, were unchanged following ENO2 depletion (Fig. 3H), indicating that ENO2-regulated migration and invasion are not accompanied by affecting EMT.

Given the strong in vivo effects of ENO2 on tumor growth and metastasis, we next asked whether ENO2 contributes to CSC properties. ENO2 silencing by either RNA interference or CRISPR-mediated knockout resulted in a 40-60% reduction in primary mammosphere-forming efficiency (Fig. 3I). This defect was fully rescued by restoring ENO2 expression in MDA-MB-231 cells (Fig. 3J) and similarly in BT-549 cells (Supplementary Fig. S8), indicating that ENO2 is required for maintaining CSC-associated phenotypes.

### ENO2 is essential for maintaining CSC-enriched populations and tumor-initiating capacity in TNBC

**Fig. 4:**
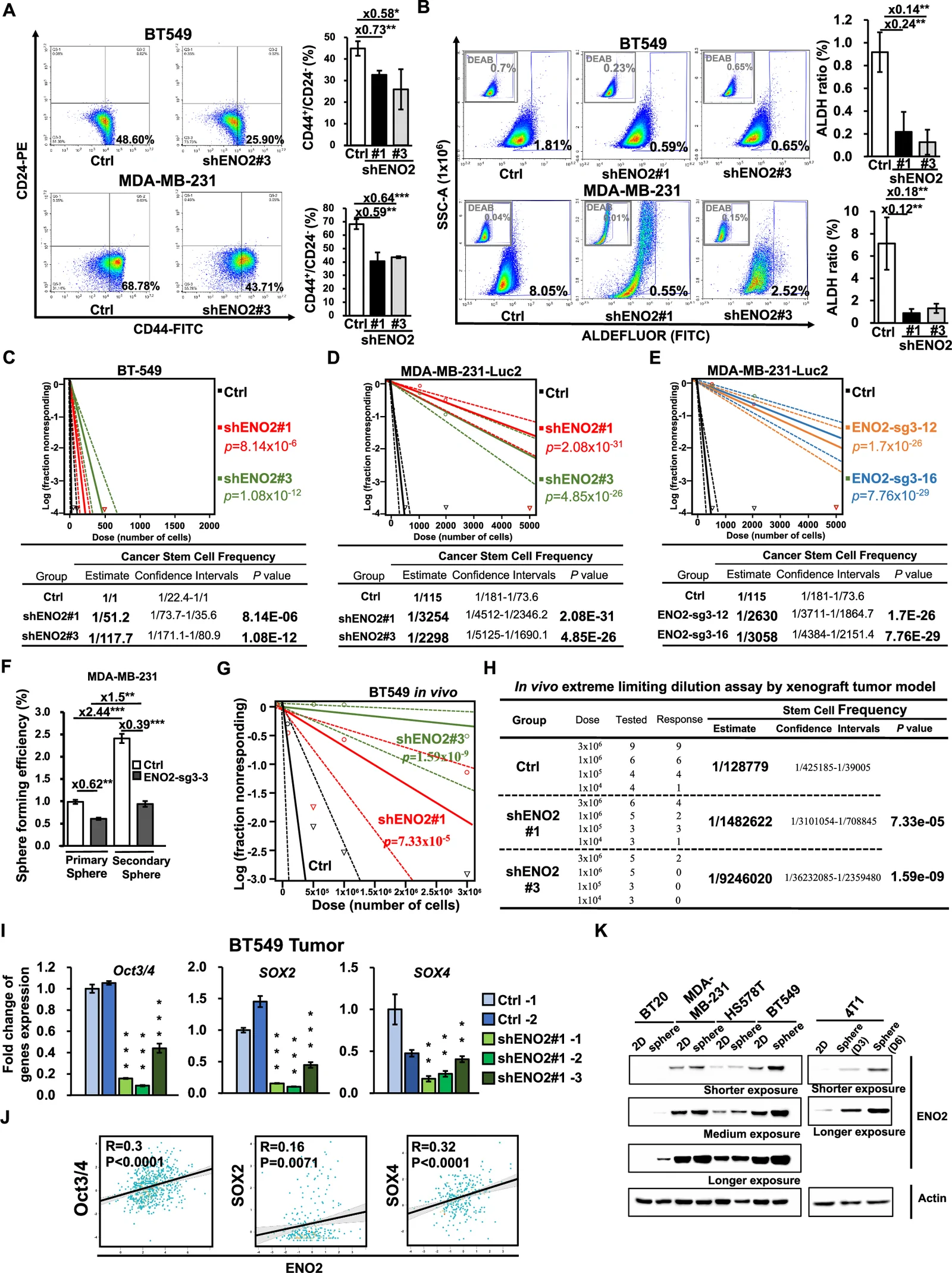
ENO2 depletion impairs CSC properties in TNBC. **A-B**. Flow cytometry analysis of CSC-enriched populations in mammospheres. CD44⁺/CD24⁻ cells were quantified after 7–8 days of suspension culture (**A**), and ALDH⁺ cells were measured by ALDEFLUOR assay with or without DEAB control (**B**). Quantification shown as mean ± SD from three independent biological replicates (n = 3 independent experiments). **C-E**. CSC frequencies of BT-549 (**C**), MDA-MB-231-Luc2 KD (**D**), and MDA-MB-231-Luc2 KO (**E**) cells were estimated by limiting dilution assays (ELDA). Data are plotted as log fraction of non-responding wells vs. plated cell number. Estimated CSC frequency and 95% confidence intervals are provided. Representative ELDA results from one of three independent experiments are shown, with similar results observed across experiments. **F** Secondary mammosphere formation of control and ENO2-KO MDA-MB-231 cells. Sphere-forming efficiency was calculated as the ratio of sphere number to seeded cell number. Data are shown from one representative experiment and presented as mean ± SD from three technical replicates (n = 3), with similar trends observed across at least three independent experiments. **G-H** In vivo limiting dilution assays. BT-549 control or ENO2-KD cells (four cell doses, 1×10⁴-3×10⁶) were orthotopically transplanted into mammary fat pads of NOD-SCID mice. Tumor-initiating frequency was calculated by ELDA. **I** qPCR analysis of stemness-related genes (OCT3/4, SOX2, SOX4) in BT-549 control (2 tumors) and ENO2-KD (3 tumors) xenograft tumors. Data are presented as mean ± SD from three technical replicates of the indicated xenograft tumor samples. **J** Pearson’s correlation between ENO2 and stemness genes in the TCGA breast cancer dataset was analyzed using bc-GenExMiner v4.5. R values and *p*-values are indicated. **K** Immunoblotting analysis of ENO2 in 2D-grown TNBC cells and corresponding mammospheres across multiple cell lines. Representative immunoblots from one of three independent experiments are shown. Statistical significance was determined using unpaired two-tailed Student’s *t*-test where applicable: ns, not significant; *p < 0.05; **p < 0.01; ***p < 0.001.

To further confirm the role of ENO2 for the maintenance of CSC traits, flow cytometric analysis was employed to assess the effect of reduced ENO2 expression on CSC-enriched subpopulations. As illustrated in Fig. 4A, B. CD44⁺/CD24⁻ cells were decreased by 27–42% in BT-549 and 36–41% in MDA-MB-231 spheres, and ALDH⁺ populations were reduced by 76–88% in both cell lines. Extreme limiting dilution assays further demonstrated significantly diminished CSC frequency in ENO2-knockdown and knockout cells (Fig. 4C–E). Consistently, a substantial suppression of secondary sphere formation was observed in ENO2-knockout TNBC cells in a serial sphere-forming assay, a widely used assay for assessing CSC self-renewal. As depicted in Fig. 4F, while control TNBC cells displayed a ~ 2.4-fold increase in secondary mammosphere-forming efficiency, ENO2-knockout cells showed only a modest 1.5-fold enhancement, indicating that ENO2 depletion compromises CSC self-renewal capacity.

To further assess tumor-initiating capacity in vivo, serial dilutions of BT-549 cells were orthotopically transplanted into NOD-SCID mice. ENO2 knockdown reduced tumor-initiating frequency by 20- to 50-fold compared with controls (Control: 1/128,779; shENO2#1: 1/2,396,193; shENO2#3: 1/10,849,469; Fig. 4G, H). Consistently, tumors derived from ENO2-deficient xenografts also showed reduced expression of stemness-associated transcription factors, including SOX4, Oct3/4, and SOX2 (Fig. 4I), without significantly altered expression of E-Cadherin, N-Cadherin, β-catenin and its downstream VEGF (Supplementary Fig. S9). Analysis of TNBC patient datasets further confirmed positive correlations between ENO2 and these CSC regulators (SOX4, R = 0.32; Oct3/4, R = 0.30; SOX2, R = 0.16; Fig. 4J). Notably, cancer spheres derived from multiple TNBC cell lines displayed markedly elevated ENO2 expression, ranging from a ~ 2-fold increase in MDA-MB-231 and HS578T to >16-fold induction in BT-20 and murine 4T1 cells (Fig. 4K). Consistently, ENO2 depletion in 4T1 cells (Supplementary Fig. S10A) recapitulated the phenotypes observed in human TNBC models, leading to a reduction in sphere-forming capacity (Supplementary Fig. S10B) and attenuated tumor growth in vivo (Supplementary Fig. S10C).

In summary, our results indicate a pivotal role for ENO2 in regulating cancer stemness in TNBC and suggest that ENO2 contributes to cancer progression by enhancing metastatic traits, including migration, invasion, and CSC properties, in an EMT-independent manner.

### ENO2 catalytic activity sustains CSC phenotypes through a PEP-PKM2-pyruvate axis and PKM2-independent mechanisms

**Fig. 5:**
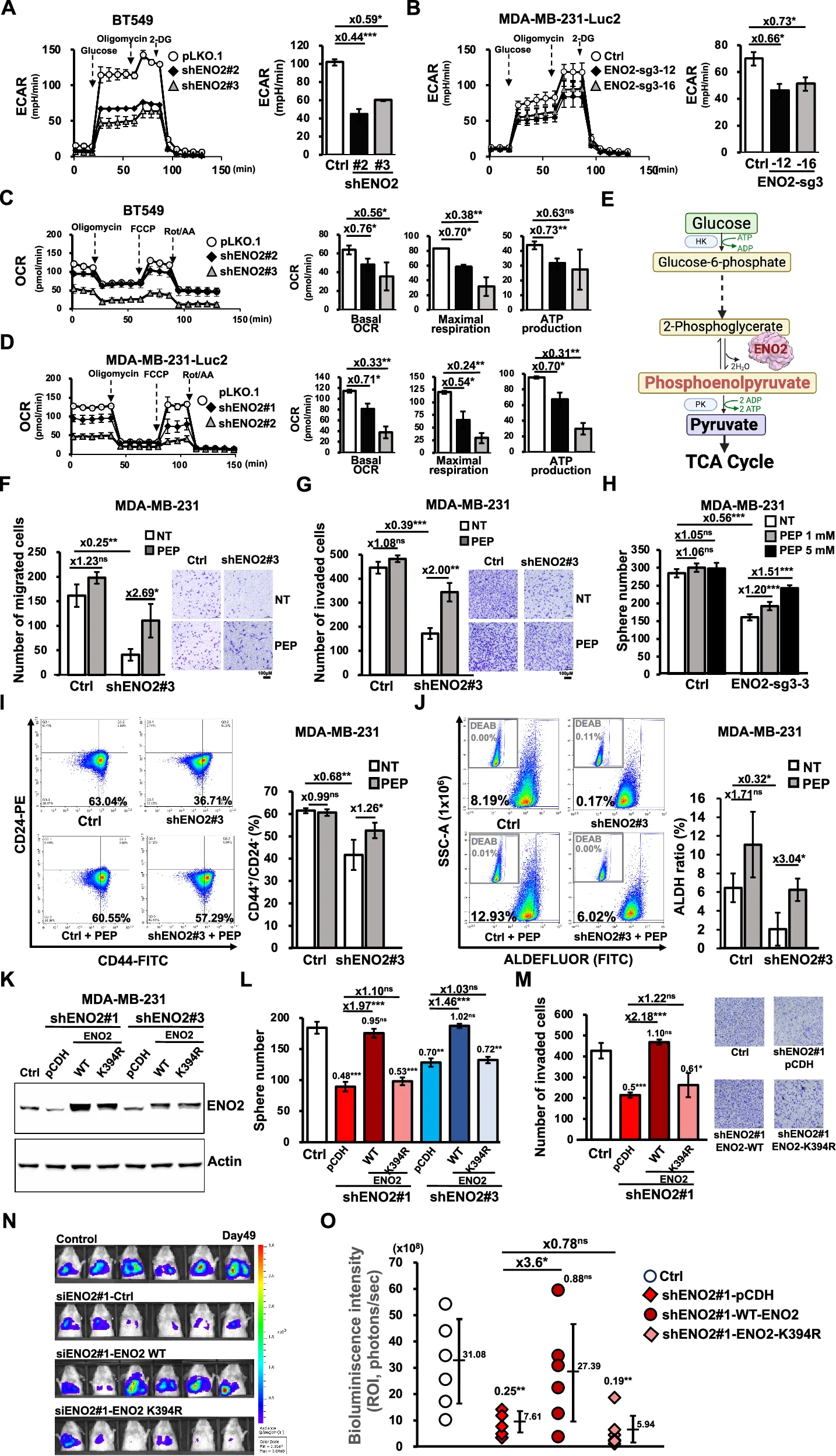
ENO2 regulates migration, invasion, and cancer stemness via its metabolic function. Glycolytic activity was assessed by extracellular acidification rate (ECAR) in ENO2-deficient TNBC cells. BT-549 control and ENO2-KD (shENO2#2 and #3) cells (**A**), and MDA-MB-231 control and ENO2-KO cells (sg3-12, sg3-16) (**B**), were analyzed using Seahorse glycolysis stress assays. ECAR was recorded under basal conditions and after sequential addition of glucose, oligomycin, and 2-DG. Mitochondrial respiration was measured by oxygen consumption rate (OCR) using Seahorse mito-stress assays. BT-549 (**C**) and MDA-MB-231 (**D**) cells with ENO2 knockdown were analyzed under basal conditions and after sequential addition of oligomycin, FCCP, and Rot/AA. Quantification of basal respiration, spare capacity, and ATP-linked respiration is shown. **E** Schematic representation of ENO2-mediated glycolytic conversion of 2-PG to PEP. PEP supplementation (1 mM) restored migration (**F**) and invasion (**G**) capacity in ENO2-deficient MDA-MB-231 cells, as quantified by transwell assays. Representative micrographs and quantification are shown. **H** PEP supplementation restored mammosphere formation in ENO2-deficient MDA-MB-231 cells. Cells were cultured in suspension with 1- or 5-mM PEP for 7–8 days; mammosphere numbers were quantified. PEP supplementation restored CSC-enriched subpopulations in ENO2-deficient mammospheres. Representative flow cytometry plots and quantification of CD44⁺/CD24⁻ (**I**) and ALDH⁺ (**J**) cells are shown. **K** Wild-type ENO2 (ENO2-WT) or the catalytically impaired mutant ENO2-K394R was reintroduced into MDA-MD-231-shENO2 cells. ENO2 expression was confirmed by immunoblotting. Reintroduction of wild-type ENO2, but not ENO2-K394R, restored mammosphere formation (**L**) and invasion capacity (**M**) in ENO2-deficient cells. Pulmonary metastasis assays following lateral tail-vein injection of MDA-MB-231-Luc2 control, ENO2-deficient, wild-type ENO2-reconstituted, or ENO2-K394R-reconstituted cells (1 × 10⁶ cells per mouse; *n* = 6 mice per group). Metastatic burden was quantified by in vivo bioluminescence imaging (IVIS). Representative images (**N**) and chest bioluminescence intensities (**O**) are shown. For **A**–**H,** **L**, **M**, data are shown from one representative experiment and presented as mean ± SD from three technical replicates (*n* = 3). Similar trends were observed across at least three independent experiments. For **I**, **J**, flow cytometry quantification is presented as mean ± SD from three independent biological replicates (*n* = 3 independent experiments). For panel **O**, data are presented as mean ± SD from six mice per group. Statistical significance was determined using unpaired two-tailed *t*-tests: ns, not significant; *p < 0.05; **p < 0.01; ***p < 0.001.

As ENO2 catalyzes the conversion of 2-phosphoglycerate to phosphoenolpyruvate (PEP) during glycolysis, we next examined whether its enzymatic activity underlies its role in TNBC progression. Suppression of ENO2 significantly reduced glycolytic activity, as reflected by decreased extracellular acidification rates (ECAR) measured by Seahorse analysis (Fig. 5A, B and Supplementary Fig. S11A). ENO2 depletion also impaired OXPHOS, with basal and ATP-linked oxygen consumption rate (OCR) reduced by 30–70% (Fig. 5C, D and Supplementary Fig. S11B), indicating that ENO2-driven glycolysis supports not only lactate production but also mitochondrial respiration.

Because PEP is the immediate product of ENO2 and is subsequently converted to pyruvate during glycolysis (Fig. 5E), we examined whether PEP contributes to ENO2-dependent biological effects. Supplementation of ENO2-deficient TNBC cells with exogenous PEP markedly restored their migratory (Fig. 5F and Supplementary Fig. S12) and invasive capacities (Fig. 5G). Moreover, PEP supplementation also rescued mammosphere formation in ENO2-deficient cells in a dose-dependent manner, with no effect in controls. As shown in Fig. 5H, treatment with 5 mM PEP increased sphere-forming efficiency of ENO2-KO MDA-MB-231 cells by ~1.5-fold, achieving a level comparable to parental controls (control vs. PEP-treated ENO2-KO: 1:0.85; control vs. untreated ENO2-KO: 1:0.56). A similar 1.68-fold rescue was observed in ENO2-KD BT-549 cells (Supplementary Fig. S13).

Consistently, PEP treatment reinstated both CD44⁺/CD24⁻ and ALDH⁺ CSC-like populations in ENO2-deficient MDA-MB-231 (Fig. 5I, J) and BT-549 cells (Supplementary Fig. S14), supporting the involvement of PEP in ENO2-dependent CSC-associated phenotypes.

To directly determine whether ENO2 catalytic activity is required for its biological effects, wild-type ENO2 or the catalytically impaired K394R mutant [6] was reintroduced into ENO2-KD (Fig. 5K and Supplementary Fig. S15A) and ENO2-KO cells (Supplementary Fig. S16A). Consistent with the results shown in Fig. 3B, neither ENO2 deficiency nor reconstitution with wild-type ENO2 or the K394R mutant substantially affected cell proliferation, as assessed by direct cell counting (Supplementary Fig. S15A). In parallel, no detectable cleavage of PARP-1 or caspase-3 was observed among these cells (Supplementary Fig. S15B), indicating that ENO2 loss does not markedly induce apoptosis under these conditions. Moreover, expression of β−catenin and its downstream target Cyclin D1 remained largely unchanged (Supplementary Fig. S15B), which, together with the direct cell-counting results, suggests that ENO2 loss is unlikely to cause overt proliferative or cell-cycle perturbation. These findings indicate that ENO2 catalytic activity is not required for general cell growth or survival under these conditions, allowing us to further assess its specific contribution to CSC-associated and metastatic phenotypes.

In contrast, ENO2 catalytic activity was clearly required for CSC-associated and metastatic phenotypes. Reconstitution with wild-type ENO2, but not the K394R mutant, restored mammosphere-forming ability (Fig. 5K, L and Supplementary Fig. S16B) and invasion (Fig. 5M and Supplementary Fig. S16C). Importantly, this catalytic requirement was further substantiated in vivo, as wild-type ENO2, but not the K394R mutant, restored pulmonary metastatic colonization by ENO2-deficient cells, increasing lung bioluminescence intensity to a level comparable to that of control cells (Fig. 5N, O). Together, these findings indicate that ENO2 catalytic activity is not required for general cell growth or survival, but is specifically required for CSC-associated sphere formation, invasion, and pulmonary metastatic colonization.

**Fig. 6:**
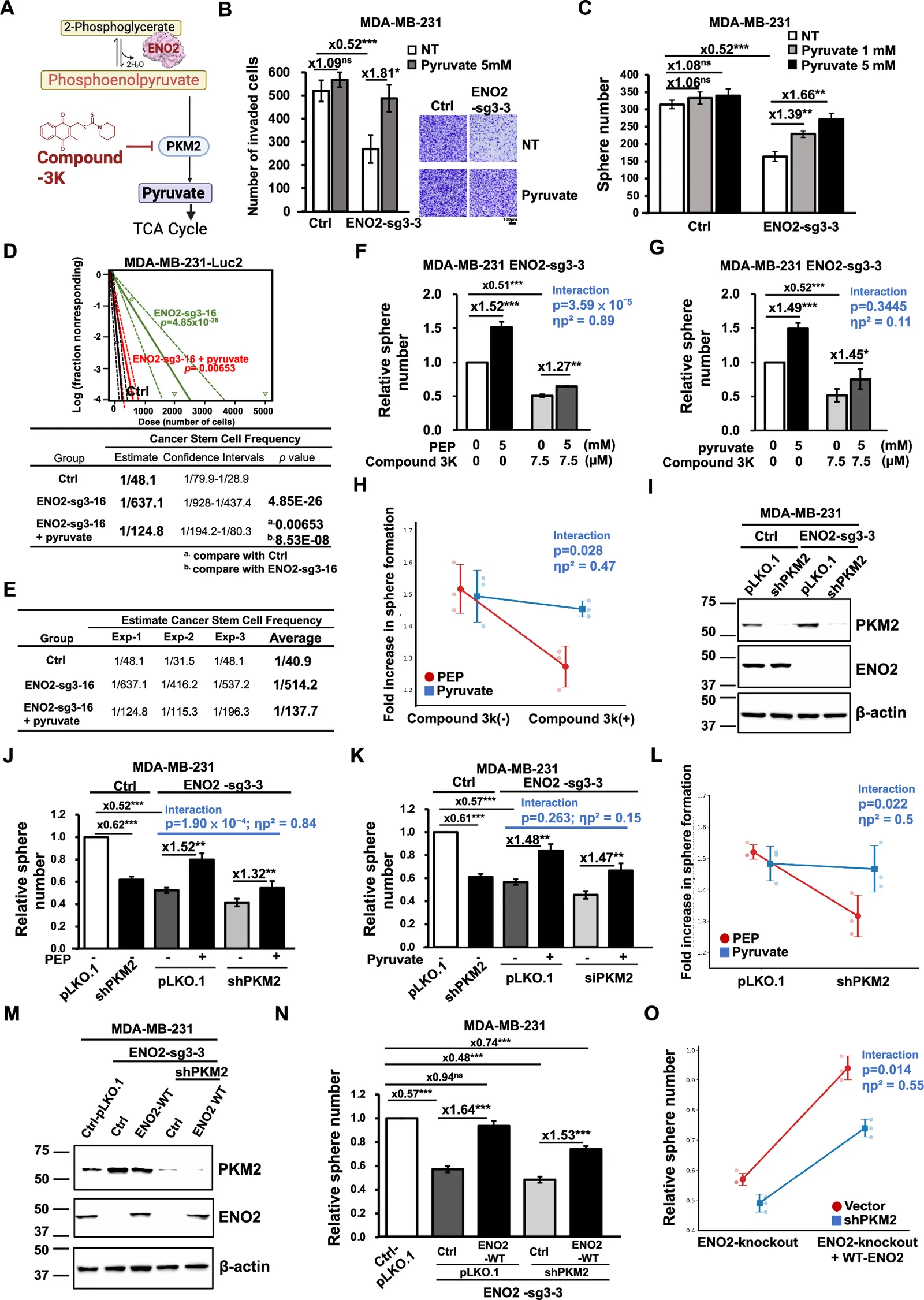
PEP-derived Pyruvate contributes to ENO2-mediated regulation of cancer stemness. **A** Schematic illustration of Phosphoenolpyruvate (PEP) conversion to pyruvate via pyruvate kinase M2 (PKM2). This enzymatic step can be inhibited by the small-molecule inhibitor compound 3 K. Control (Ctrl) and ENO2-knockout MDA-MB-231 cells (ENO2-sg3-3) were treated with pyruvate (0, 1, or 5 mM), respectively, for 7 days and subjected to trans-well invasion (**B**) and mammosphere formation assay (**C**). Fold changes relative to Ctrl are indicated above the bars. **D** Limiting dilution assay (ELDA) of MDA-MB-231-Luc2 Control (Ctrl), ENO2-knockout cells (ENO2-sg3-16), and pyruvate (1 mM)-treated ENO2-knockout cells. Data are plotted as log fraction of non-responding wells versus plated cell number. Estimated CSC frequency and 95% confidence intervals are provided. **E** Summary of CSC frequency derived from three independent ELDA experiments shown in (**D)**, with group averages shown. ENO2-sg3-3 cells were subjected to mammosphere assays in the presence of PEP (**F**) or pyruvate (**G**), with or without compound 3 K. Relative sphere-forming efficiency was calculated by normalizing each group to untreated ENO2-sg3-3 cells. PEP-mediated rescue was significantly attenuated by PKM2 inhibition (**F**; interaction p = 3.59 × 10⁻⁵; ηp² = 0.89), whereas pyruvate-mediated effects were largely unaffected (**G**; p = 0.3445; ηp² = 0.11). **H** Interaction plot derived from (**F, G**) showing fold changes in sphere-forming efficiency following PEP or pyruvate treatment with or without compound 3 K. Two-way ANOVA revealed a significant metabolite × PKM2 inhibition interaction (p = 0.028; ηp² = 0.47). **I** Immunoblot analysis of ENO2 and PKM2 expression in control and ENO2-knockout MDA-MB-231 cells with or without PKM2 knockdown. This setup was used for subsequent functional assays in the figures. ENO2-sg3-3 cells with or without PKM2 knockdown were subjected to mammosphere assays in the presence or absence of PEP (**J**) or pyruvate (**K**). Relative sphere-forming efficiency was calculated by normalizing each group to untreated Ctrl-pLKO.1 cells. PEP-mediated rescue was significantly reduced upon PKM2 depletion (**J**; interaction *p* = 1.90 × 10⁻⁴; ηp² = 0.84), whereas pyruvate-mediated effects were largely unchanged (**K**; *p* = 0.263; ηp² = 0.15). **L** Interaction plot derived from (**J, K)** showing fold changes in sphere-forming efficiency following PEP or pyruvate treatment with or without PKM2 knockdown. Two-way ANOVA revealed a significant metabolite × PKM2 depletion interaction (*p* = 0.022; ηp² = 0.50). **M** Immunoblot analysis confirming PKM2 knockdown and wild-type ENO2 reconstitution in ENO2-sg3-3 cells. PKM2 was depleted prior to reintroduction of wild-type ENO2, and the resulting cell models were used for mammosphere assays in (**N, O)**. β-actin served as a loading control. **N** ENO2-sg3-3 cells with or without PKM2 knockdown were reconstituted with wild-type ENO2 and subjected to mammosphere assays. Relative sphere-forming efficiency was calculated by normalizing each group to untreated Ctrl-pLKO.1 cells. ENO2 reconstitution restored sphere formation in PKM2-intact cells but only partially rescued sphere formation under PKM2 depletion. **O** Interaction plot derived from (**N**) showing the combined effects of ENO2 reconstitution and PKM2 depletion on sphere-forming efficiency. Two-way ANOVA revealed a significant ENO2 reconstitution × PKM2 depletion interaction (*p* = 0.014; ηp² = 0.55). For **B, C** and representative images, data are shown from one representative experiment and presented as mean ± SD from three technical replicates (*n* = 3). Similar trends were observed across at least three independent experiments. For **D,E**CSC frequency was derived from three independent ELDA experiments. For **F–O**, data are presented as mean ± SD from three independent biological replicates (*n* = 3 independent experiments), with each biological replicate representing an independently performed experiment. Pairwise comparisons are indicated by asterisks: ns, not significant; *p < 0.05; **p < 0.01; ***p < 0.001.

Given that PEP is subsequently converted to pyruvate by PKM2 during glycolysis (Fig. 6A), we next tested whether pyruvate could phenocopy the effects of PEP in ENO2-deficient cells. Pyruvate treatment dose-dependently increased invasion in ENO2-KO cells (Fig. 6B) and enhanced both mammosphere-forming efficiency (Fig. 6C) and CSC frequency (Fig. 6D, E). Notably, pyruvate substantially restored CSC frequency in ENO2-deficient cells, increasing it from 1/514.2 in untreated ENO2-KO cells to 1/137.7 after pyruvate treatment (Fig. 6E), thereby partially restoring the CSC frequency reduced by ENO2 deficiency.

As both PEP and pyruvate restored CSC-associated phenotypes in ENO2-deficient cells, we next investigated whether the effects of PEP depend on its conversion to pyruvate through PKM2. Pharmacologic inhibition of PKM2 with compound 3 K [27] attenuated PEP-mediated rescue of sphere formation, whereas pyruvate-mediated rescue remained largely unaffected (Fig. 6F, G).

To quantify metabolite-induced effects, fold-change analysis (treated/untreated within each PKM2 condition) was performed. PEP supplementation increased sphere-forming efficiency by approximately 1.52-fold, which was reduced to ~1.27-fold upon PKM2 inhibition, whereas pyruvate-mediated rescue showed only minimal change (~1.49- to ~1.45-fold). Two-way ANOVA of fold-change values revealed a significant interaction between metabolite treatment and PKM2 inhibition (*p* = 0.028; ηp² = 0.47; Fig. 6H and Supplementary Fig. S17A), indicating preferential sensitivity of PEP-mediated rescue to PKM2 perturbation.

Similar results were obtained using genetic depletion of PKM2 (Fig. 6I). PKM2 knockdown reduced PEP-mediated rescue (~1.52- to ~1.32-fold) but had minimal impact on pyruvate-mediated rescue (~1.48- to ~1.47-fold) (Fig. 6J, K). Two-way ANOVA of fold-change values again confirmed a significant interaction between metabolite treatment and PKM2 depletion (*p* = 0.022; ηp² = 0.5; Fig. 6L and Supplementary Fig. S17B), consistent with preferential attenuation of PEP-mediated, but not pyruvate-mediated, rescue by PKM2 depletion. These findings indicate that the ability of PEP to promote CSC phenotypes is at least partially dependent on its conversion to pyruvate through PKM2, while pyruvate functions downstream of PKM2 as an effector metabolite.

To further assess the contribution of PEP-to-pyruvate conversion to ENO2-mediated CSC regulation, PKM2 was depleted prior to reconstitution of wild-type ENO2 in ENO2-deficient cells (Fig. 6M). ENO2 reconstitution largely restored sphere formation in PKM2-intact cells (~0.57 to ~0.94 relative to control), but only partially restored sphere formation under PKM2 depletion (~0.48 to ~0.74), remaining below control levels (Fig. 6N).

This differential response was further visualized using an interaction plot based on relative sphere-forming efficiency values derived from independent experiments (Fig. 6O), in which non-parallel trajectories indicated that the effect of ENO2 reconstitution varied depending on PKM2 status. Two-way ANOVA confirmed a significant ENO2 × PKM2 interaction (*p* = 0.014; ηp² = 0.55), indicating that ENO2 function is partially dependent on PKM2 activity. Moreover, the persistence of residual rescue under PKM2 perturbation further suggests that a PKM2-independent mechanism may also be involved in ENO2-dependent CSC phenotypes.

Collectively, these findings indicate that PEP contributes to ENO2-dependent CSC phenotypes through both a PEP-PKM2-pyruvate axis and an additional PKM2-independent PEP-responsive route.

### Targeting ENO2 by POMHEX suppresses cell growth, cancer stemness, and in vivo tumor growth

POMHEX is a small-molecule enolase inhibitor with approximately fourfold higher affinity for ENO2 than ENO1 and exhibits potent nanomolar activity against ENO1-deficient glioma [10], highlighting ENO2 as a potential therapeutic vulnerability. Given the marked upregulation of ENO2 in TNBC spheres and the profound suppression of CSC traits upon ENO2 loss, we evaluated the therapeutic efficacy of POMHEX in TNBC models.

**Fig. 7:**
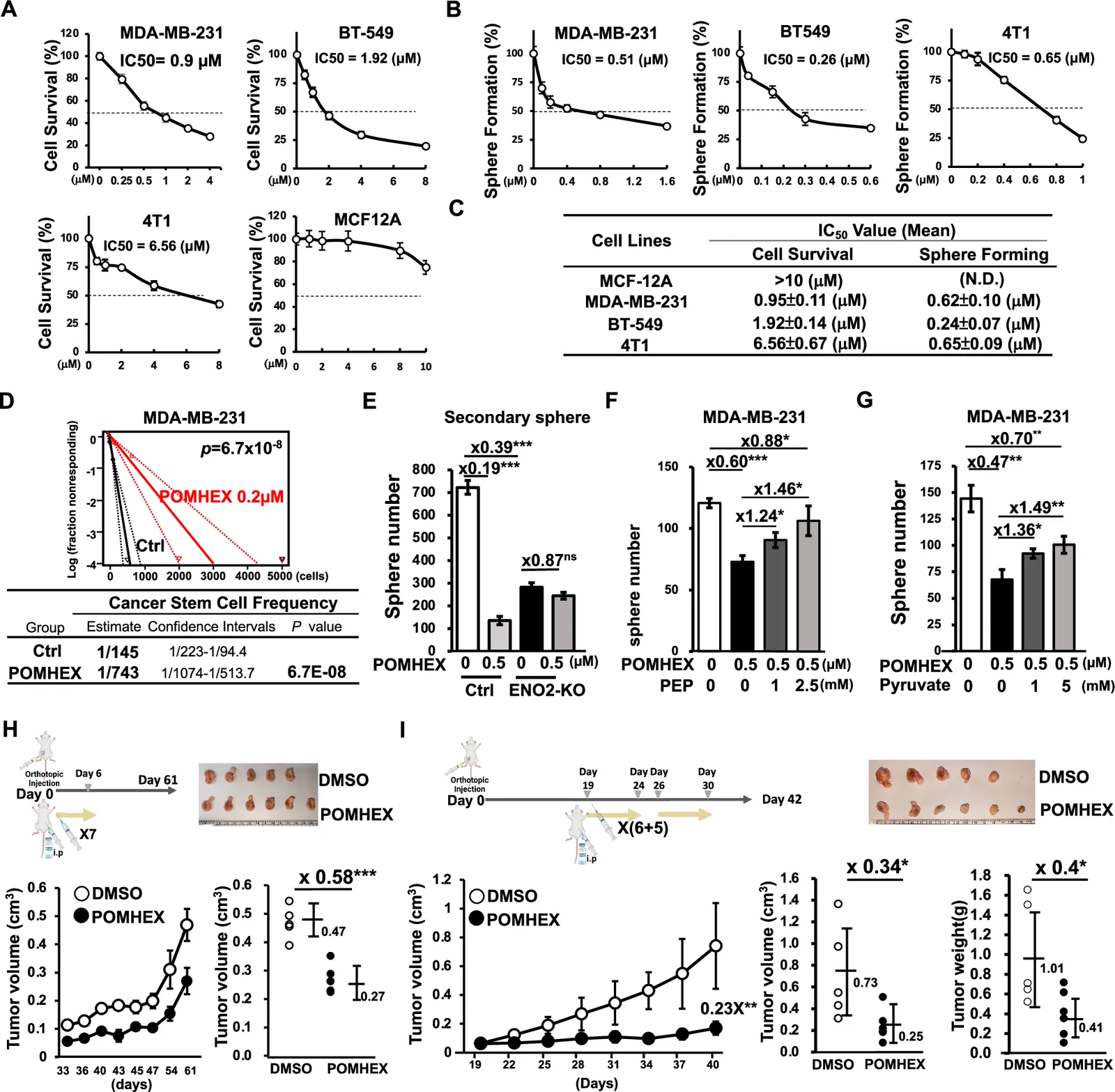
Therapeutic effect of POMHEX in TNBC. IC₅₀ values of POMHEX in TNBC cell survival assays (**A**) and primary sphere formation assays(**B**). **C**. Summary of IC₅₀ values from three independent experiments shown in (**A, B). D** POMHEX reduces CSC frequency in TNBC cells. Upper: representative images of mammospheres treated with POMHEX (0.2 μM). Lower: CSC frequency calculated using ELDA, with estimated frequencies and p-values shown in the table. **E** POMHEX suppresses CSC self-renewal in an ENO2-dependent manner. Primary spheres from control and ENO2-knockout MDA-MB-231 cells were treated with or without POMHEX (0.5 μM) and assessed in secondary sphere formation assays. Fold-change values are indicated. Exogenous PEP (**F**) or pyruvate (**G**) alleviates POMHEX-mediated suppression of sphere formation. MDA-MB-231 cells were treated with POMHEX (0.5 μM) alone or in combination with PEP (1 and 2.5 mM, **F**) or pyruvate (1 and 5 mM, **G**). Fold-change values are shown above each bar. **H** POMHEX delays tumorigenesis in vivo. MDA-MB-231 cells were orthotopically implanted into the mammary fat pads of NOD-SCID mice. Mice received POMHEX (25 mg/kg, *n* = 6) or vehicle (2% DMSO in PBS, *n* = 5) intraperitoneally from day 0 for 7 consecutive days. Tumor growth curves (left) and endpoint tumor volumes (right) are shown. **I** POMHEX suppresses established tumor growth in vivo. NOD-SCID mice bearing orthotopic MDA-MB-231 tumors (~0.1 cm³) were randomized to receive vehicle (*n* = 5) or POMHEX (25 mg/kg, *n* = 6) for 11 days. Tumor growth kinetics, final tumor volumes, tumor weights, and representative endpoint xenograft images are shown. For **A, B**, **D–G**, data are shown from one representative experiment and presented as mean ± SD from three technical replicates (*n* = 3). Similar trends were observed across at least three independent experiments. For **C**, data are presented as mean ± SD from three independent biological replicates (*n* = 3 independent experiments). For **H, I**, data are presented as mean ± SD from the indicated numbers of mice per group. Statistical significance was determined using an unpaired two-tailed Student’s *t*test where applicable: ns, not significant; *p < 0.05; **p < 0.01; ***p < 0.001.

POMHEX effectively suppressed TNBC cell proliferation, with IC₅₀ values of 0.897 μM (MDA-MB-231), 1.92 μM (BT-549), and 6.56 μM (4T1). Sphere cultures displayed heightened sensitivity, with IC₅₀ values of 0.51 μM, 0.26 μM, and 0.65 μM (Fig. 7A–C). Consistent with ENO2’s role in CSC maintenance, POMHEX significantly reduced CSC frequency (Fig. 7D) and impaired secondary mammosphere formation (Fig. 7E).

Importantly, the inhibitory effect of POMHEX on sphere formation was abolished in ENO2-knockout cells (Fig. 7E), confirming ENO2 as the primary functional target mediating POMHEX sensitivity. Supplementation with either PEP or pyruvate restored mammosphere-forming capacity in POMHEX-treated cells (Fig. 7F, G), further supporting that POMHEX acts by disrupting ENO2-dependent metabolic flux.

Next, the in vivo efficacy of POMHEX was evaluated in orthotopic TNBC murine models. POMHEX, administered concurrently with tumor implantation and delivered daily for 7 days (30 mg/kg, i.p.), significantly reduced primary tumor formation (Fig. 7H). Additionally, POMHEX was effective against established disease, with an 11-day treatment course substantially attenuating tumor growth, as demonstrated by slower growth kinetics, reduced tumor volumes, and decreased tumor weights at endpoint (Fig. 7I).

Together, these findings demonstrate that pharmacologic ENO2 inhibition disrupts stemness-associated phenotypes and exerts significant anti-tumor activity in vivo. The dual ability of POMHEX to impair tumor initiation and suppress established disease underscores ENO2 as a therapeutically relevant metabolic dependency in TNBC and supports the therapeutic potential of ENO2-targeted metabolic intervention.

## Discussion

Metabolic reprogramming is a hallmark of cancer and enables tumor cells to adapt to diverse bioenergetic and biosynthetic demands during disease progression [28, 29]. In addition to altered glycolytic flux, tumors display substantial metabolic heterogeneity, allowing cancer cells to flexibly engage distinct metabolic programs in response to microenvironmental stress. While elevated aerobic glycolysis supports rapid proliferation by supplying intermediates for macromolecule synthesis, multiple cancer types, including melanoma, pancreatic cancer, and TNBC, also exhibit a dependency on mitochondrial OXPHOS during metastatic progression. Accumulating evidence further indicates that both glycolysis and OXPHOS contribute to the regulation of CSC states, underscoring the complexity of metabolic control over tumor initiation, progression, and therapy resistance [29–31].

In this study, we investigated the clinical and functional relevance of ENO2 in TNBC and provided evidence supporting a link between its enzymatic activity and tumor progression. Elevated ENO2 expression was associated with advanced tumor grade, but not tumor stage, in our tissue-based TNBC cohort, and was correlated with worse clinical outcomes across two independent public TNBC cohorts, including OS, DFS, and DMFS. In our tissue cohort, tumor grades ranged from 1 to 3, whereas tumor stages ranged from IA to IIIB, with all cases classified as M0. This staging range, particularly the absence of distant metastatic disease, may limit the ability to detect associations between ENO2 expression and tumor stage. Accordingly, the lack of correlation with stage should be interpreted with caution, as it may reflect cohort composition rather than the absence of a biological relationship.

By contrast, tumor grade reflects the degree of cellular differentiation and malignancy, whereas tumor stage represents the anatomical extent of disease progression [32]. In this context, the observed association between ENO2 expression and tumor grade is consistent with a role for ENO2 in tumor cell-intrinsic properties associated with cancer stem cell phenotypes, which are often linked to cellular plasticity. Similar observations have been reported for metabolic regulators such as GLUT1, whose expression has been associated with higher tumor grade, TNBC subtype, proliferative activity, and poorer clinical outcome in breast cancer [33–36]. Notably, several studies reported that GLUT1 expression was not significantly associated with TNM-related parameters, including tumor size and lymph node status [35, 36]. In addition, a recent tissue-based study found no significant variation in GLUT1 expression across clinical stages I–IV, despite its association with higher tumor grade, TNBC subtype, and Ki-67 proliferative index [37]. Together with the survival associations observed in two independent public TNBC cohorts, these findings support the clinical relevance of ENO2 as a marker linked to CSC-associated malignant phenotypes, while highlighting the need for further validation in larger cohorts with complete survival and staging annotation.

At the same time, the tissue microarray analysis has several limitations. Although ENO2 staining intensity was quantified using ImageJ/Fiji-based DAB color deconvolution and mean optical density-based tertile classification, this approach still reduces continuous and spatially heterogeneous staining patterns into categorical groups. ImageJ/Fiji-based color deconvolution provides an objective measurement of DAB staining intensity and reduces observer-dependent bias compared with purely visual scoring. However, more advanced digital pathology workflows that support spatially resolved or cell-level quantification may provide a more accurate and objective assessment of staining heterogeneity in tumor samples. Such approaches would further help determine the biological and pathological significance of heterogeneous ENO2 staining patterns in TNBC tissues.

Consistent with the clinical associations, our in vivo experiments further support a functional role for ENO2 in tumor growth and pulmonary metastasis and showed that ENO2 depletion prolongs survival in murine metastatic models. The concordance between clinical associations and functional observations supports ENO2 as a potential contributor to tumor aggressiveness. Future validation in larger patient cohorts with complete clinical annotation and the use of patient-derived xenografts would further strengthen the clinical and translational significance of ENO2.

Functionally, ENO2 appears to regulate metastatic traits in TNBC through maintenance of CSC properties. Downregulation of ENO2 significantly reduced migration and invasion without altering canonical EMT markers (Fig. 3D–H), suggesting that ENO2 may promote metastatic competence through EMT-independent mechanisms. In parallel, ENO2 deficiency reduced mammosphere formation, self-renewal capacity, CSC-enriched subpopulations (CD44⁺/CD24⁻ and ALDH⁺), and tumor-initiating potential. Together, these observations provide a functional basis for the association between ENO2 expression and tumor grade, supporting a link between ENO2 and tumor cell-intrinsic malignant phenotypes.

Although ENO2 exhibits non-metabolic “moonlighting” functions in several malignancies, including activation of AKT-GSK3β [38], PI3K/AKT and MAPK/ERK [39], Hippo-YAP1 signaling [40], as well as stabilization of PKM2 to promote CCND1-driven proliferation in head and neck squamous cell carcinoma (HNSCC) [9], our study supports an important role for its catalytic activity in CSC regulation in TNBC. Reconstitution experiments showed that wild-type ENO2, but not the catalytically impaired K394R mutant, restored CSC traits, invasion, and metastatic capacity, indicating that ENO2-mediated regulation of cancer stemness depends on its enzymatic activity. Consistently, supplementation with PEP or pyruvate restored CSC phenotypes in ENO2-deficient cells, supporting the functional involvement of ENO2-associated metabolites in these processes.

Further mechanistic analyses suggest the involvement of a PEP-PKM2-pyruvate axis in ENO2-mediated CSC regulation. Pharmacologic inhibition or genetic depletion of PKM2 attenuated PEP-mediated rescue of CSC phenotypes, whereas pyruvate-mediated restoration remained largely preserved. These observations indicate that a significant portion of ENO2-dependent CSC regulation may be mediated through PKM2-dependent conversion of PEP to pyruvate. Consistently, ENO2-driven restoration of CSC phenotypes was partially dependent on PKM2 activity, collectively suggesting a functional relationship between ENO2 enzymatic activity and PKM2-dependent pyruvate production.

**Fig. 8:**
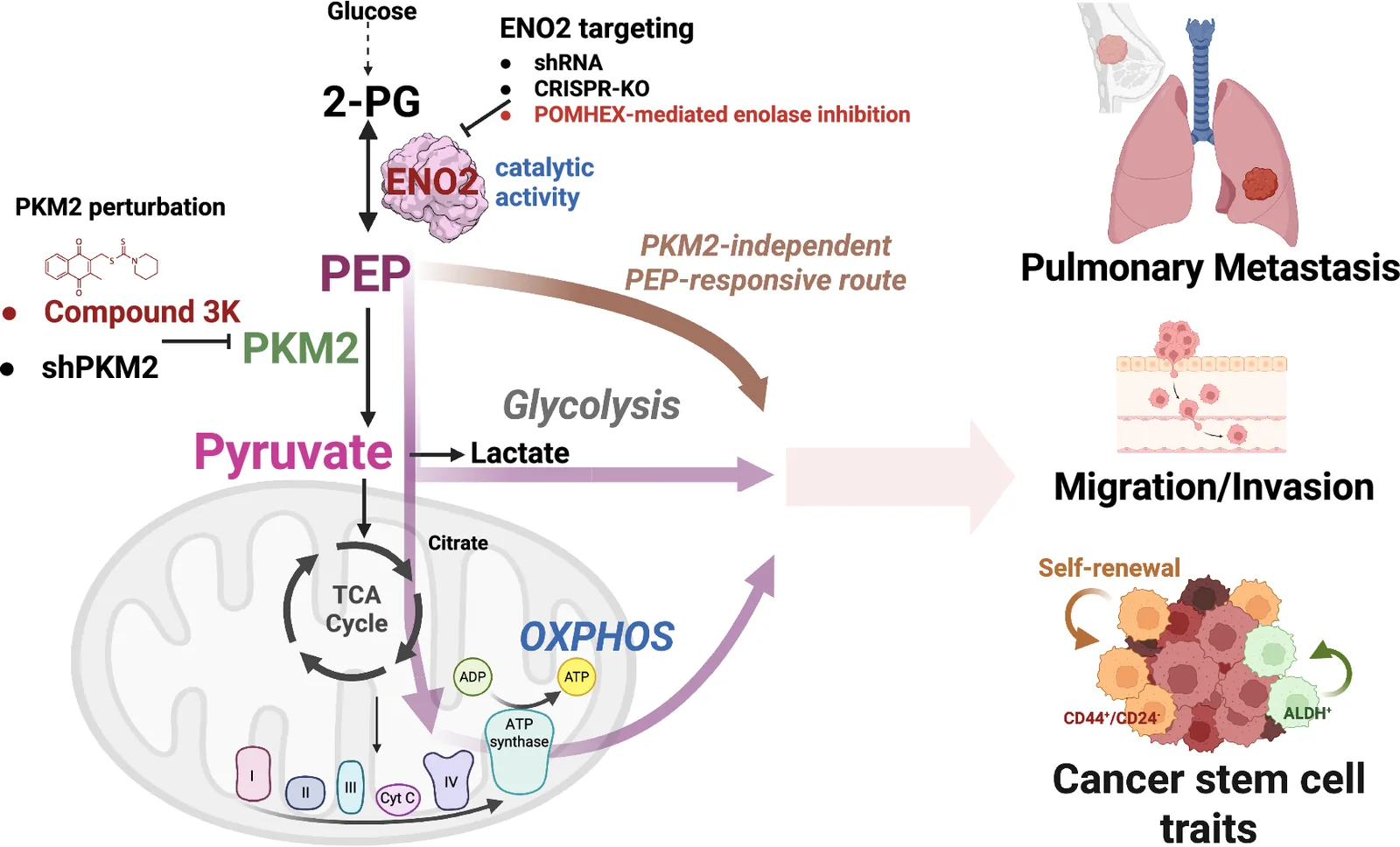
Proposed model of the ENO2-driven PEP-dependent metabolic axis linking metabolic reprogramming to cancer stemness and metastasis in TNBC. ENO2 catalyzes the conversion of 2-phosphoglycerate (2-PG) to phosphoenolpyruvate (PEP), thereby supporting PEP-responsive metabolism. Genetic targeting of ENO2 by shRNA or CRISPR-mediated knockout, as well as POMHEX-mediated enolase inhibition, suppresses this ENO2-dependent metabolic pathway. ENO2-derived PEP can be converted to pyruvate through PKM2, a step functionally perturbed by compound 3 K or shPKM2. Pyruvate further supports lactate production and mitochondrial metabolism through the TCA cycle and oxidative phosphorylation (OXPHOS). In addition to the PEP-PKM2-pyruvate route, the incomplete suppression of CSC-associated phenotypes under PKM2 perturbation suggests the presence of an additional PKM2-independent PEP-responsive route. These PKM2-dependent and PKM2-independent routes may converge one metabolic support for cancer stem cell traits, migration/invasion, and pulmonary metastatic competence in TNBC. Illustration created with BioRender.com.

In this context, pyruvate supplementation restored CSC phenotypes in ENO2-deficient cells and largely bypassed PKM2 perturbation, indicating pyruvate as a downstream effector metabolite. As a central metabolic intermediate, pyruvate can be converted to lactate or enter the tricarboxylic acid (TCA) cycle to support OXPHOS. This metabolic positioning aligns with the observed impairment of both glycolysis and OXPHOS upon ENO2 depletion. Together, these findings suggest that ENO2 may link CSC regulation with cellular metabolism through the PEP-PKM2-pyruvate axis (Fig. 8).

Although our functional analyses support the involvement of the ENO2-PEP-PKM2–pyruvate axis in cancer stemness regulation, the endogenous metabolic changes underlying this model remain to be fully defined. In the present study, metabolite rescue, PKM2 perturbation, and catalytic rescue experiments demonstrated that PEP-dependent metabolic regulation functionally contributes to ENO2-associated CSC phenotypes. However, because intracellular PEP and pyruvate levels were not directly measured, we cannot determine whether ENO2 deficiency alters the endogenous abundance of these metabolites or whether such changes contribute to impaired CSC maintenance. Future studies using direct metabolomic quantification and isotope-tracing approaches would help define how endogenous PEP and pyruvate pools are altered, routed, and utilized following ENO2 loss. Such analyses would further clarify how PEP/pyruvate metabolism is integrated into CSC regulation and deepen the mechanistic understanding of how the ENO2-PEP-PKM2-pyruvate axis contributes to cancer stemness and metastatic competence.

Within this framework, the proposed model is compatible with the metabolic heterogeneity of TNBC CSCs, in which CD44⁺/CD24⁻ populations preferentially rely on glycolysis, whereas ALDH⁺ populations depend on mitochondrial respiration [41]. Consistently, supplementation with PEP restored both CSC subpopulations, including the ALDH⁺ population, suggesting that ENO2-associated metabolism may support multiple energetic states required for CSC maintenance.

Beyond PKM2-dependent regulation, the incomplete suppression of CSC phenotypes under PKM2 perturbation suggests the presence of an additional PKM2-independent PEP-responsive route. These observations indicate that ENO2-mediated metabolic regulation is not exclusively dependent on PKM2 activity and may involve alternative metabolic routing or metabolite-dependent regulatory processes. Future studies will be required to identify these PKM2-independent mechanisms and determine whether they contribute to CSC maintenance, metastatic colonization, or therapeutic vulnerability.

Furthermore, our additional analyses suggest that the CSC-associated defects observed following ENO2 depletion are unlikely to be explained by alterations in apoptosis, cell-cycle progression, or proliferation. ENO2 depletion did not induce detectable PARP-1 or caspase-3 cleavage, arguing against overt apoptosis as a major contributor. In addition, unchanged Cyclin D1 expression, together with direct cell-counting results, suggests that ENO2 loss does not cause substantial proliferative or cell-cycle perturbation under these conditions. Nevertheless, these analyses do not exclude the possibility that ENO2 may influence other regulated cell death or stress-survival pathways under distinct biological or metabolic contexts. Indeed, ENO2 interference has been reported to promote ferroptosis in clear cell renal cell carcinoma, linking ENO2-dependent glycolytic and mitochondrial functions to ferroptosis sensitivity [40]. Therefore, although apoptosis was not detectably induced in our TNBC models under standard culture conditions, ENO2 loss-induced metabolic impairment may still influence other forms of regulated cell death under specific environmental stresses, such as glucose limitation, oxidative stress, anchorage-independent growth, or metastatic colonization. Future studies examining ferroptosis, oxidative-stress-associated cell death, and other regulated cell death pathways under these metabolically challenging conditions will be required to determine whether ENO2 supports tumor progression not only by maintaining CSC-associated metabolic fitness, but also by protecting tumor cells from context-dependent cell death during metastatic dissemination and colonization.

In addition to its well-known metabolic role, PEP has been reported to exert non-metabolic regulatory functions through modulation of histone deacetylase 1 (HDAC1). In colorectal cancer, ENO2-derived PEP was reported to inhibit HDAC1-mediated β-catenin deacetylation, thereby enhancing β-catenin-dependent transcriptional programs and consequent resistance to antiangiogenic therapy [42]. However, in our TNBC system, ENO2 depletion did not alter β-catenin expression or the expression of its downstream targets, including Cyclin D1 and VEGF, suggesting that the PEP-HDAC1-β-catenin axis is unlikely to represent a major contributing mechanism in this context.

In vivo, ENO2 depletion suppressed primary tumor growth, spontaneous pulmonary metastasis, and tumor-associated mortality in orthotopic xenograft models. In parallel, tail-vein injection experiments showed that ENO2 depletion also reduced lung colonization and prolonged survival. Because the tail-vein injection model bypasses primary tumor angiogenesis and early steps of the invasion-metastasis cascade, including local invasion and intravasation, while assessing later steps such as survival in circulation, extravasation, and colonization, these findings support the interpretation that ENO2 influences pulmonary metastasis at least in part at the metastatic colonization stage. Nevertheless, this experimental design does not exclude a potential contribution of angiogenesis or vascular remodeling during metastatic outgrowth in the lung. Future studies incorporating angiogenic analyses will be required to clarify whether ENO2-derived PEP contributes to tumor vascularization in TNBC.

Given its functional role in CSC maintenance and metastasis, ENO2 represents a potential therapeutic vulnerability in TNBC. We therefore evaluated the ENO2-selective inhibitor POMHEX, which exhibits fourfold higher specificity for ENO2 over ENO1 and has demonstrated anti-tumor effects in ENO1-deficient glioma [10]. In TNBC, POMHEX suppressed sphere formation, CSC frequency, and self-renewal, with effects absent in ENO2-deficient cells and reversible by PEP and pyruvate supplementation, supporting the metabolic basis of ENO2-dependent CSC regulation. Importantly, POMHEX reduced orthotopic TNBC tumor initiation and suppressed the growth of established tumors, demonstrating in vivo efficacy. These findings highlight the therapeutic potential of targeting ENO2-dependent metabolic vulnerability in TNBC.

In summary, our study identifies ENO2 as a metabolic and functional regulator of TNBC progression. ENO2 expression correlates with poor prognosis in public TNBC cohorts, and functional studies support its role in CSC maintenance and metastasis. Mechanistically, our findings support a functionally relevant PEP-dependent metabolic axis involving PKM2-dependent PEP-to-pyruvate conversion and an additional PKM2-independent PEP-responsive route. Although direct metabolomic and isotope-tracing analyses will be required to fully resolve endogenous PEP/pyruvate abundance and flux, our data support a model in which ENO2 catalytic activity contributes to CSC-associated sphere formation, invasion, and pulmonary metastatic colonization. These findings reveal ENO2-dependent metabolic regulation as a potential vulnerability in TNBC.

## Supplementary information

supplementary figures

supplementary table

Uncropped images

## Data Availability

All data files supporting the findings of this study are available upon reasonable request. No newly generated datasets requiring accession codes. No custom code central to the conclusions was generated. Public TNBC cohort analyses were performed using bc-GenExMiner v4.8, Kaplan–Meier Plotter, TNMplot, and GENT2. Experimental data generated in this study are available from the corresponding author upon reasonable request.
