# Supplementary material for: ENO2 sustains cancer stemness and metastatic competence through a phosphoenolpyruvate-dependent metabolic axis in triple-negative breast cancer
Source: Cell Death Dis. 2026 Jul 11;17(1):864. doi: 10.1038/s41419-026-09106-0 (PMC13639003; doi:10.1038/s41419-026-09106-0)
Supplement: Supplementary file 1 [file 41419_2026_9106_MOESM1_ESM.pdf]

Supplementary Figure S1

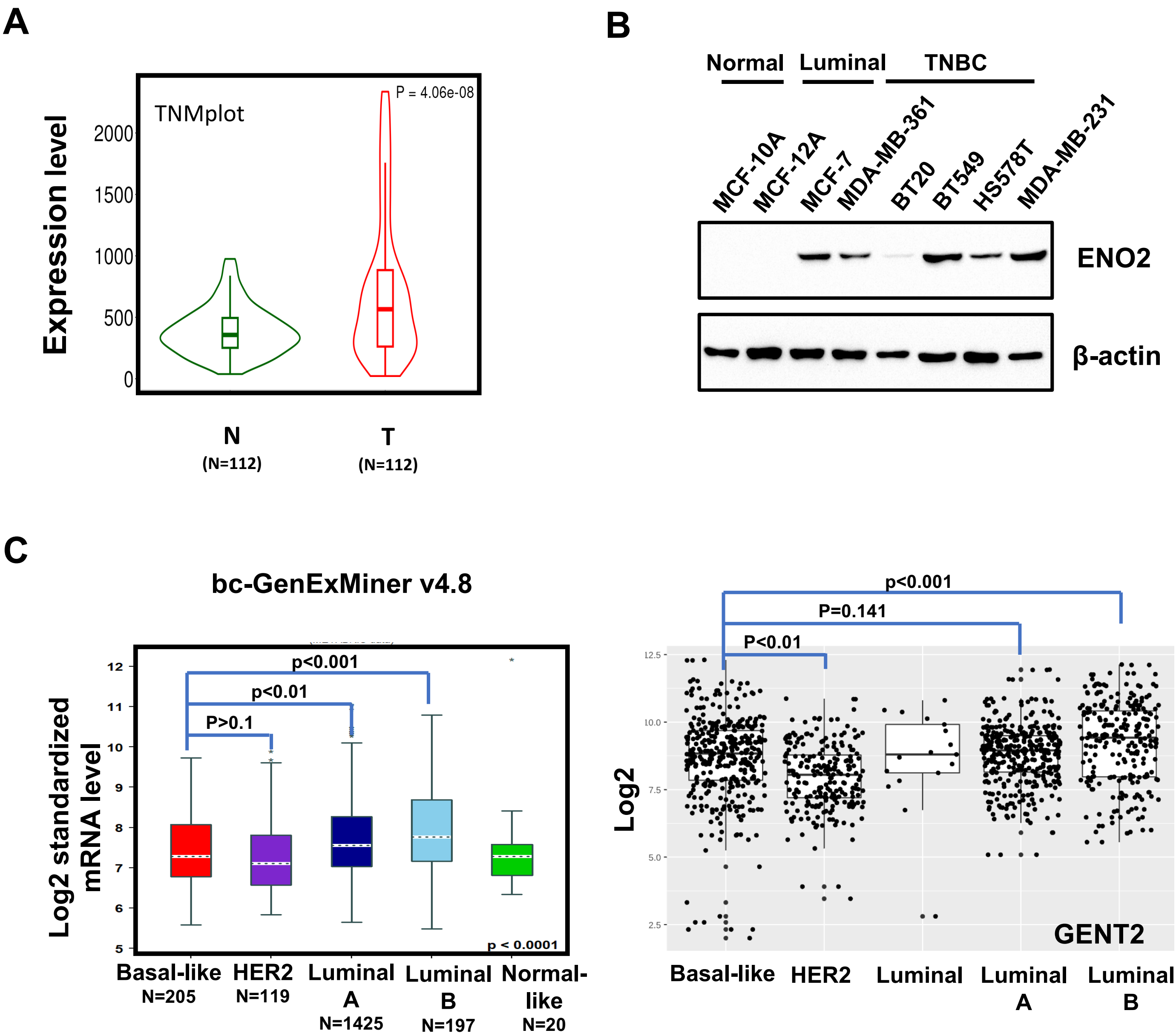

Supplementary Figure S1. Elevated ENO2 expression in breast cancers. A. ENO2 mRNA expression in paired adjacent-normal and tumor breast tissue samples was analyzed using the TNMplot online database (n = 122 per group). N, adjacent normal; T, tumor. **B.** ENO2 protein levels were examined by immunoblotting in normal mammary epithelial cell lines (MCF-10A, MCF-12A), luminal breast cancer cell lines (MCF-7, MDA-MB-361), and triple-negative breast cancer (TNBC) cell lines (BT-20, BT-549, Hs578T, MDA-MB-231). **C.** ENO2 mRNA expression across intrinsic molecular subtypes of breast cancer was analyzed using bc-GenExMiner v4.8 (left panel; METABRIC dataset, PAM50 subtypes) and GENT2 (right panel).

Supplementary Figure S2

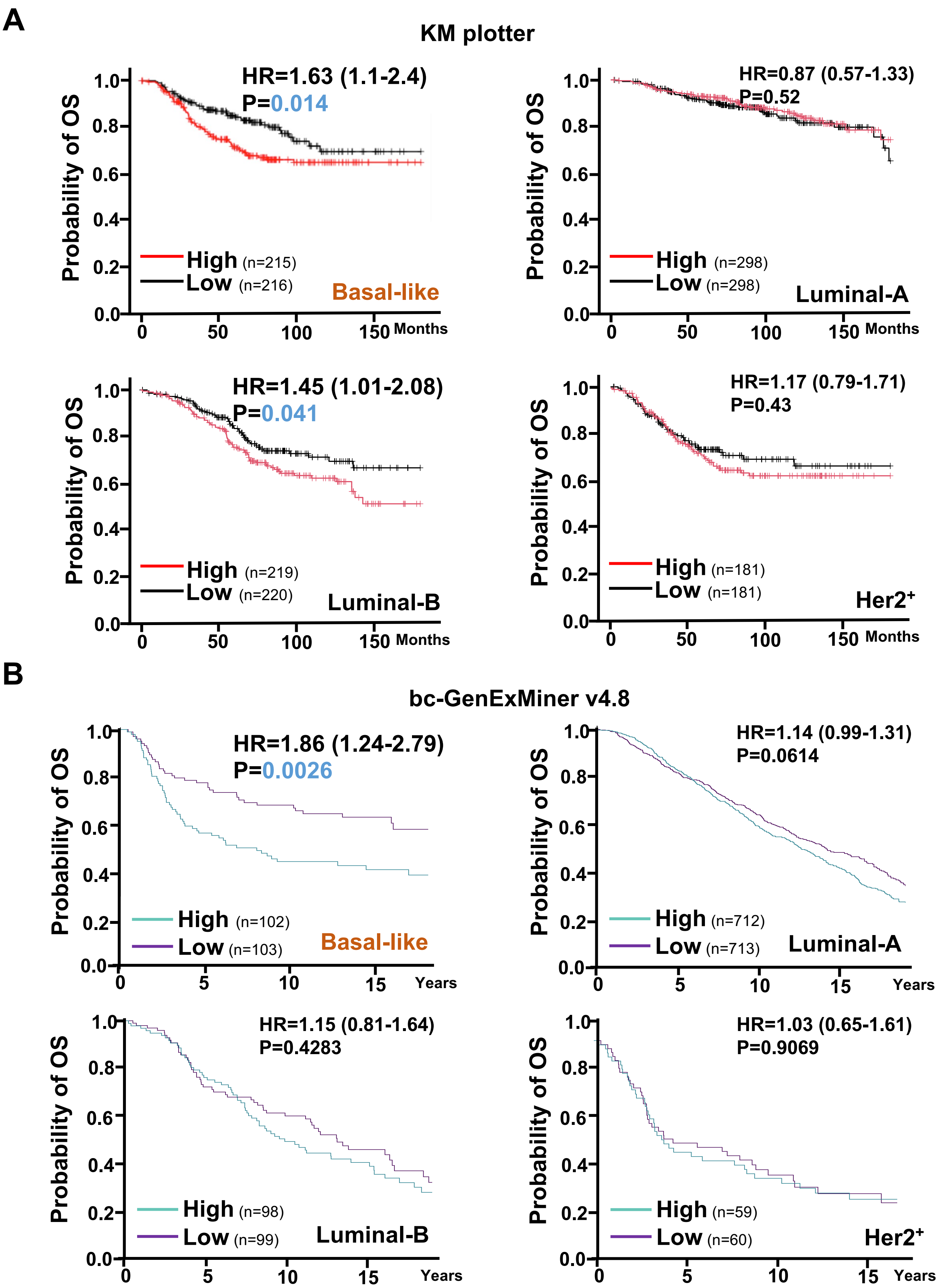

Supplementary Figure S2. ENO2 predicts poor overall survival in patients with basal-like breast cancer. Overall survival (OS) of breast cancer patients was analyzed using (**A**) the microarray dataset via KM plotter and (**B**) the METABRIC dataset via bc-GenExMiner v4.8. Molecular subtypes were classified by the PAM50 gene signature, and patients were stratified into ENO2-high and ENO2-low cohorts according to the median expression threshold. Statistical significance of survival differences was evaluated using the log-rank test.

Supplementary Figure S3

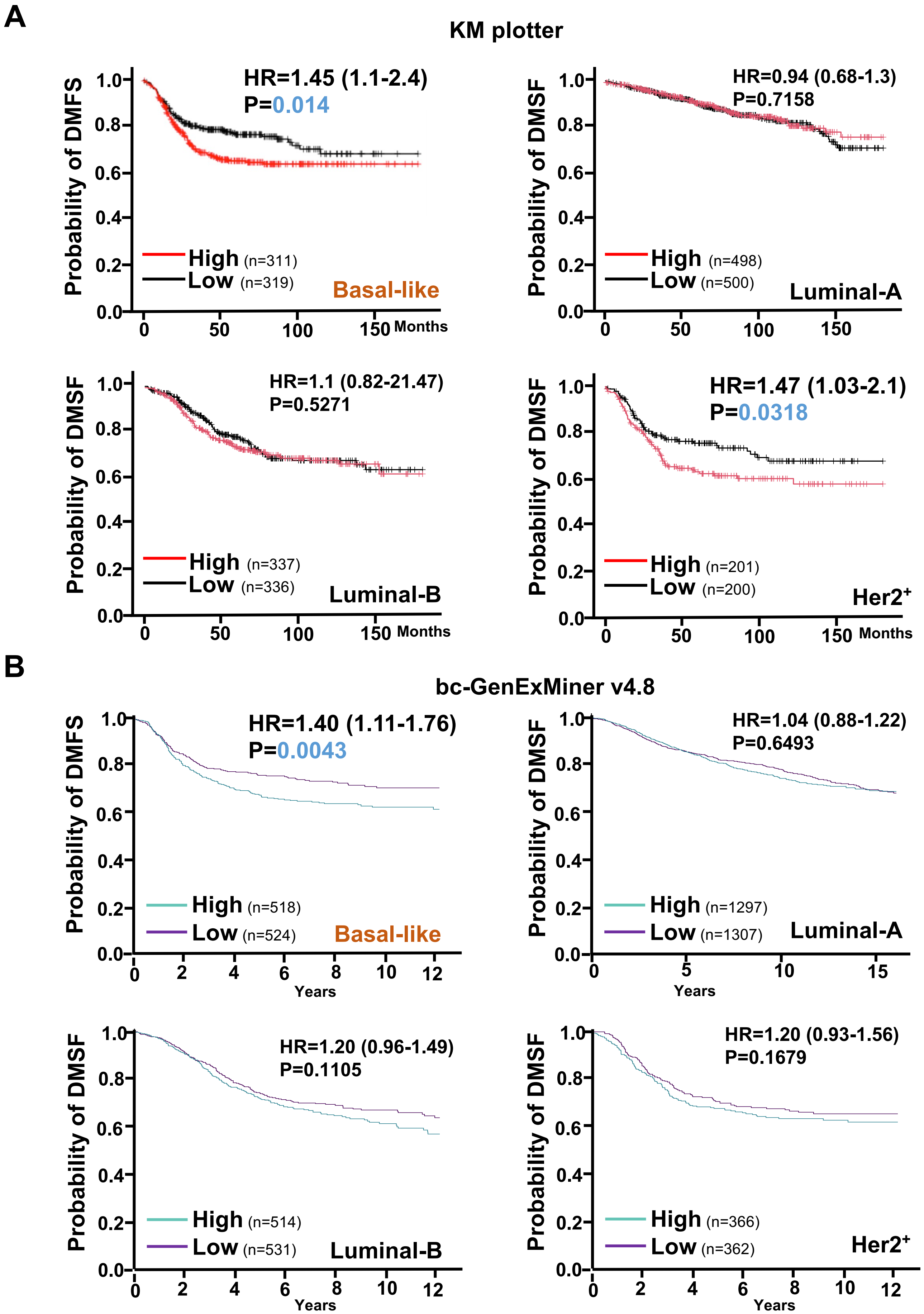

Supplementary Figure S3. ENO2 predicts poor distant metastasis–free survival in patients with basal-like breast cancer. Distant metastasis–free survival (DMFS) of breast cancer patients was analyzed using (**A**) the microarray dataset via KM plotter and (**B**) all DNA microarray datasets via bc-GenExMiner v4.8. Molecular subtypes of breast cancer were classified by the PAM50 gene signature, and patients were stratified into ENO2-high and ENO2-low cohorts based on the median expression threshold.

Supplementary Figure S4

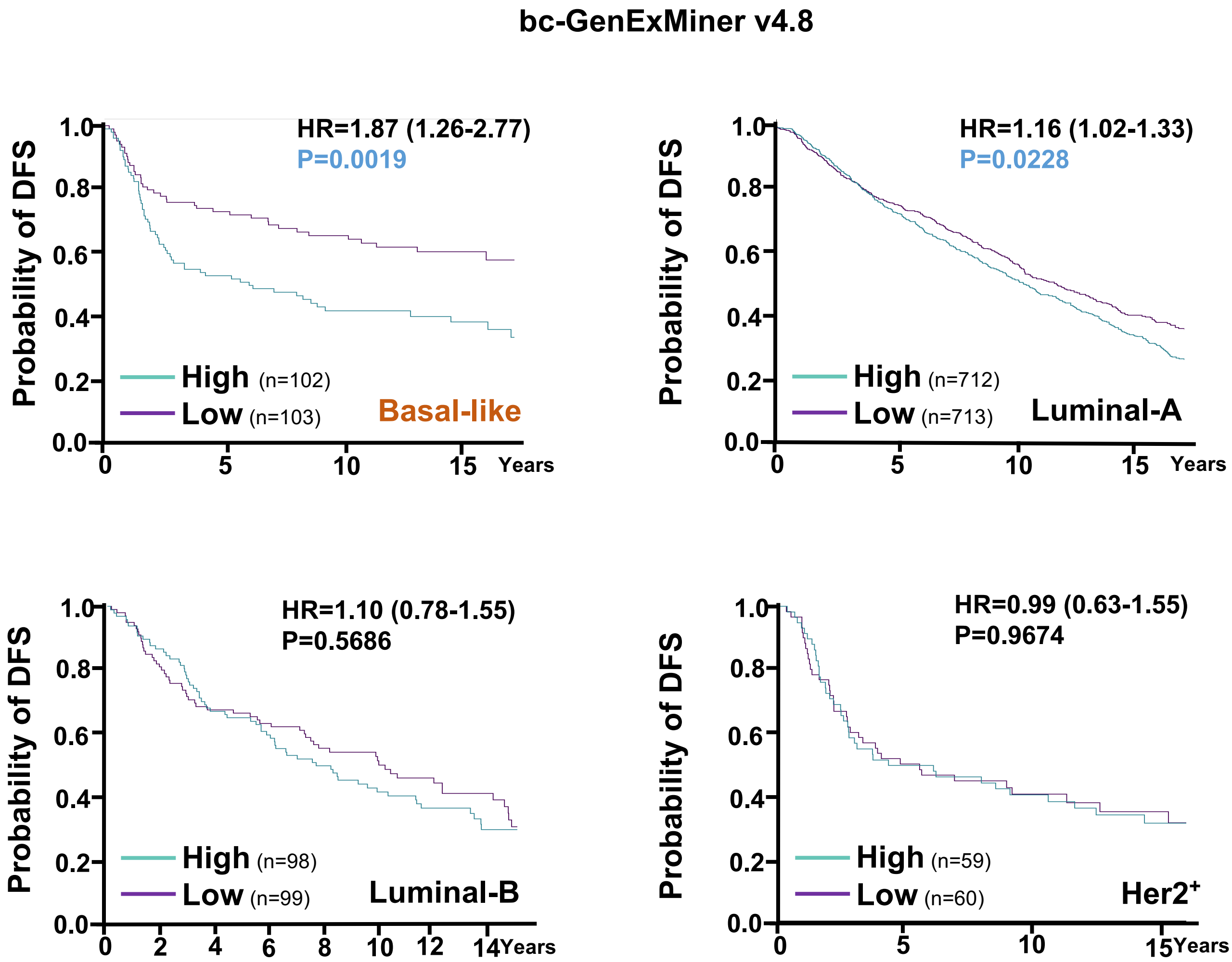

Supplementary Figure S4. ENO2 predicts poor disease-free survival in patients with basal-like breast cancer. Disease-free survival (DFS) of breast cancer patients from the METABRIC dataset was analyzed using the bc-GenExMiner v4.8 online tool. Molecular subtypes of breast cancer were classified by the PAM50 gene signature, and patients were stratified into ENO2-high and ENO2-low cohorts based on the median expression threshold. Statistical significance of survival differences was evaluated using the log-rank test.

Supplementary Figure S5

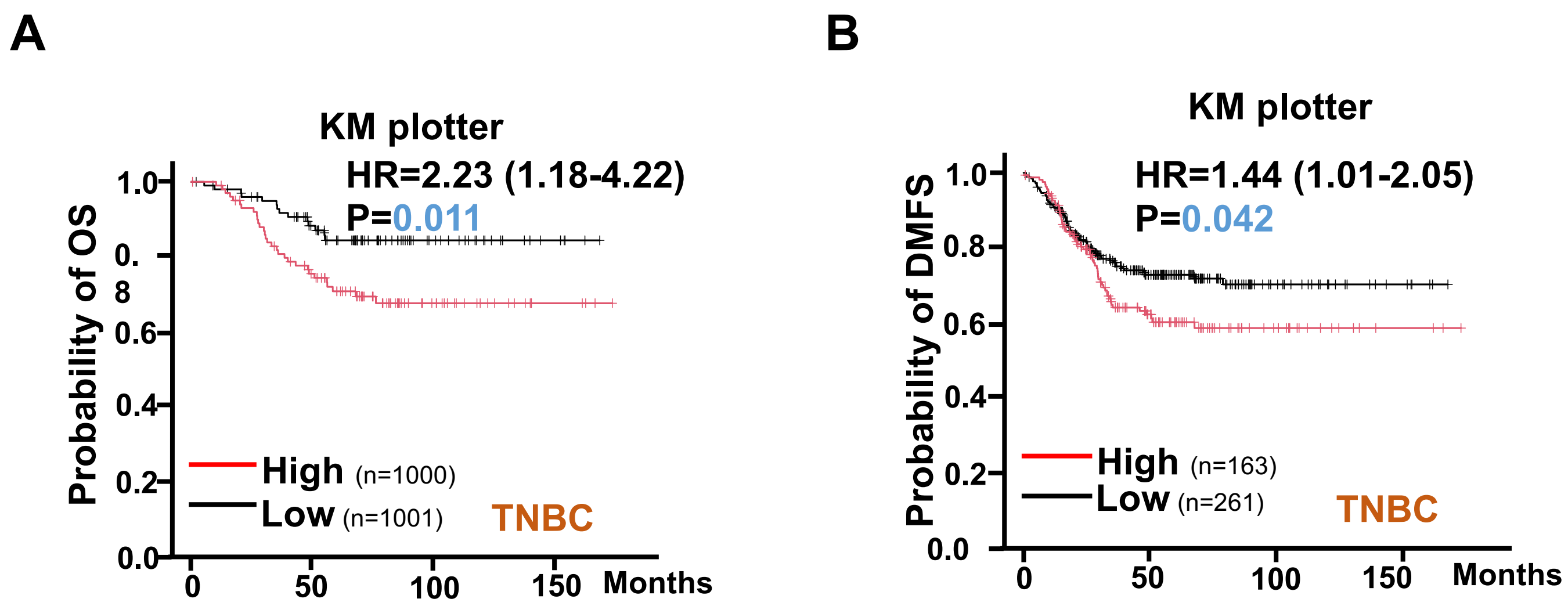

Supplementary Figure S5. ENO2 predicts poor survival in TNBC patients. (**A**) Overall survival (OS) and (**B**) distant metastasis-free survival (DMFS) of TNBC patients were analyzed using the KM plotter online tool. Patients were stratified into ENO2-high and ENO2-low cohorts based on ENO2 expression levels in the microarray dataset. Statistical significance of survival differences was evaluated using the log-rank test.

Supplementary Figure S6

A

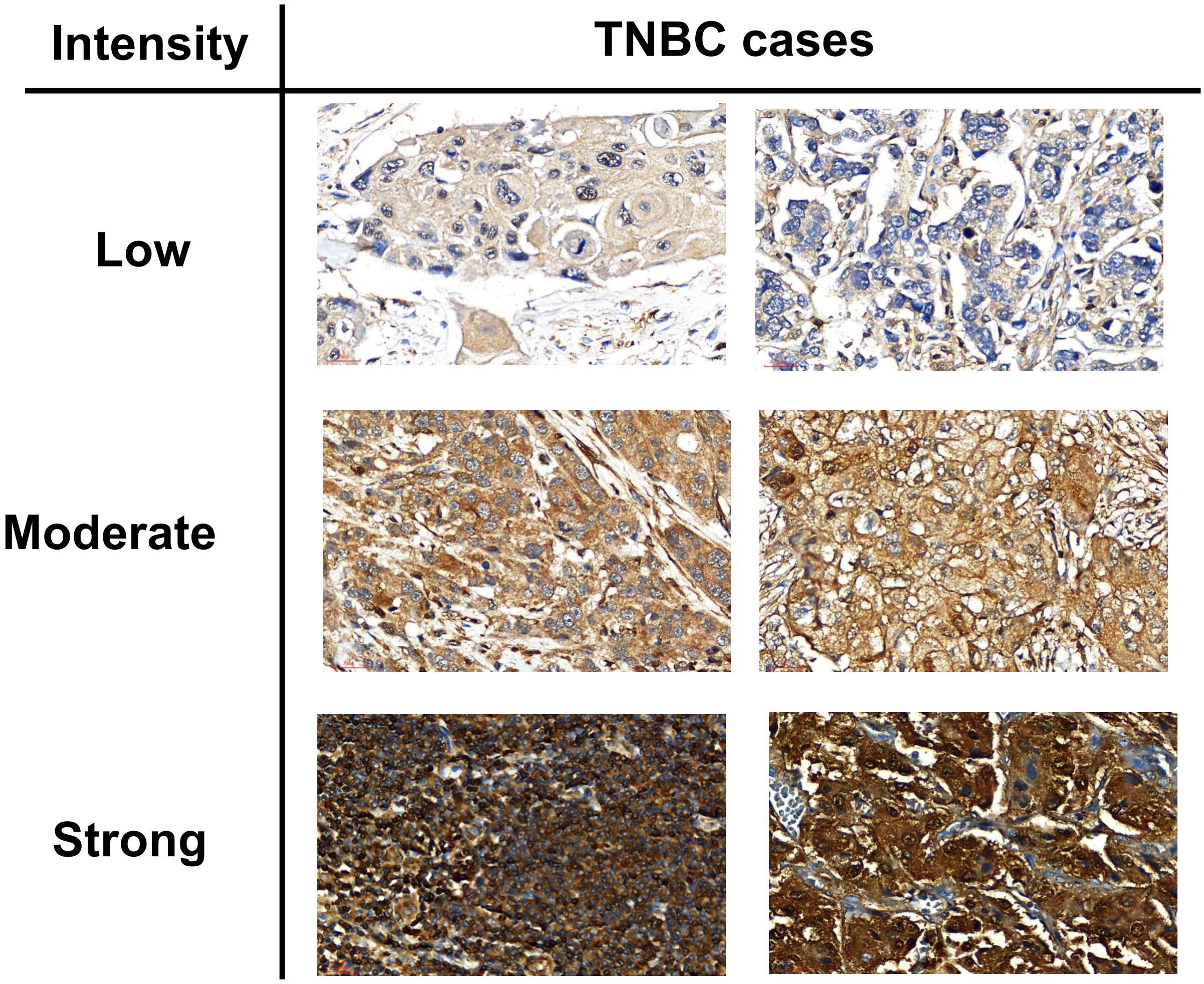

B

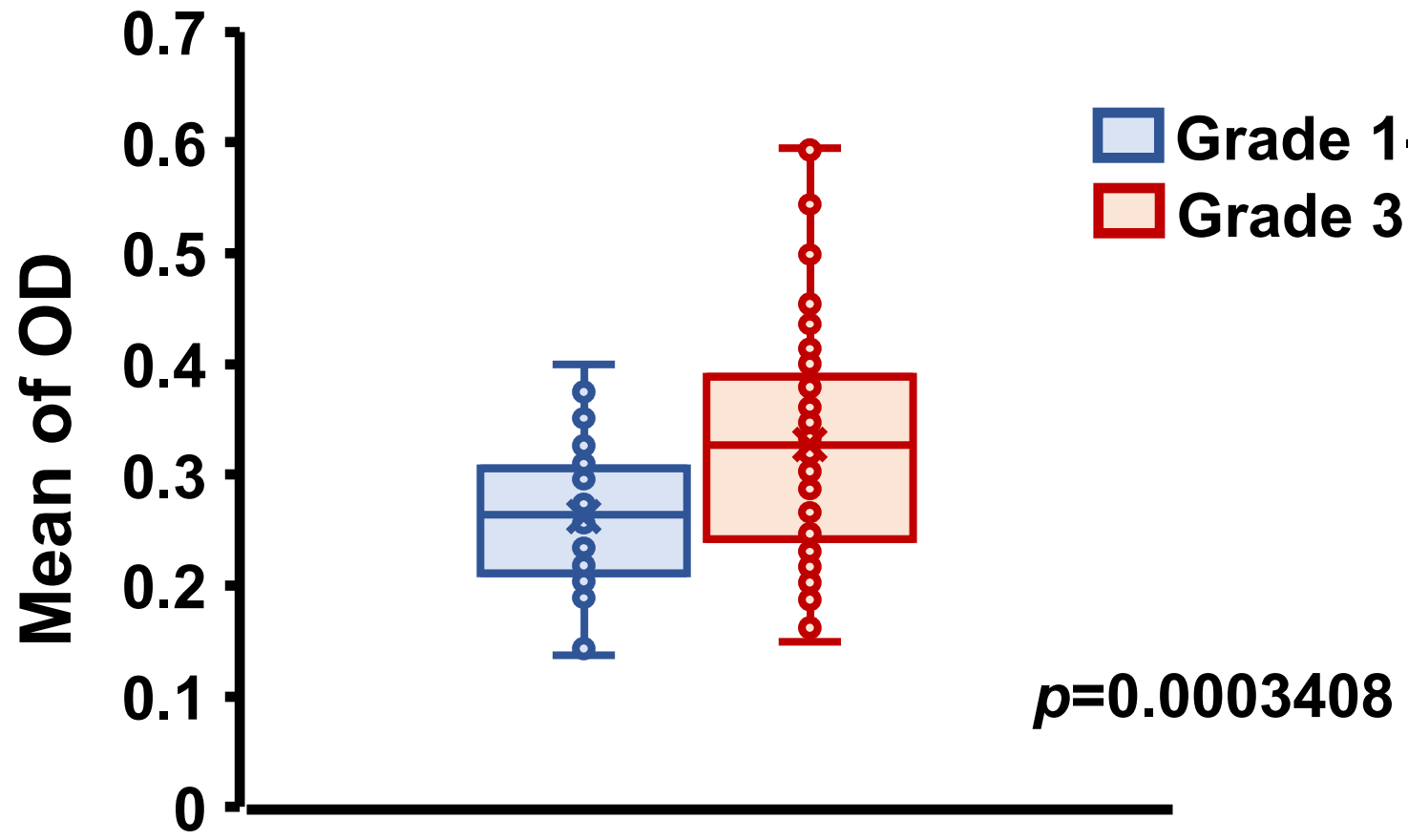

C

Staining Intensity	Tumor Grade 1+2	Tumor Grade 3	Row totals
Negative/Weak	19	21	40
Moderate	19	21	40
Strong	5	35	40
Column totals	43	77	120

Chi-Square Test *P*=0.0008

Supplementary Figure 6 continued

D

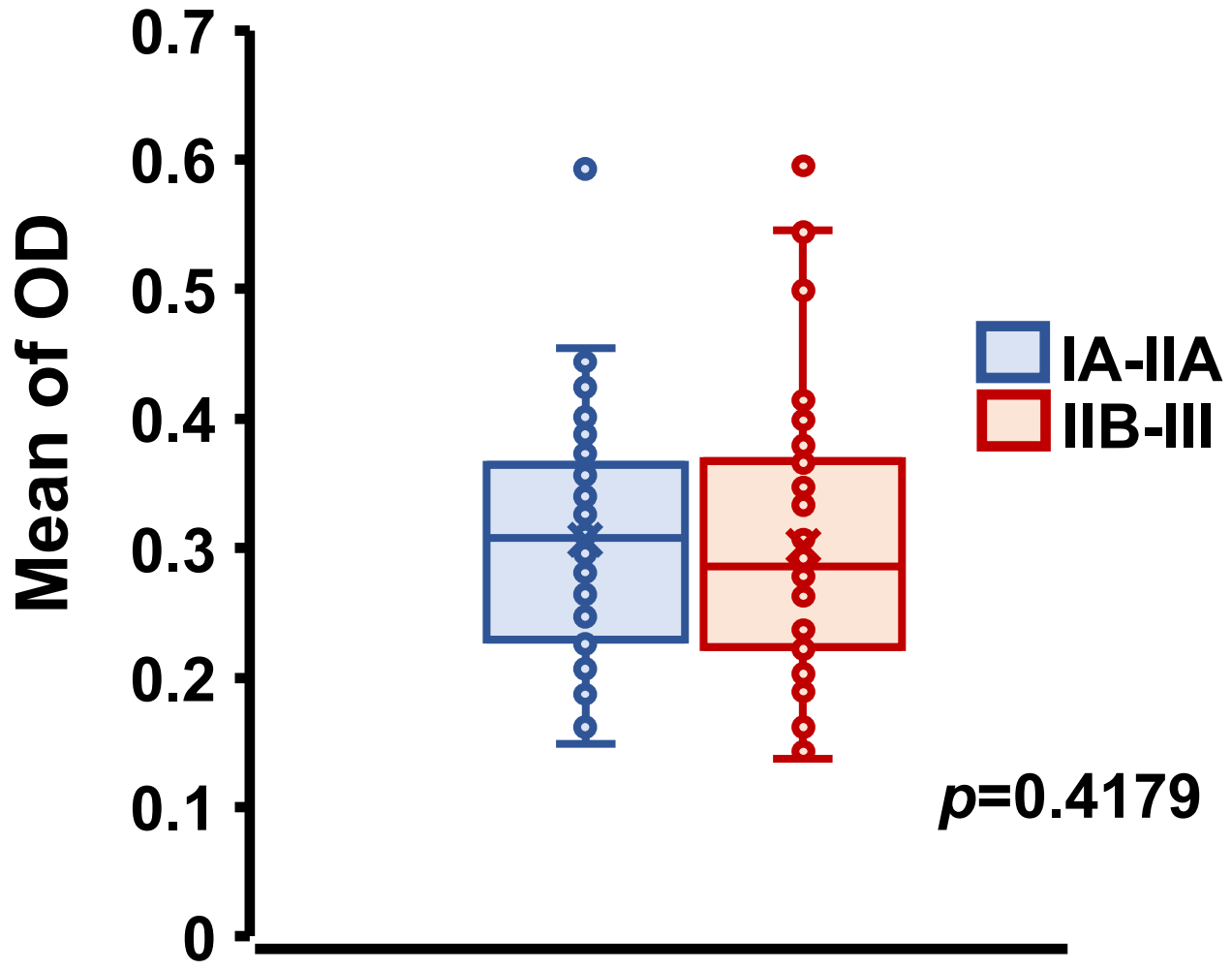

E

Staining Intensity	Tumor Stage			
	IA-IIA	IIB	III	Row totals
Negative/Weak	20	12	8	40
Moderate	21	10	9	40
Strong	23	11	6	40
Column totals	64	33	23	120

Fisher's Exact Test *P*=0.9083

Supplementary Figure S6. ENO2 expression associates with advanced tumor grade in TNBC. A. Immunohistochemical analysis of ENO2 expression in the TNBC tissue array BR1301a. DAB staining intensity was quantified using ImageJ (Fiji), and cases were classified as weak, moderate, or strong staining according to mean optical density (OD) values. **B.** Comparison of mean OD values between low-grade tumors (grades 1–2; n = 43) and high-grade tumors (grade 3; n = 77). Statistical significance was determined by Mann–Whitney U test. **C.** Association between ENO2 staining intensity and tumor grade was evaluated using the Chi-square test. **D.** Comparison of mean OD values between early-stage tumors (stages IA–IIA; n = 64) and late-stage tumors (stages IIB–III; n = 56). Statistical significance was determined by Mann–Whitney U test. **E.** Association between ENO2 staining intensity and tumor stage was evaluated using Fisher's exact test.

Supplementary Figure S7

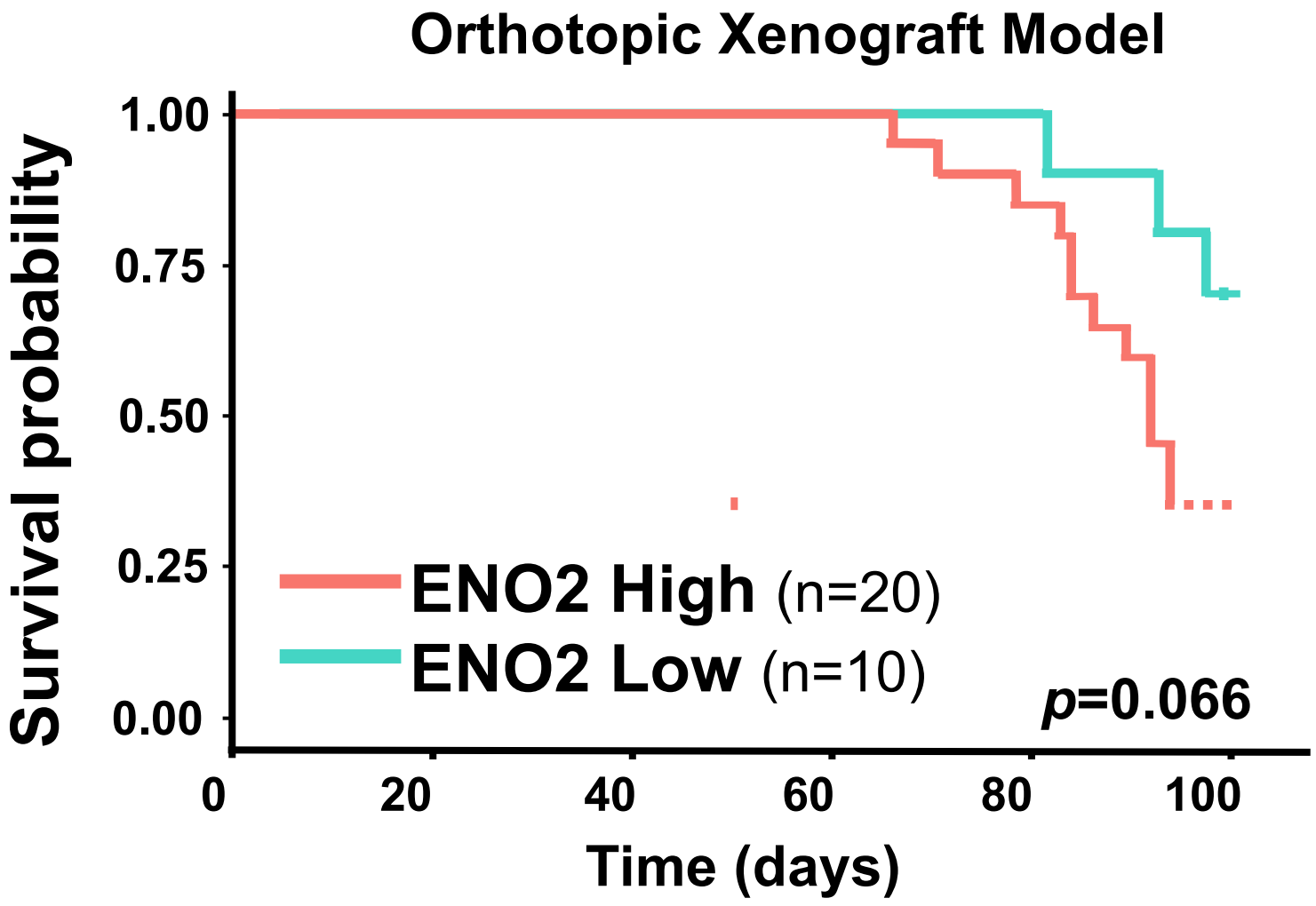

Supplementary Figure S7. ENO2 predicts poor survival in an orthotopic xenograft murine model. MDA-MB-231 control (Ctrl; n = 20) and ENO2-knockdown cells (shENO2#3; 3 × 10^6; n = 10) were inoculated into the fourth mammary fat pad of female NOD-SCID mice to establish orthotopic xenografts with spontaneous pulmonary metastasis. Survival of tumor-bearing mice was monitored, and Kaplan–Meier survival curves were generated using IBM SPSS Statistics 24. Statistical significance was determined by log-rank test.

Supplementary Figure S8

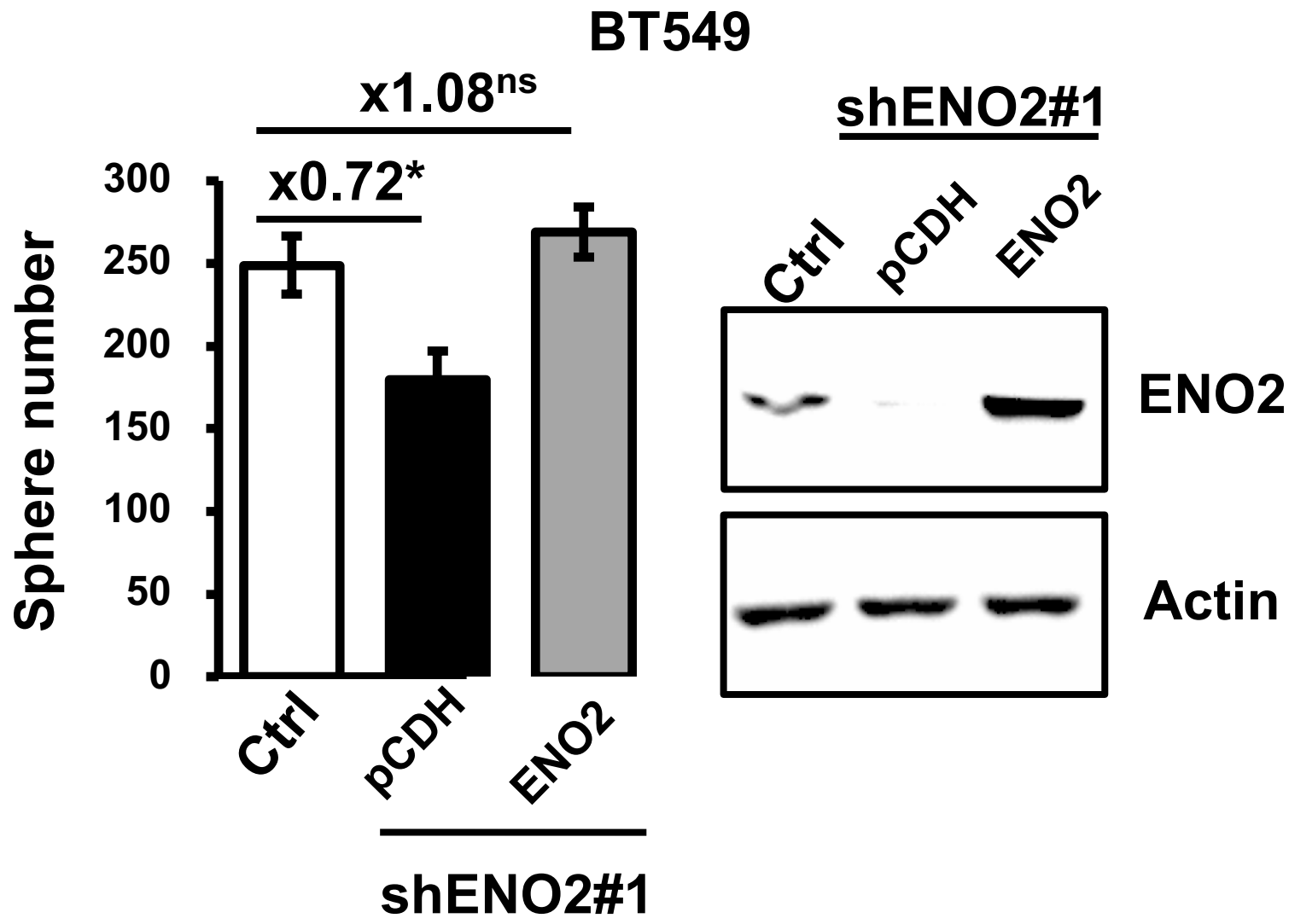

Supplementary Figure S8. Restoration of ENO2 expression rescues the reduced mammosphere-forming ability caused by ENO2 knockdown. BT549 control cells (BT549-pLKO.1+pCDH), ENO2-knockdown cells expressing ENO2 (BT549-shENO2-ENO2), or vector control (BT549-shENO2-pCDH) were analyzed by western blotting (right) and mammosphere formation assays (left). Fold-change values in mammosphere numbers relative to Ctrl are shown above each bar. Data from one representative experiment with three technical replicates (n = 3) are shown as mean ± SD. Statistical analysis was performed using an unpaired t-test: $*p < 0.05$; ns, not significant.

Supplementary Figure S9

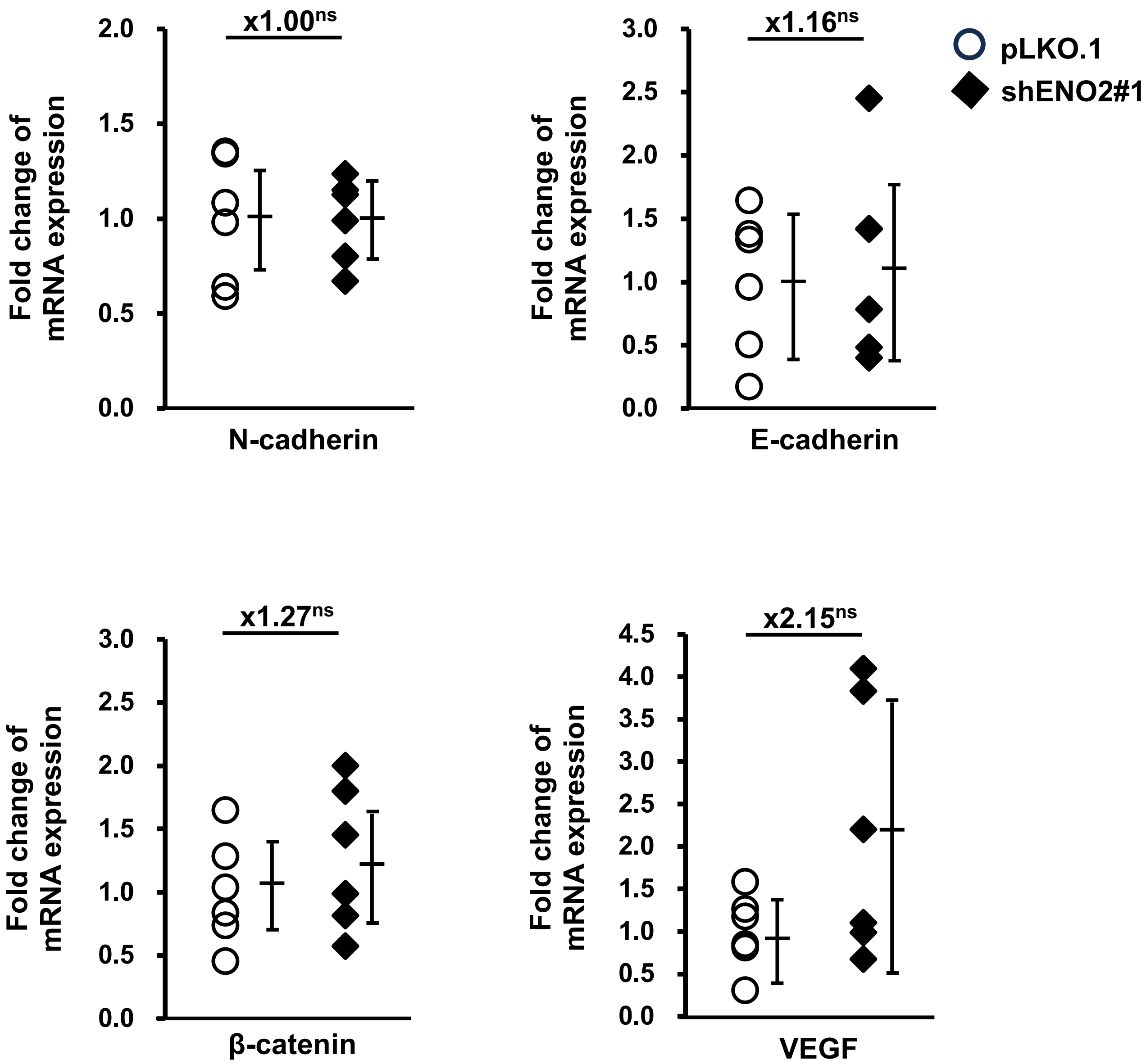

Supplementary Figure S9. ENO2 deficiency does not alter the expression of EMT markers *in vivo.* MDA-MB-231 control (Ctrl; n = 6) and ENO2-knockdown cells (shENO2#1; 3×10^6; n = 6) were orthotopically xenografted into the fourth mammary fat pad of female NOD-SCID mice. qPCR analysis of β-catenin, VEGF, and EMT-related genes (N-cadherin and E-cadherin) were performed on excised xenograft tumors. Data are presented as mean ± SD from the indicated xenograft tumor samples (n=6). Statistical significance was determined by unpaired t-test: ns, not significant.

Supplementary Figure S10

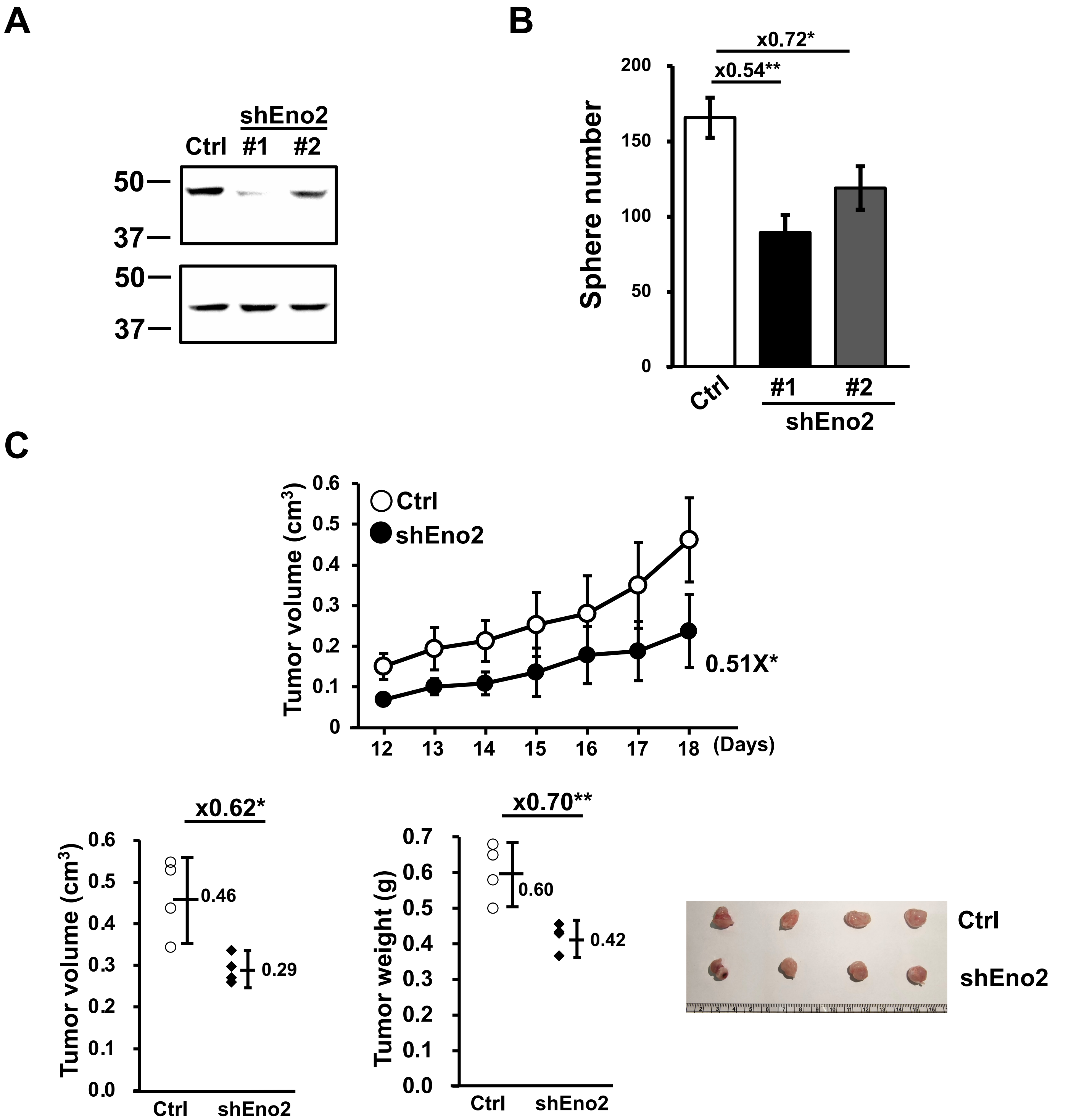

Supplementary Figure S10. Eno2 downregulation suppresses sphere formation and in vivo tumor growth in 4T1 cells. A. Eno2 expression in control and Eno2-KD 4T1 tumor spheres was examined by immunoblotting. **B,** Control and Eno2-KD 4T1 cells were subjected to mammosphere formation assays. Fold-change values in mammosphere number relative to control cells are shown above each bar. Data from one representative experiment with three technical replicates are presented as mean ± SD. Statistical significance was determined using an unpaired Student's *t*-test; *p < 0.05; ns, not significant. **C,** Control and Eno2-KD 4T1 cells (1×10^5 cells per mouse) were inoculated into the fourth mammary fat pad of female BALB/c mice (n = 4 per group), and tumor growth was monitored for 18 days. Tumor sizes were measured daily and plotted as growth curves. At the endpoint, tumors were excised, and tumor volumes were calculated using the formula: $(d_1 \times d_2^2)/2$, where d_1 represents the larger diameter and d_2 represents the smaller diameter. Tumor volumes and tumor weights are presented as dot plots with mean ± SD. Fold-change values and statistical significance are indicated above the comparison lines. Statistical significance was determined using an unpaired Student's *t*-test; **p < 0.01; ***p < 0.001.

Supplementary Figure S11

A

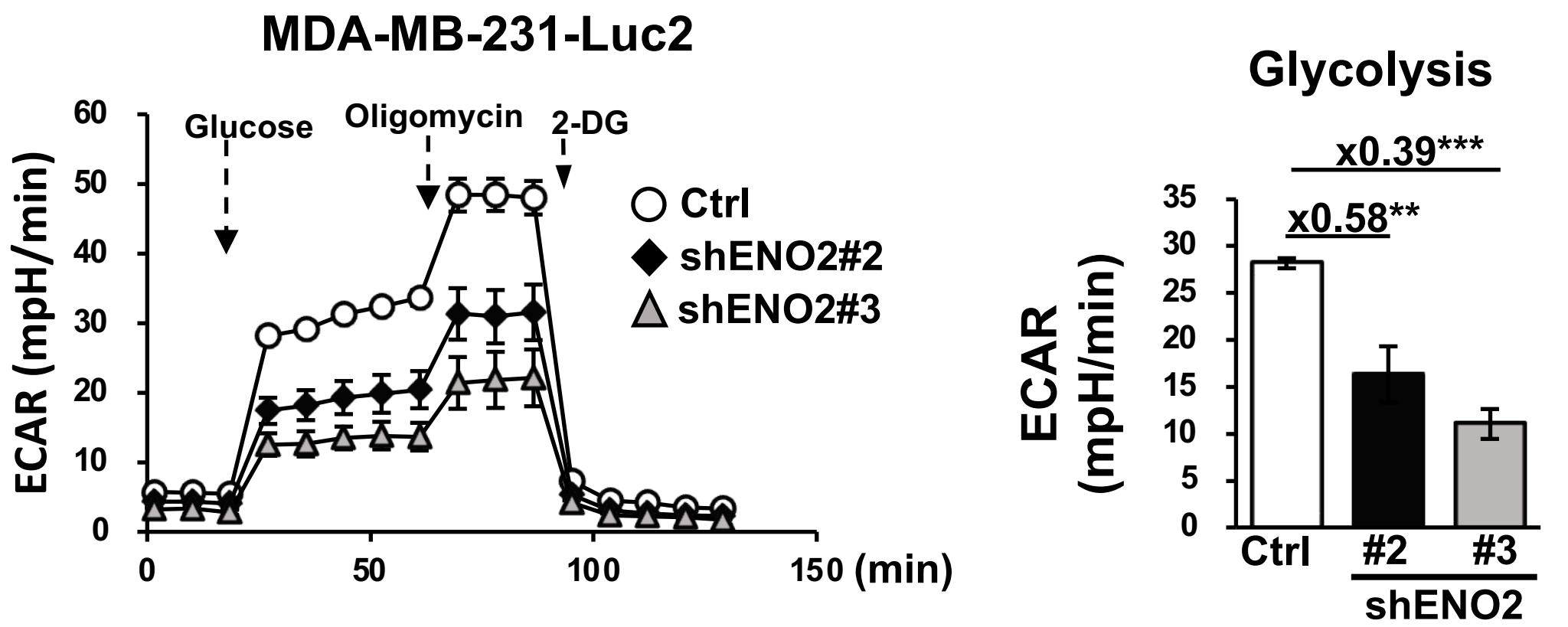

B

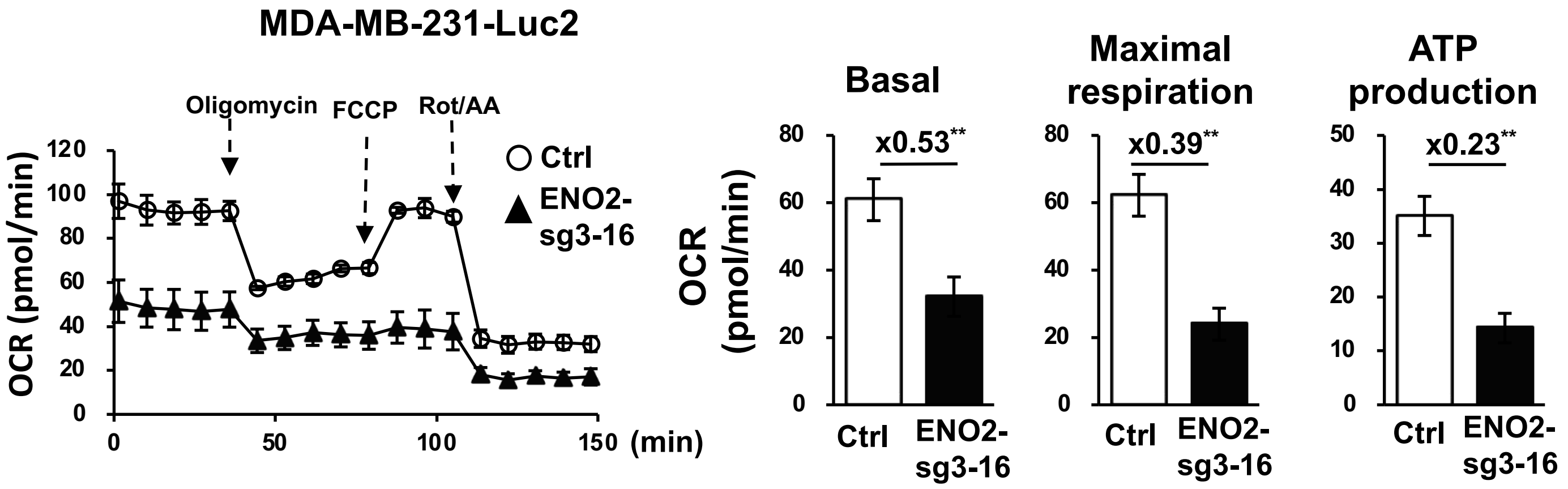

Supplementary Figure S11. ENO2 depletion reduces aerobic glycolysis and OXPHOS activity. **A.** Glycolytic activity was measured in ENO2-knockdown cells using the Seahorse glycolytic stress test. **B.** Mitochondrial respiration was assessed in ENO2-knockout cells using the Seahorse mito-stress test. Data from one representative experiment with three technical replicates (n = 3) are shown as mean ± SD. Statistical significance was determined by unpaired t-test: $^{**}p < 0.01$; $^{***}p < 0.001$.

Supplementary Figure S12

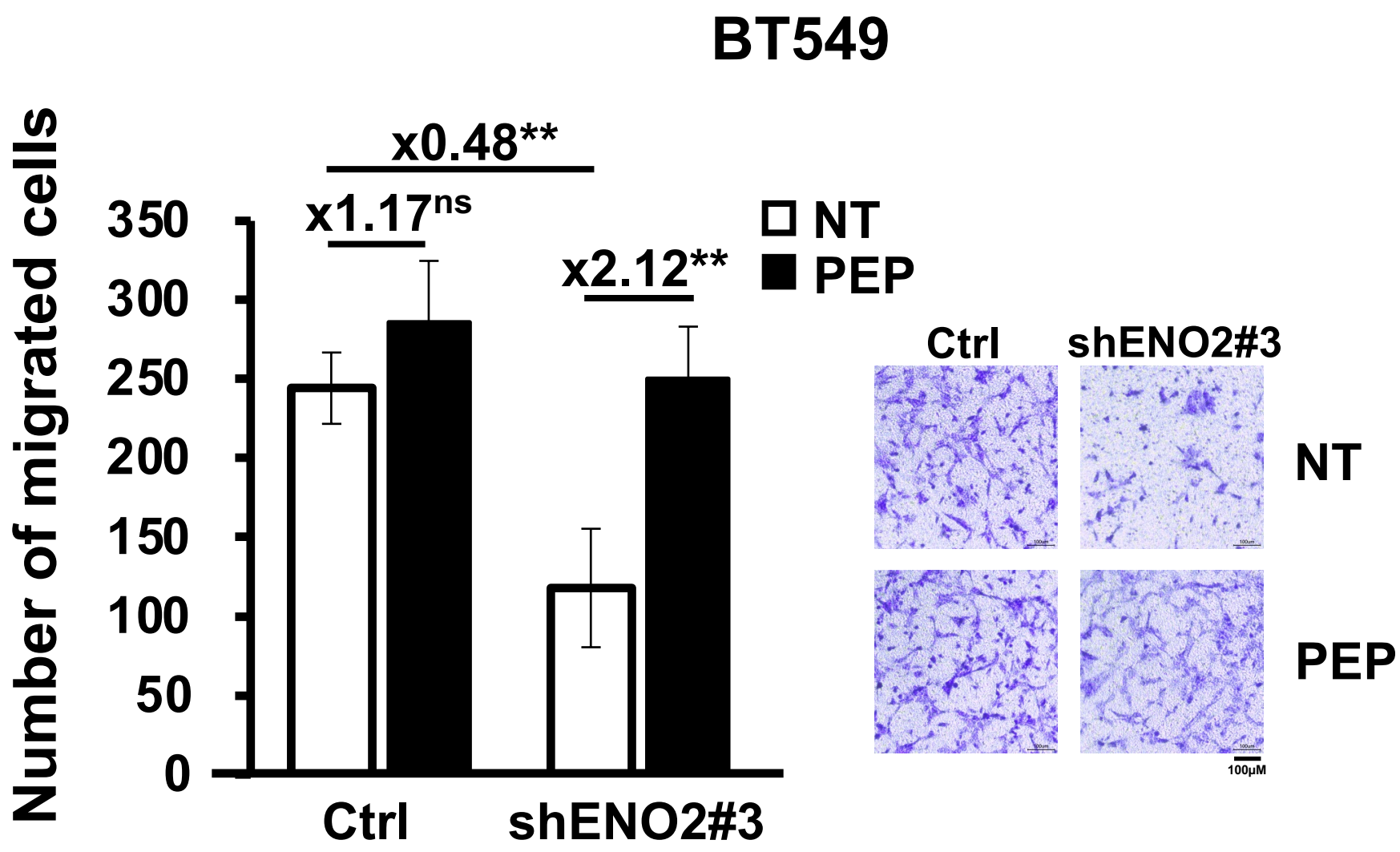

Supplementary Figure S12. PEP treatment rescues the reduced migratory capacity induced by ENO2 deficiency. Control (Ctrl) and ENO2-knockdown BT549 cells (shENO2#3) were treated with or without phosphoenolpyruvate (PEP, 1 mM) and subjected to a transwell migration assay. Migrated cells were quantified and presented as mean ± SD (n = 3 technical repeats). Fold-change values relative to untreated control (NT) are indicated above each bar. Statistical significance was determined using an unpaired t-test: ns, not significant; $*p < 0.05$, $**p < 0.01$. Representative images of migrated cells are shown on the right.

Supplementary Figure S13

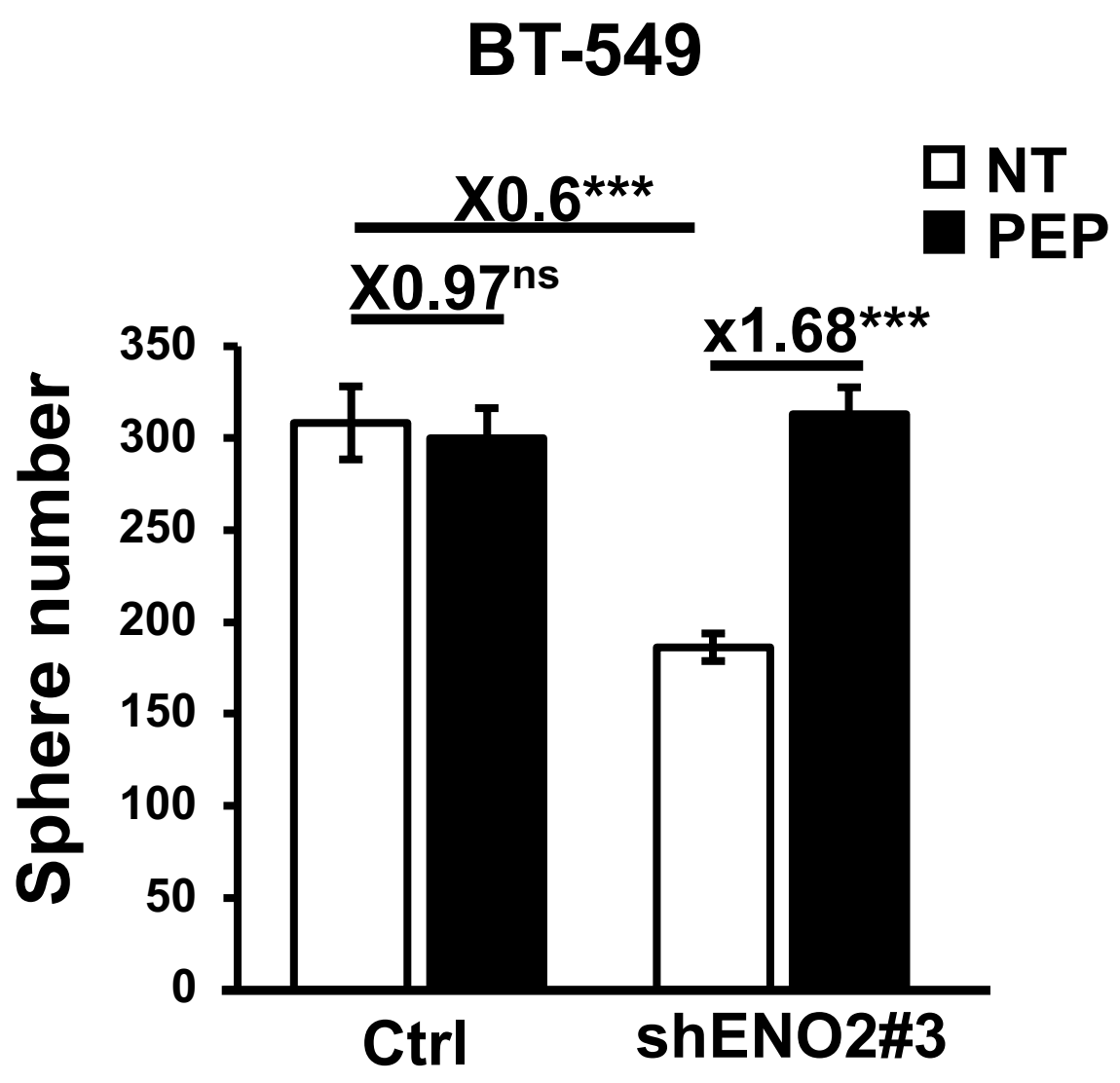

Supplementary Figure S13. Supplementation with PEP rescued the diminished cancer stemness induced by ENO2 deficiency. Control (Ctrl) and ENO2-knockdown BT-549 cells (shENO2#3) were treated with or without PEP (5 mM) respectively for 7 days and subjected to mammosphere assay. Fold-change values in mammosphere numbers relative to Ctrl are shown above each bar. Data from one representative experiment with three technical replicates (n = 3) are shown as mean ± SD. Statistical analysis was performed using an unpaired t-test: ****p* < 0.001; ns, not significant.

Supplementary Figure S14

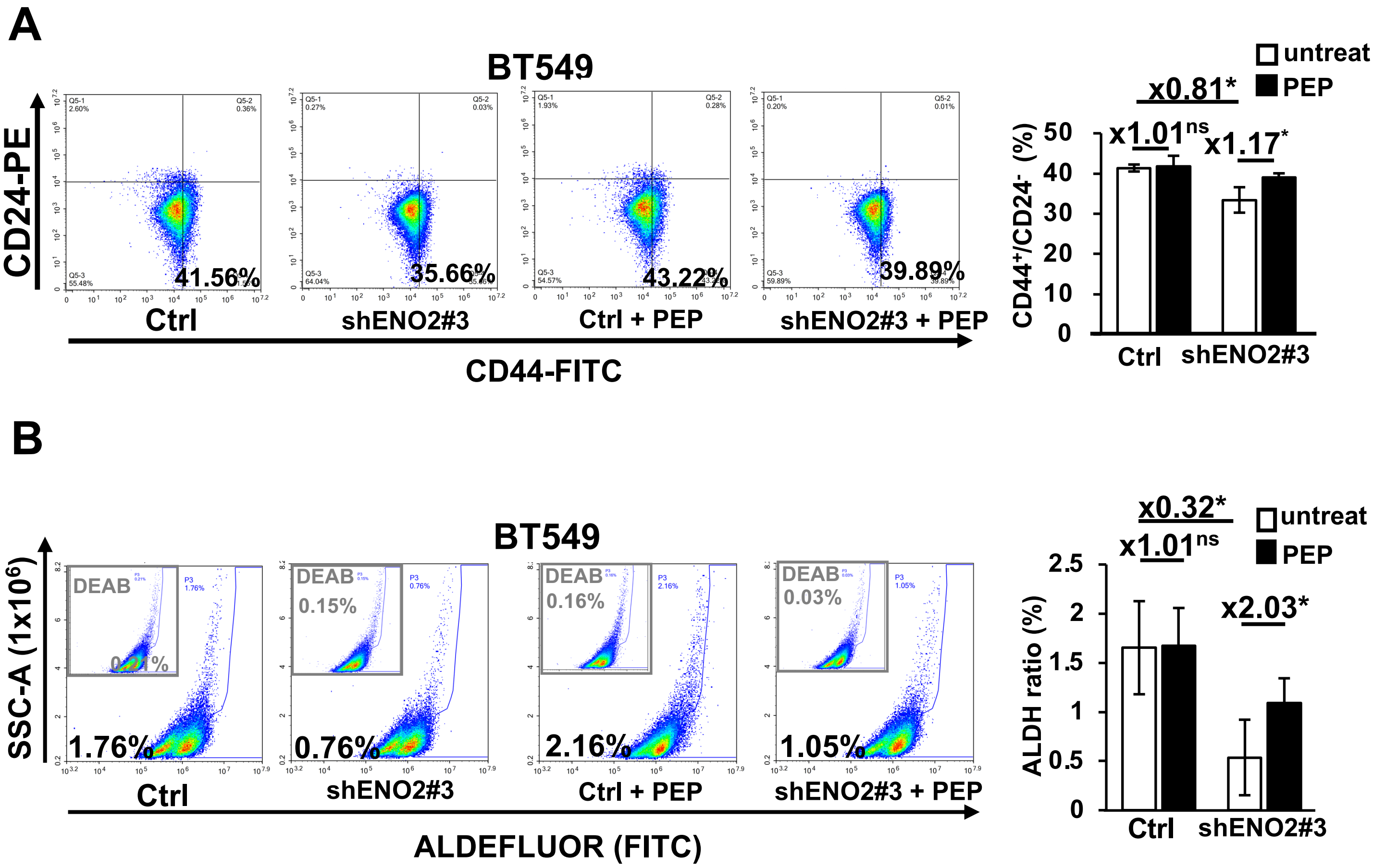

Supplementary Figure S14. PEP supplementation restores CSC-enriched populations in ENO2-knockdown BT-549 cells. A. Representative FACS plots (left) and quantification (right) of $CD44^{+}/CD24^{-}$ populations in control and ENO2-knockdown cells treated with or without PEP. **B.** Representative FACS plots (left) and quantification (right) of $ALDH^{+}$ populations under the same conditions. Data represent mean ± SD from three independent experiments, with fold-change values indicated above bars. Statistical significance was determined by unpaired t-tests: ns, not significant; $*p < 0.05$.

Supplementary Figure S15

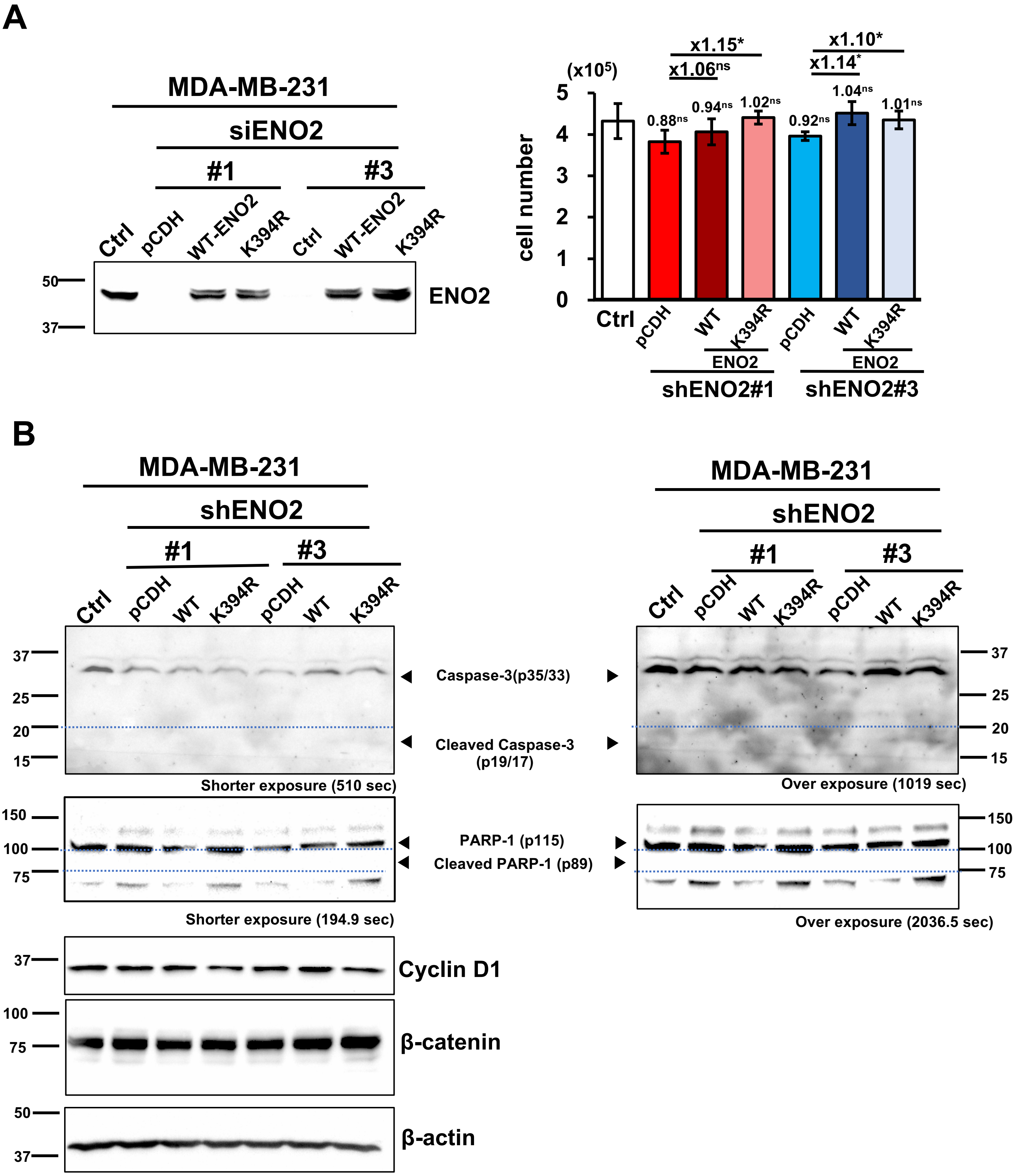

Supplementary Figure S15. ENO2 deficiency does not affect cell proliferation or induce detectable apoptotic marker cleavage. A. ENO2 expression in ENO2-knockdown (ENO2-KD) cells and ENO2-KD cells reconstituted with wild-type ENO2 or the catalytically impaired K394R mutant was examined by immunoblotting. Cell proliferation was assessed by direct cell counting over three consecutive days using flow cytometry. Data from one representative experiment with three technical replicates (n = 3) are shown as mean ± SD. Experiments were independently repeated at least three times with consistent results. Statistical significance was determined using an unpaired two-tailed Student's *t*-test; ns, not significant. **B.** β-catenin, Cyclin D1, cleaved PARP-1, and cleaved caspase-3 expression was analyzed by immunoblotting in ENO2-KD cells and ENO2-KD cells reconstituted with wild-type ENO2 or the K394R mutant. Representative immunoblots from at least two independent experiments with similar results are shown.

Supplementary Figure S16

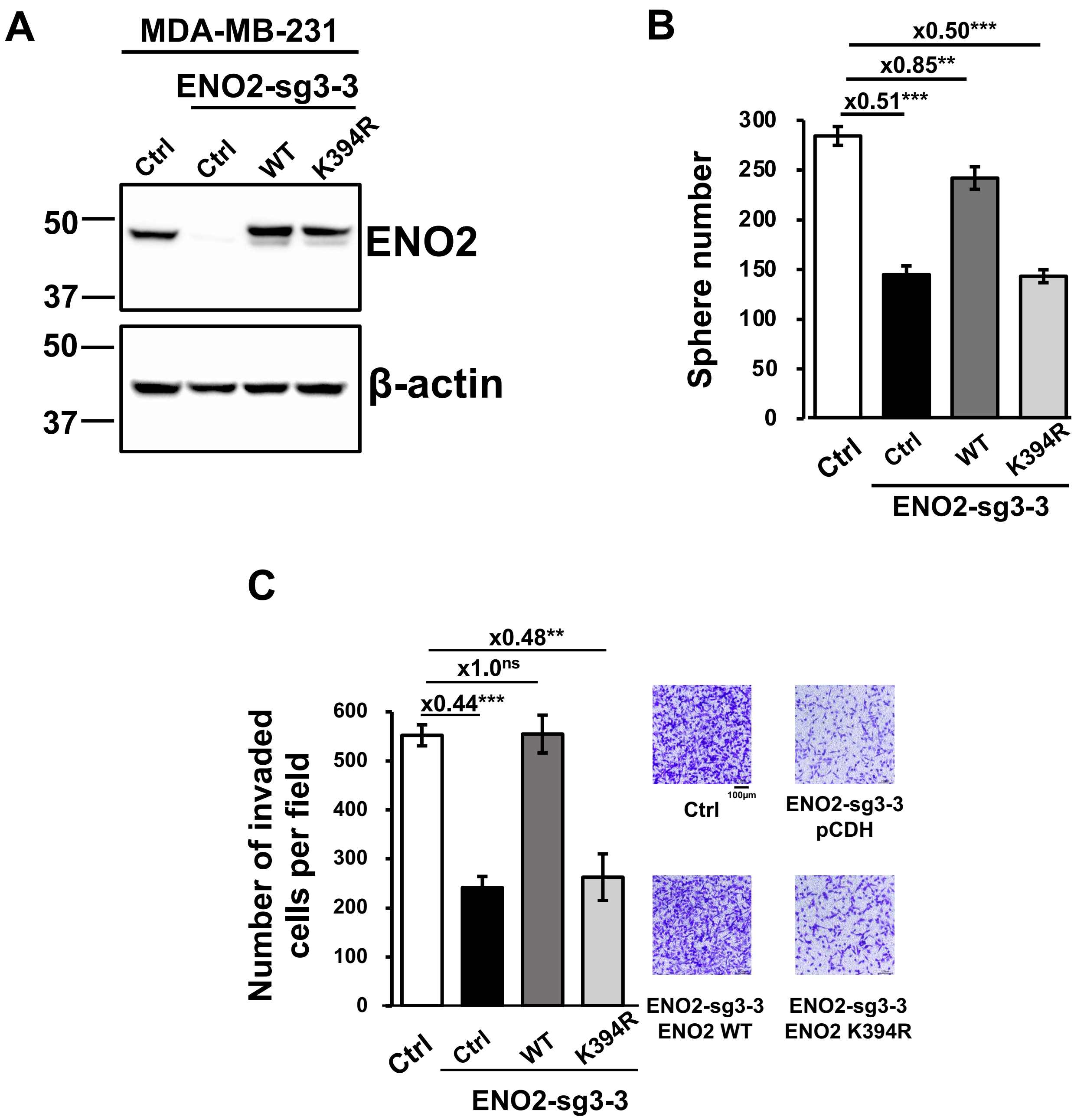

Supplementary Figure S16. Reintroduction of wild-type ENO2, but not the K394R mutant restores the reduced cancer stemness and invasive capacity of MDA-MB-231 ENO2 knockout cells. A. MDA-MB-231 control cells (MDA-MB-231 pLKO.1-pCDH), ENO2-knockout cells reconstituted with wild-type ENO2 or the K394R mutant (MDA-MB-231 ENO2-sg3-3-ENO2 WT, MDA-MB-231 ENO2-sg3-3-ENO2 K394R), and vector control cells (MDA-MB-231 ENO2 sg3-3-pCDH) were analyzed by Western blotting. **B-C.** These cells were subjected to (**B**) sphere formation assay (**C**) transwell invasion assay. Mammosphere numbers and invaded cells were quantified and are presented as mean ± SD (n = 3 technical repeats). Fold-change values relative to control (pLKO.1-pCDH) are indicated above each bar. Statistical significance was determined using an unpaired *t*-test: ns, not significant; **p < 0.01, ***p < 0.001 . Representative images of invaded cells are shown on the right.

Supplementary Figure S17

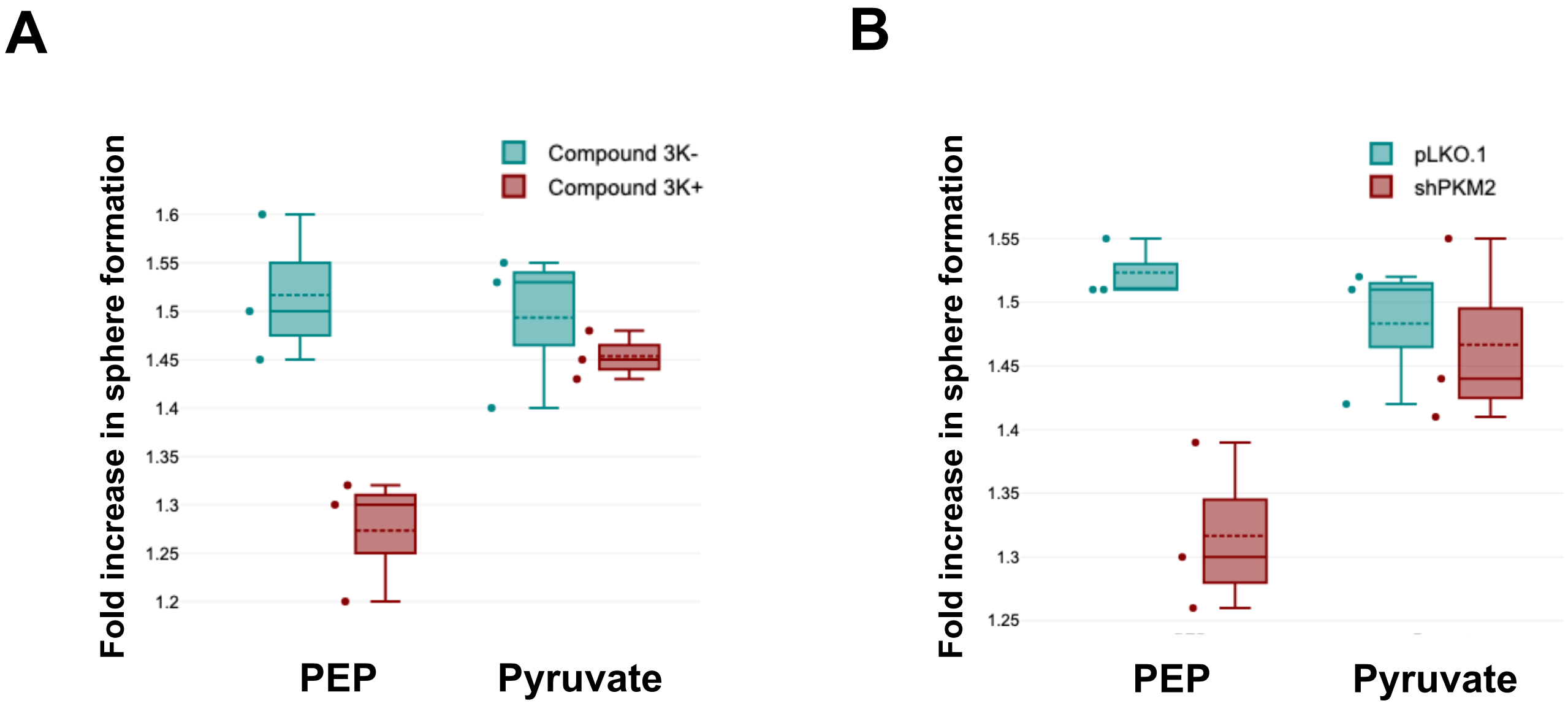

Supplementary Figure S17. PKM2 perturbation preferentially attenuates PEP-mediated rescue of sphere-forming capacity. A. Fold-change analysis of mammosphere-forming efficiency in ENO2-deficient cells treated with PEP or pyruvate in the absence or presence of the PKM2 inhibitor compound 3K. Fold-change values were calculated as the ratio of metabolite-treated to untreated cells within each compound 3K condition. Compound 3K preferentially reduced PEP-mediated rescue, whereas pyruvate-mediated rescue was largely preserved. **B**. Fold-change analysis of mammosphere-forming efficiency in ENO2-deficient cells treated with PEP or pyruvate after control knockdown or PKM2 knockdown. Fold-change values were calculated as the ratio of metabolite-treated to untreated cells within each knockdown condition. Genetic depletion of PKM2 similarly attenuated PEP-mediated rescue, with minimal effect on pyruvate-mediated rescue.

Data are presented as box-and-whisker plots with individual values from independent experiments shown as dots. Boxes indicate the interquartile range, center lines indicate the median, dashed lines indicate the mean, and whiskers indicate the range. Statistical significance was determined by two-way ANOVA to assess the interaction between metabolite treatment and PKM2 perturbation.
