# Supplementary material for: ENO2 sustains cancer stemness and metastatic competence through a phosphoenolpyruvate-dependent metabolic axis in triple-negative breast cancer
Source: Cell Death Dis. 2026 Jul 11;17(1):864. doi: 10.1038/s41419-026-09106-0 (PMC13639003; doi:10.1038/s41419-026-09106-0)
Supplement: Supplementary file 2 [file 41419_2026_9106_MOESM2_ESM.pdf]

**Supplementary Table 1. Characteristics of clinical samples (BRE1201)**

| Characteristics | Case Numbers |
| --- | --- |
| ***TNBC patients*** | **89** |
| **Age** | |
| < 50 | 39 (43.82%) |
| ≥ 50 | 50 (56.18%) |
| **Tumor Grade** | |
| Grade 1 | 3 (3.37%) |
| Grade 2 | 39 (43.82%) |
| Grade 3 | 47 (52.81%) |
| **Tumor Stage** | |
| IA | 7 (7.87%) |
| IIA | 39 (43.82%) |
| IIB | 25 (28.09%) |
| IIIA-C | 18 (30.51%) |
| ***Normal breast*** | **3** |

**Supplementary Table 2. Characteristics of clinical samples (BR1301a)**

| Characteristics | Case Numbers |
|---|---|
| ***TNBC patients*** | **120** |
| **Age** | |
| < 50 | 55 (45.83%) |
| ≥ 50 | 65 (54.17%) |
| **Tumor Grade** | |
| Grade 1 | 2 (3.37%) |
| Grade 2 | 41 (43.82%) |
| Grade 3 | 77 (52.81%) |
| **Tumor Stage** | |
| IA | 6 (7.87%) |
| IIA | 58 (43.82%) |
| IIB | 33 (28.09%) |
| IIIA-C | 23 (30.51%) |
| ***Normal breast*** | **2** |
