# Supplementary material for: ENO2 sustains cancer stemness and metastatic competence through a phosphoenolpyruvate-dependent metabolic axis in triple-negative breast cancer
Source: Cell Death Dis. 2026 Jul 11;17(1):864. doi: 10.1038/s41419-026-09106-0 (PMC13639003; doi:10.1038/s41419-026-09106-0)
Supplement: Supplementary file 3 [file 41419_2026_9106_MOESM3_ESM.pdf]

Uncropped images

Figure. 2A

PAGE 1

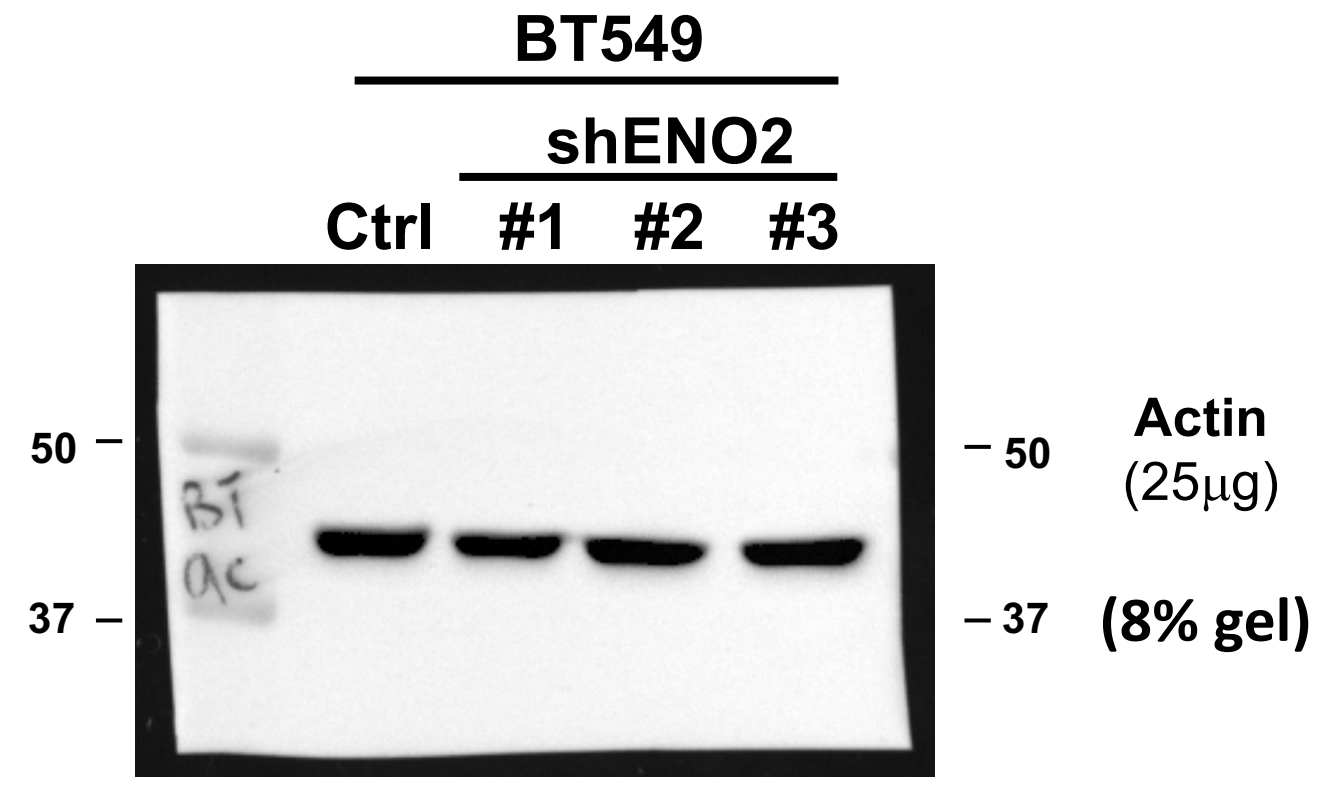

PAGE 2

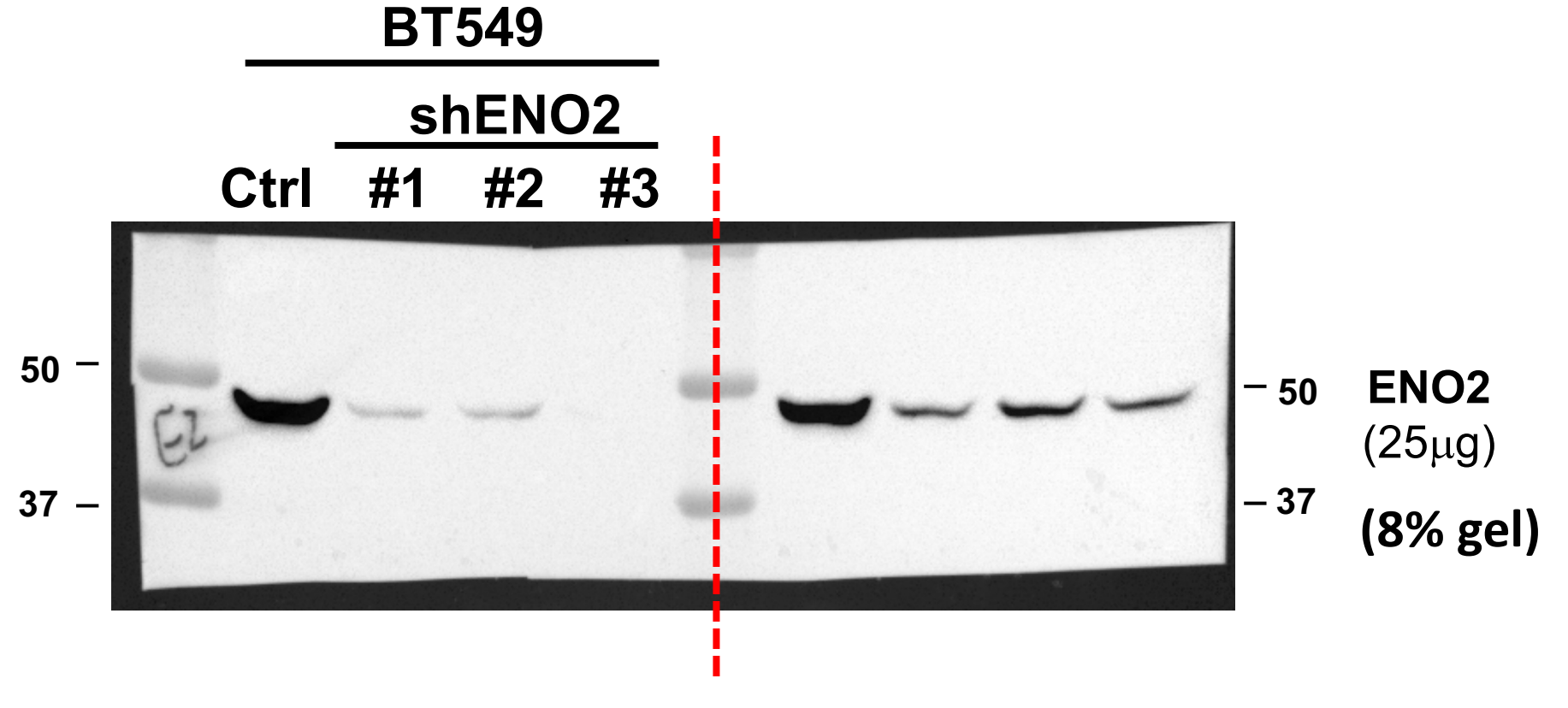

To produce data for Figure 2A, 25 μg samples were loading into PAGE 1 and PAGE 2. PAGE 1 and PAGE 2 were put into the same tank, and all the PAGEs were subjected to electrophoresis simultaneously. After protein transfer, membranes are sliced along the red dashed line and hybridized with indicated antibodies.

Uncropped images

Figure. 2B

PAGE 1

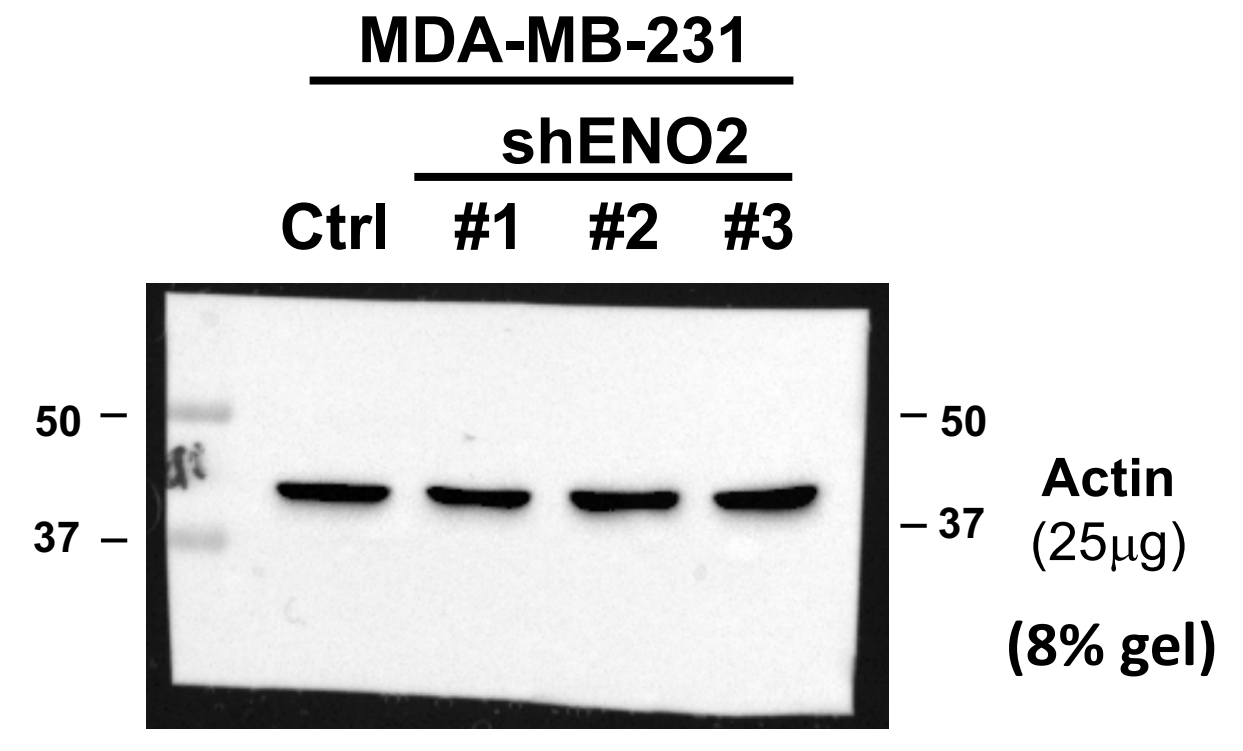

PAGE 2

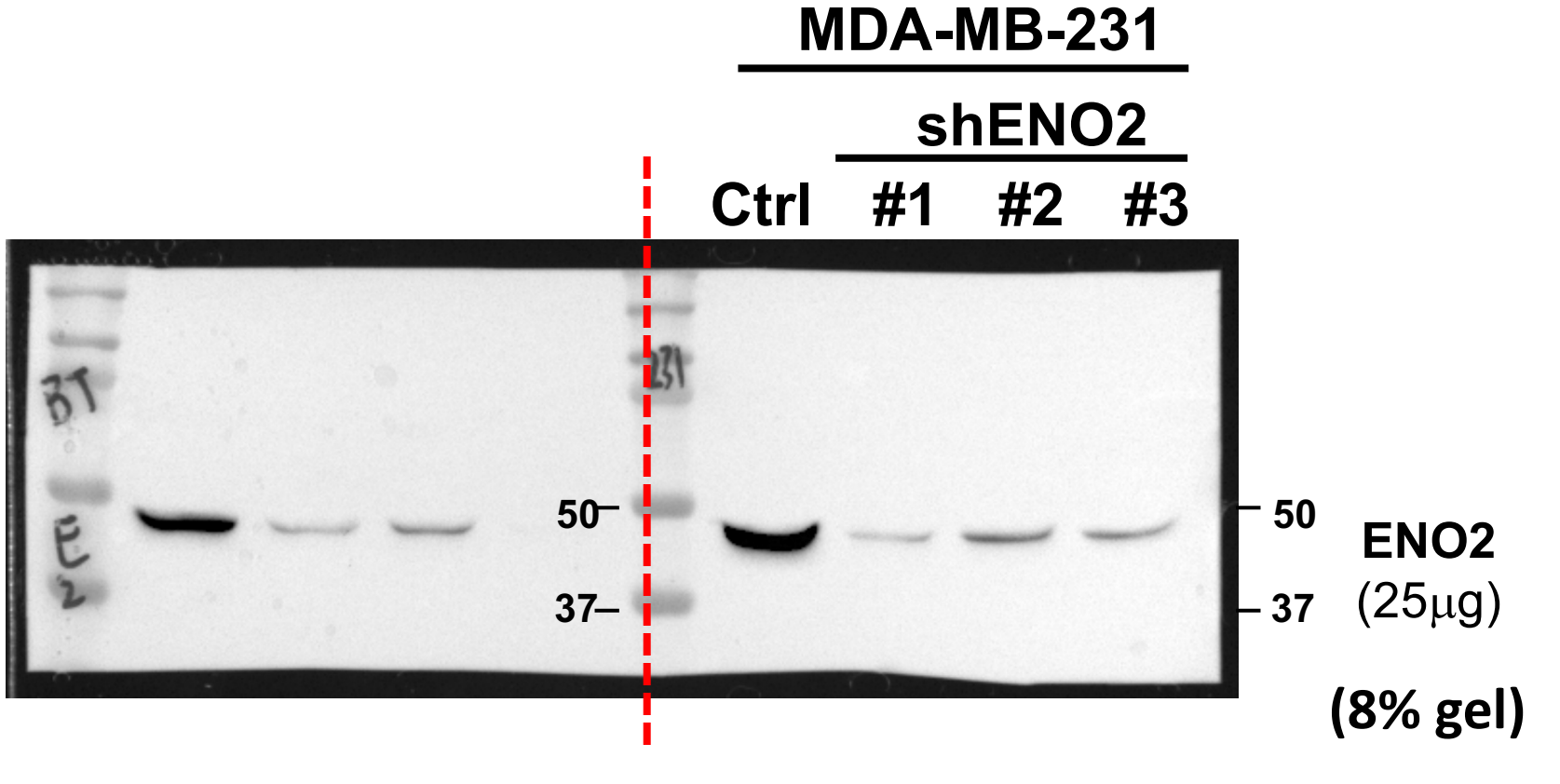

To produce data for Figure 2B, 25 μg samples were loading into PAGE 1 and PAGE 2. PAGE 1 and PAGE 2 were put into the same tank, and all the PAGEs were subjected to electrophoresis simultaneously. After protein transfer, membranes are sliced along the red dashed line and hybridized with indicated antibodies.

Uncropped images

Figure. 2C

PAGE 1

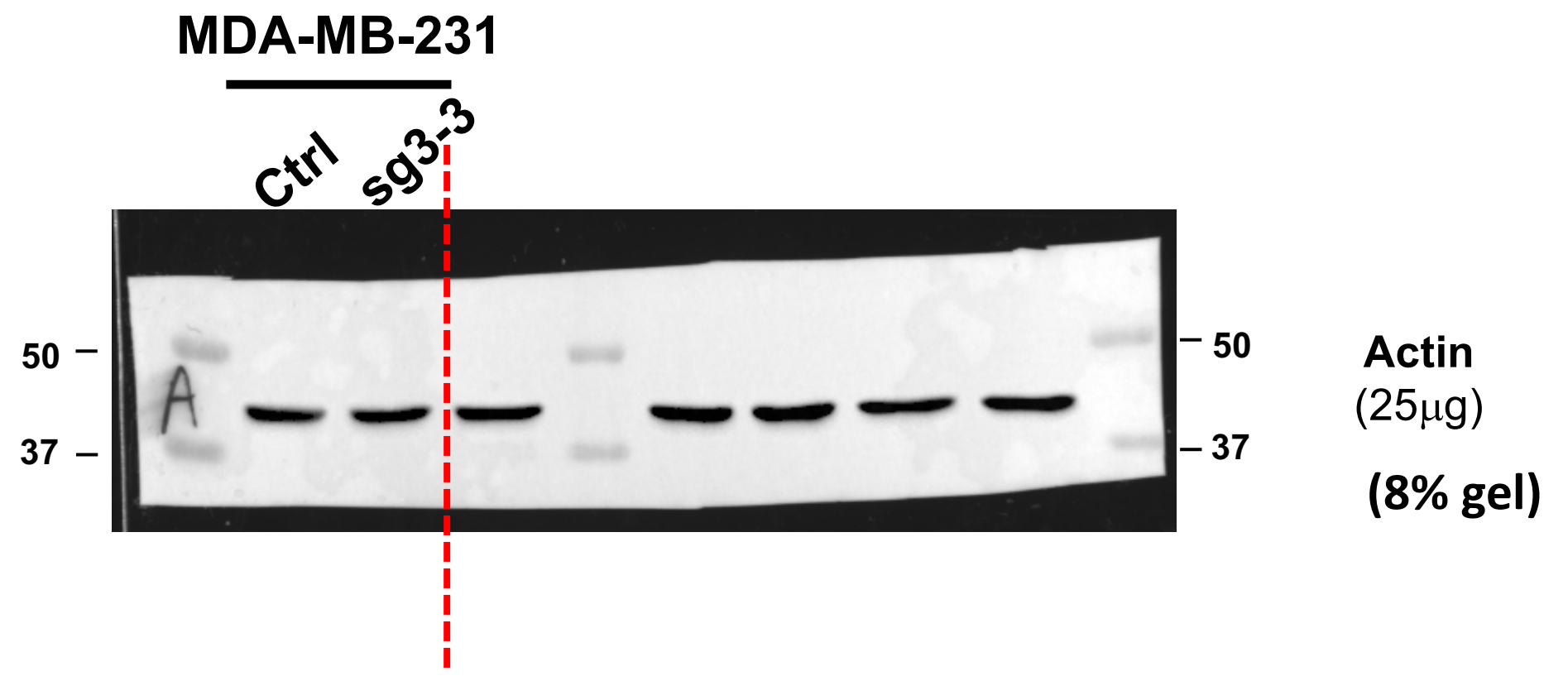

PAGE 2

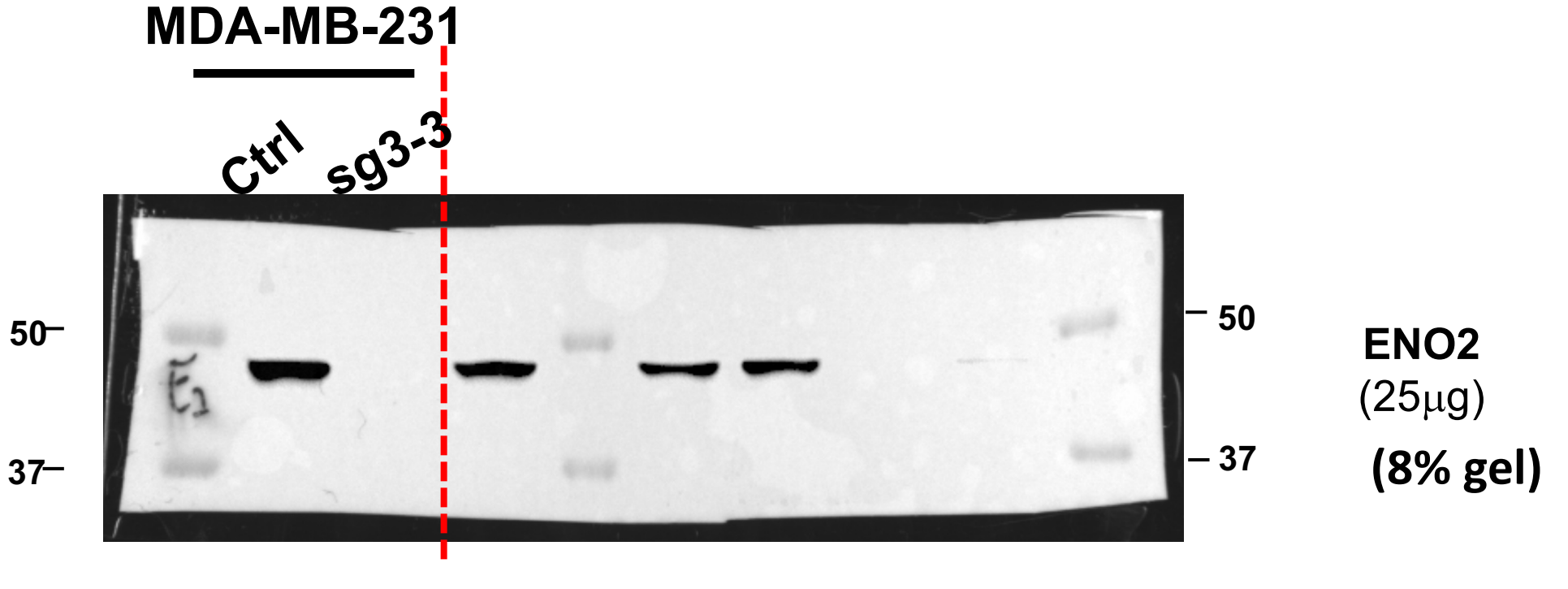

To produce data for Figure 2C, 25 μg samples were loading into PAGE 1 and PAGE 2. PAGE 1 and PAGE 2 were put into the same tank, and all the PAGEs were subjected to electrophoresis simultaneously. After protein transfer, membranes are sliced along the red dashed line and hybridized with indicated antibodies.

Uncropped images

Figure. 2E

PAGE 1

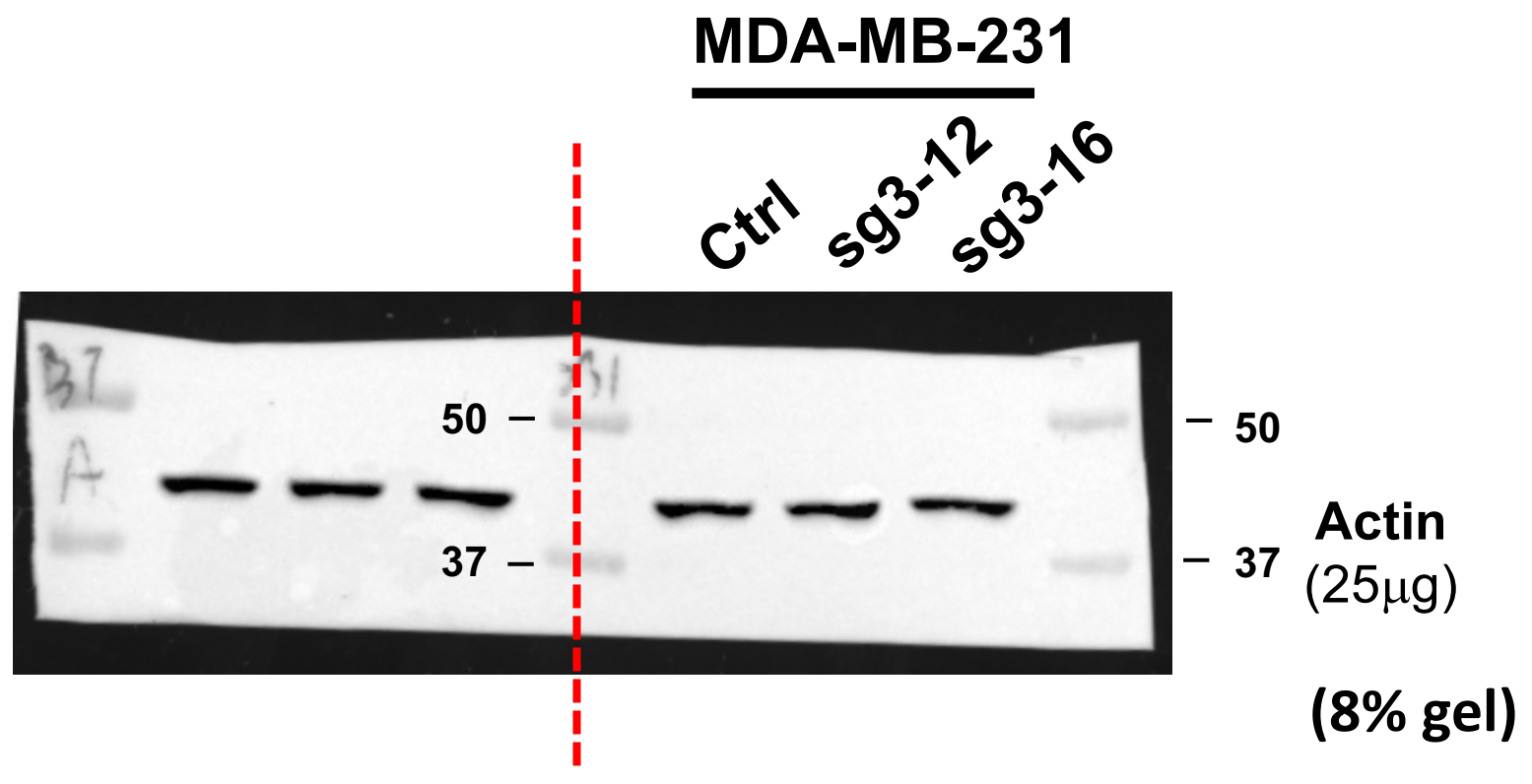

PAGE 2

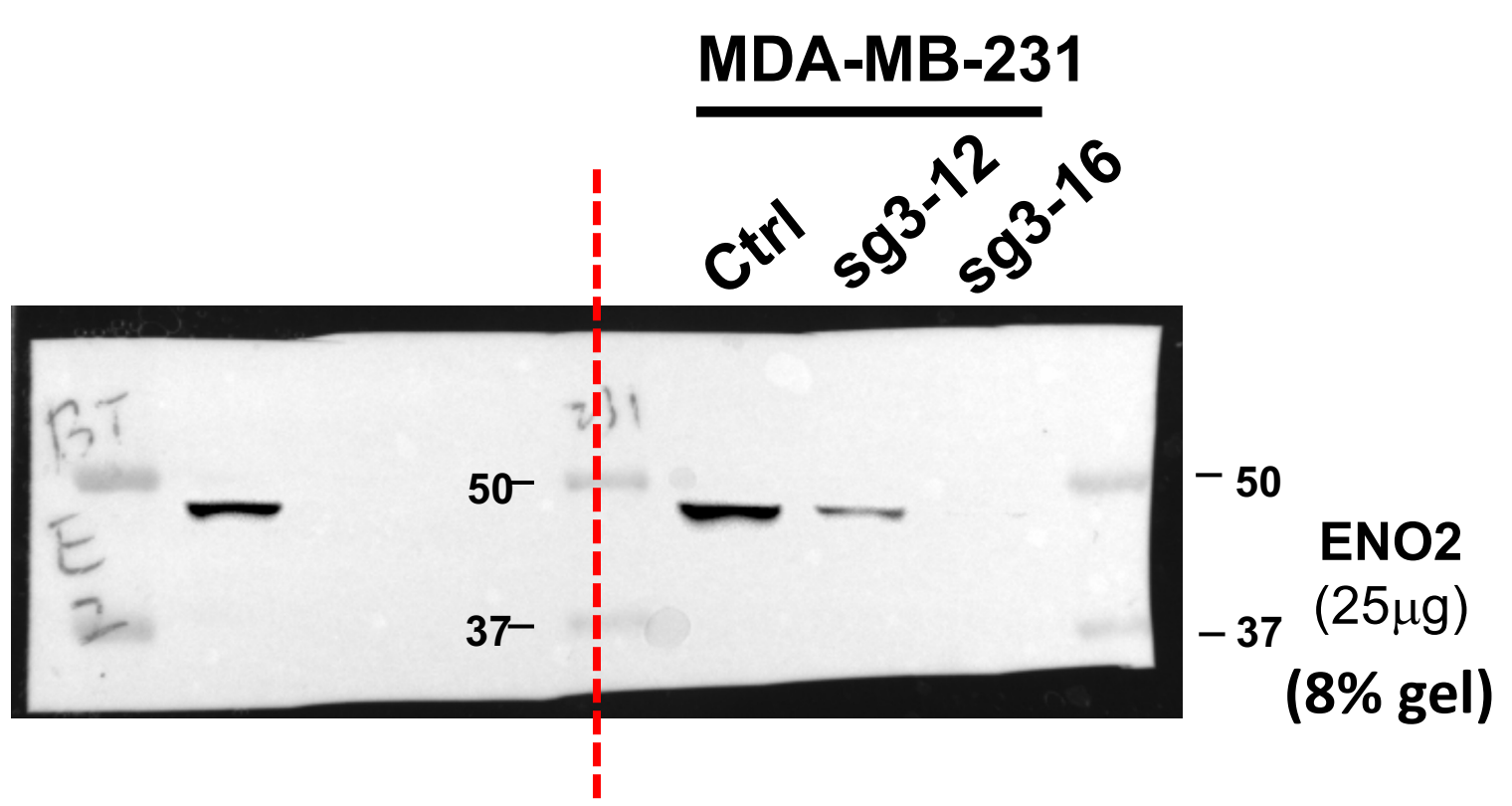

To produce data for Figure 2E, 25 μg samples were loading into PAGE 1 and PAGE 2. PAGE 1 and PAGE 2 were put into the same tank, and all the PAGEs were subjected to electrophoresis simultaneously. After protein transfer, membranes are sliced along the red dashed line and hybridized with indicated antibodies.

Uncropped images

Figure. 3A

PAGE 1

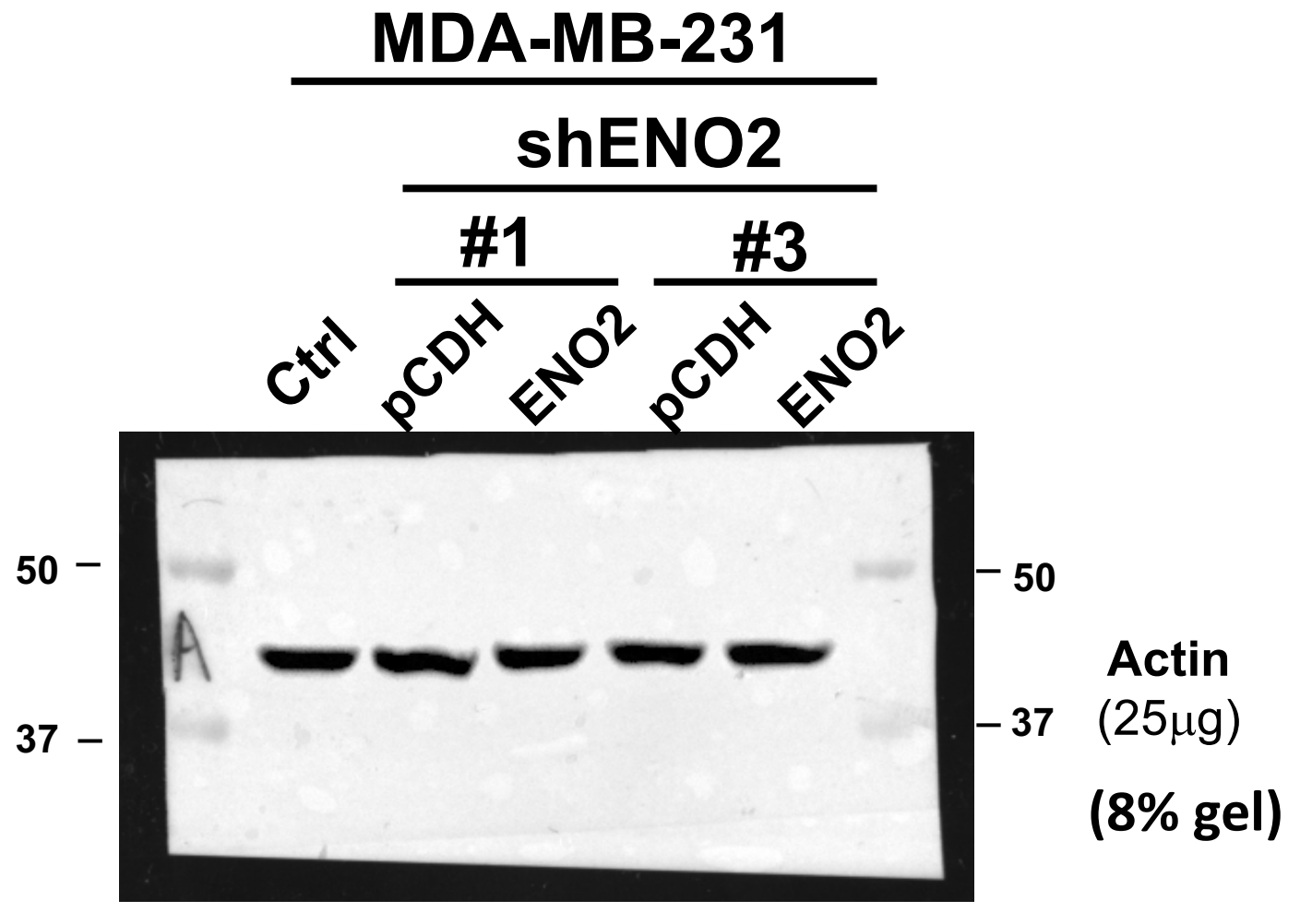

PAGE 2

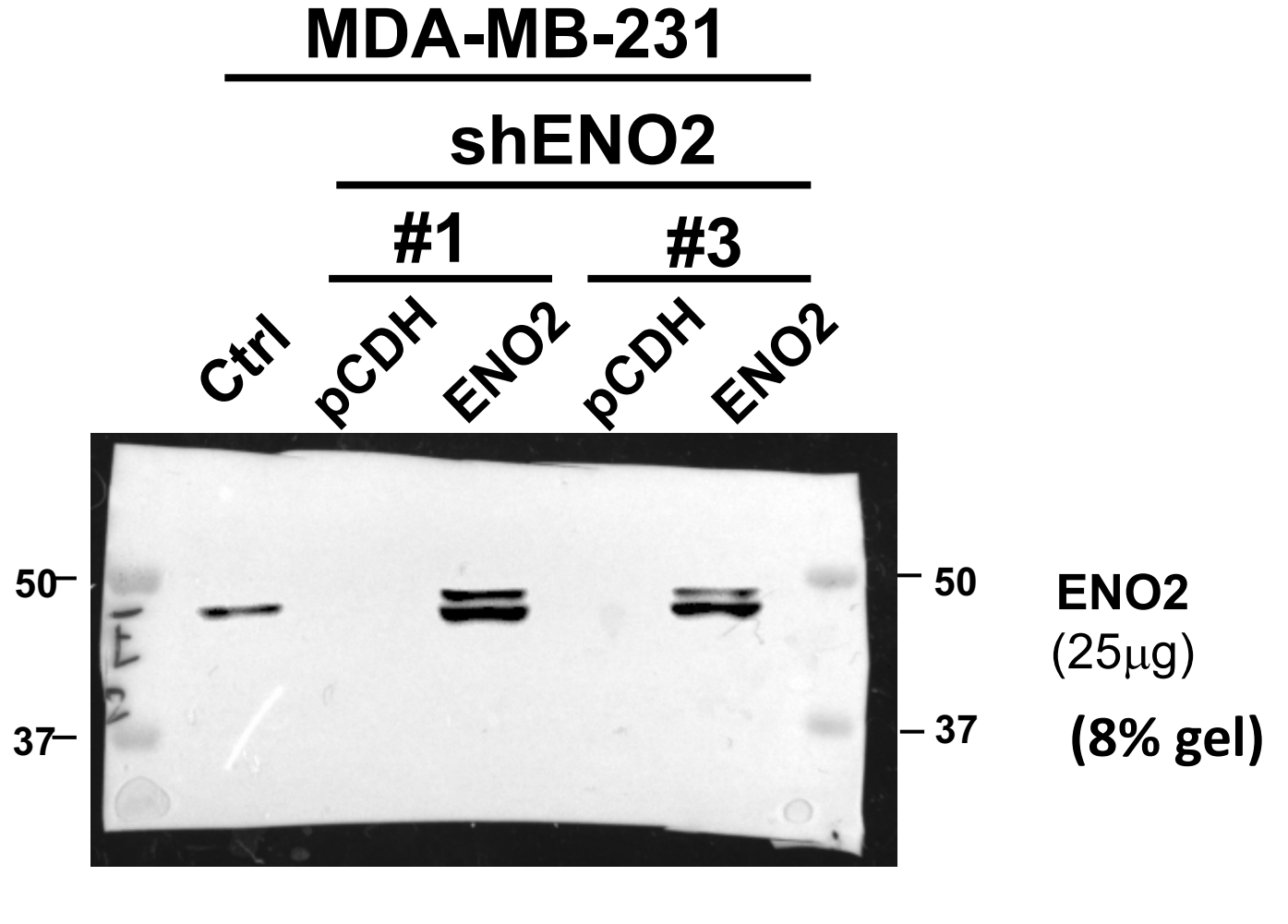

To produce data for Figure 3A, 25 μg samples were loading into PAGE 1 and PAGE 2. PAGE 1 and PAGE 2 were put into the same tank, and all the PAGEs were subjected to electrophoresis simultaneously. After protein transfer, membranes are sliced and hybridized with indicated antibodies.

Uncropped images

Figure. 3H

PAGE 1

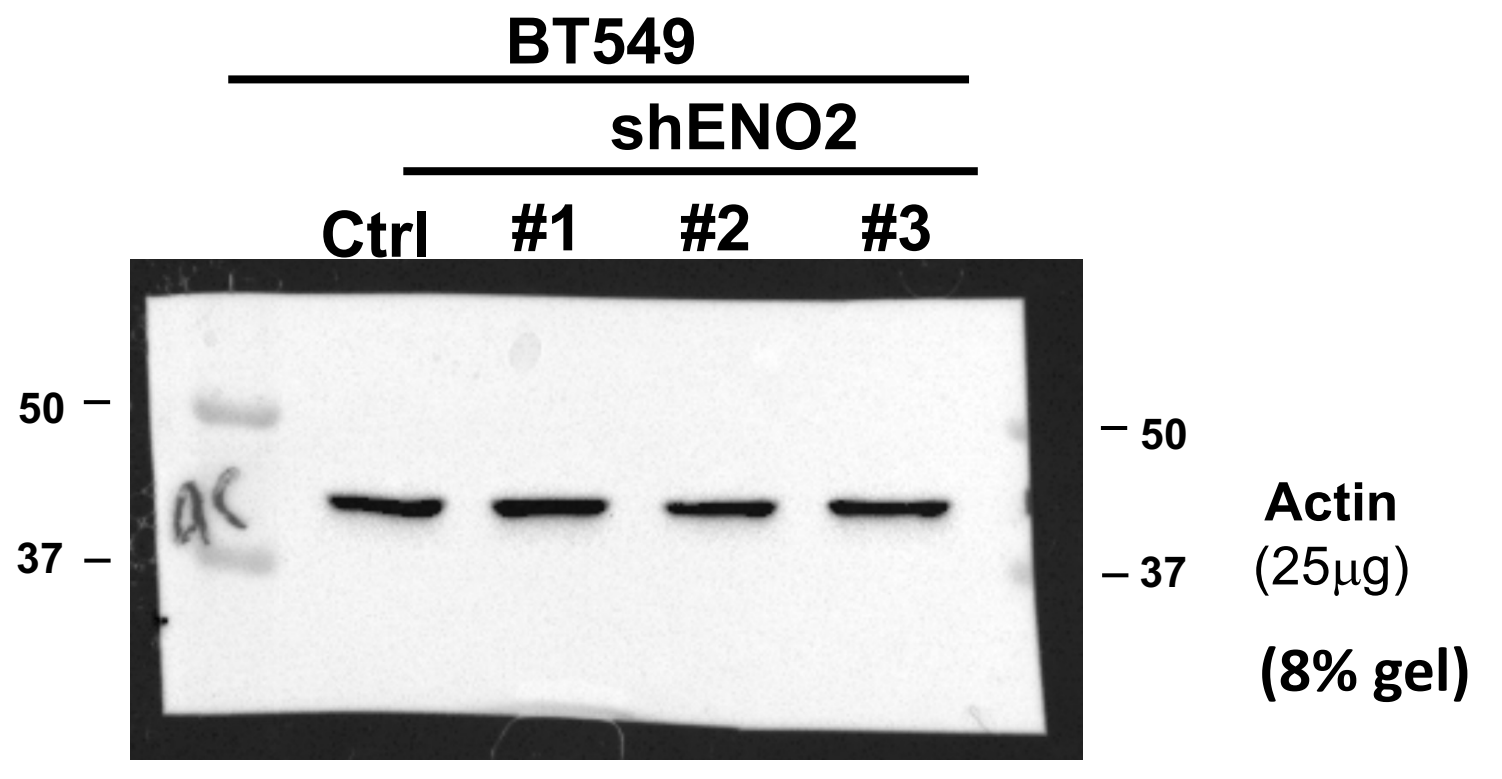

PAGE 2

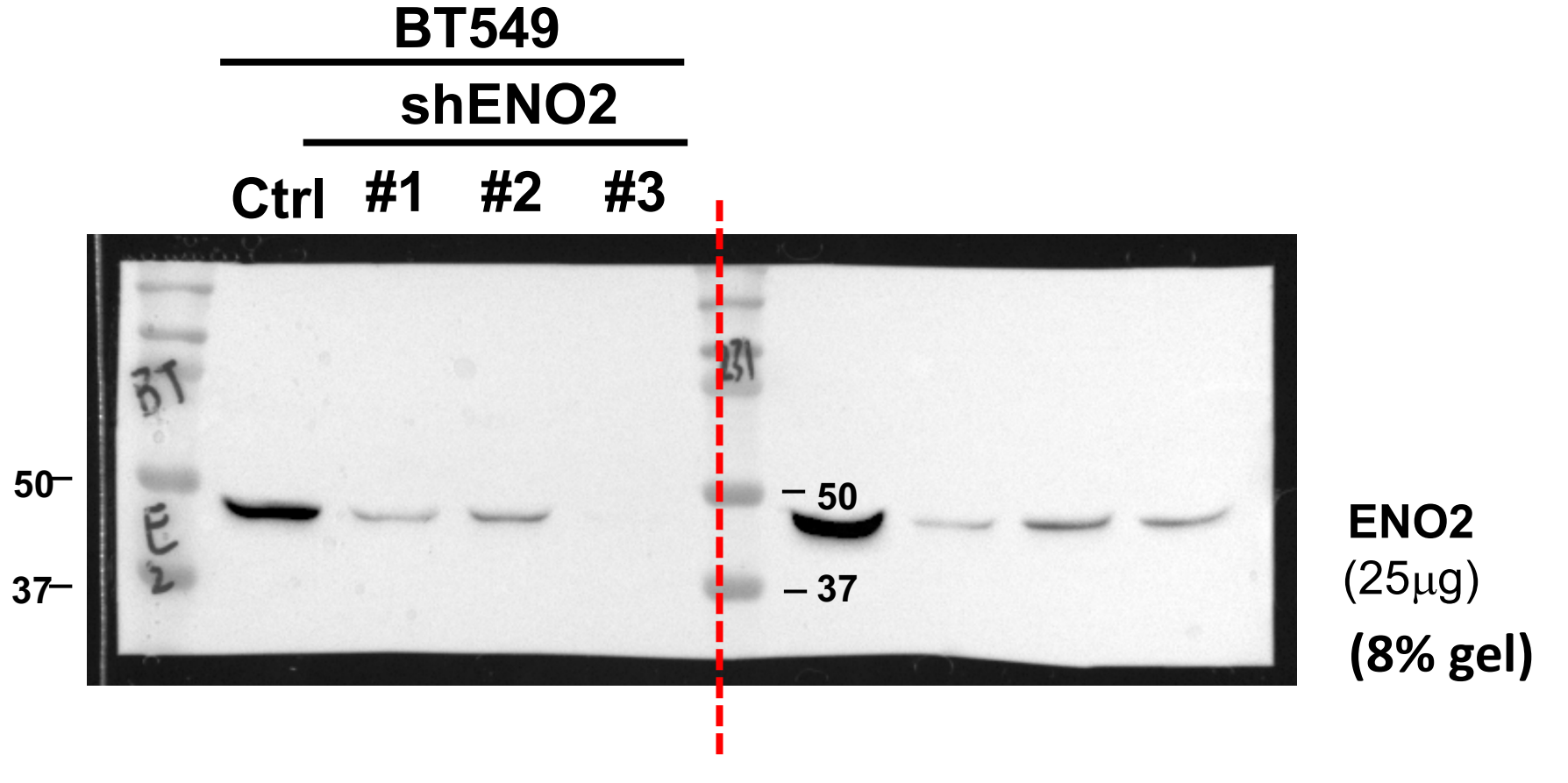

PAGE 3

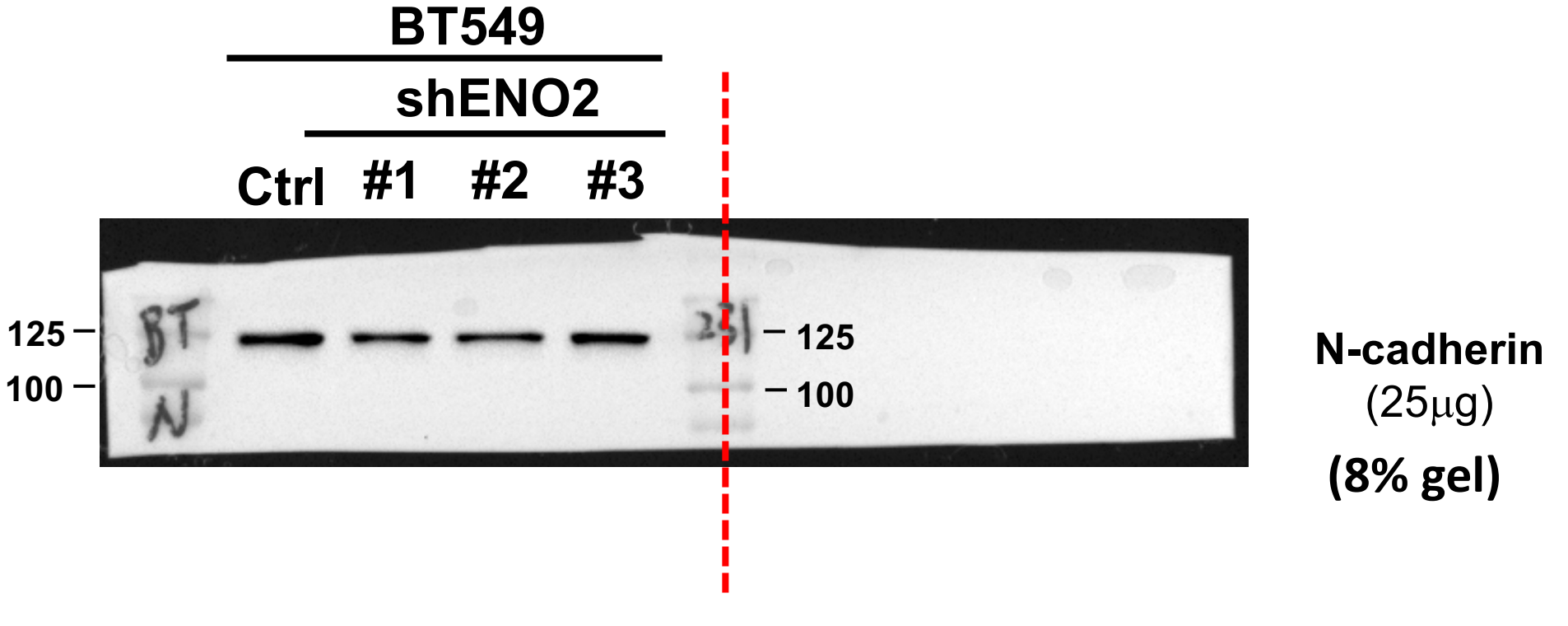

To produce data for Figure 3H, 25 µg samples were loading into PAGE 1 ,PAGE 2 and PAGE 3. PAGE 1, PAGE 2 and PAGE 3 were put into the same tank, and all the PAGEs were subjected to electrophoresis simultaneously. After protein transfer, membranes are sliced along the red dashed line and hybridized with indicated antibodies.

Uncropped images

Figure. 3H

PAGE 1

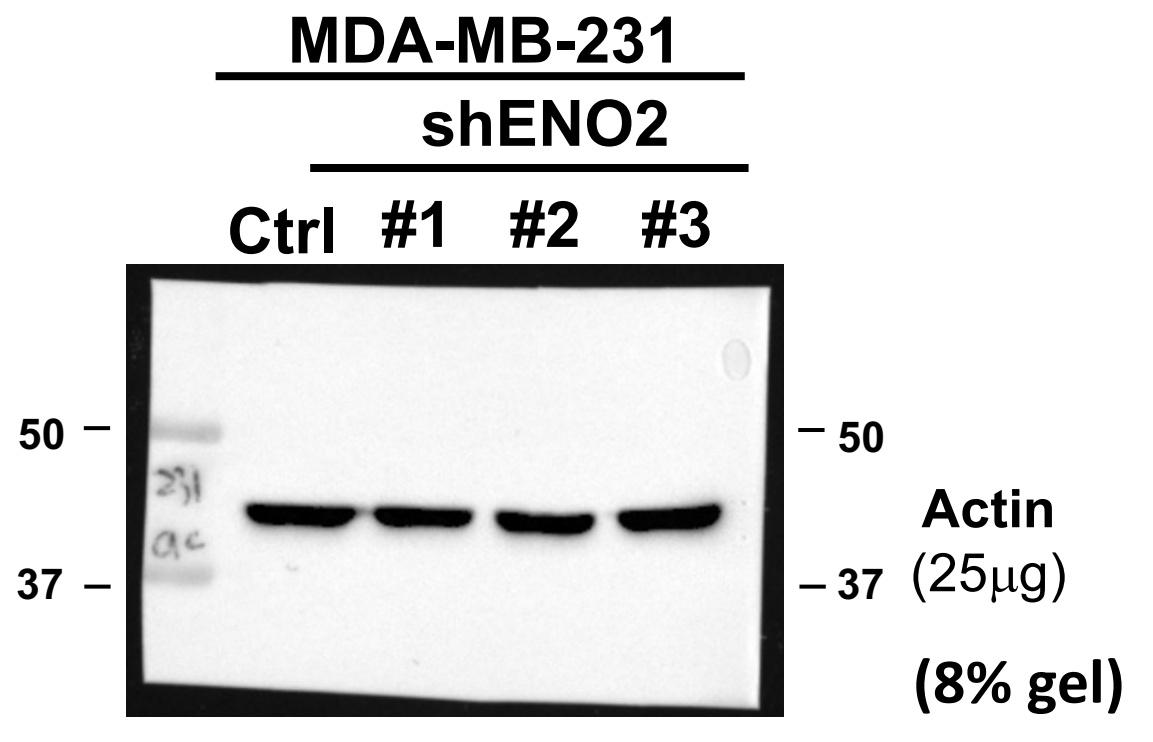

PAGE 2

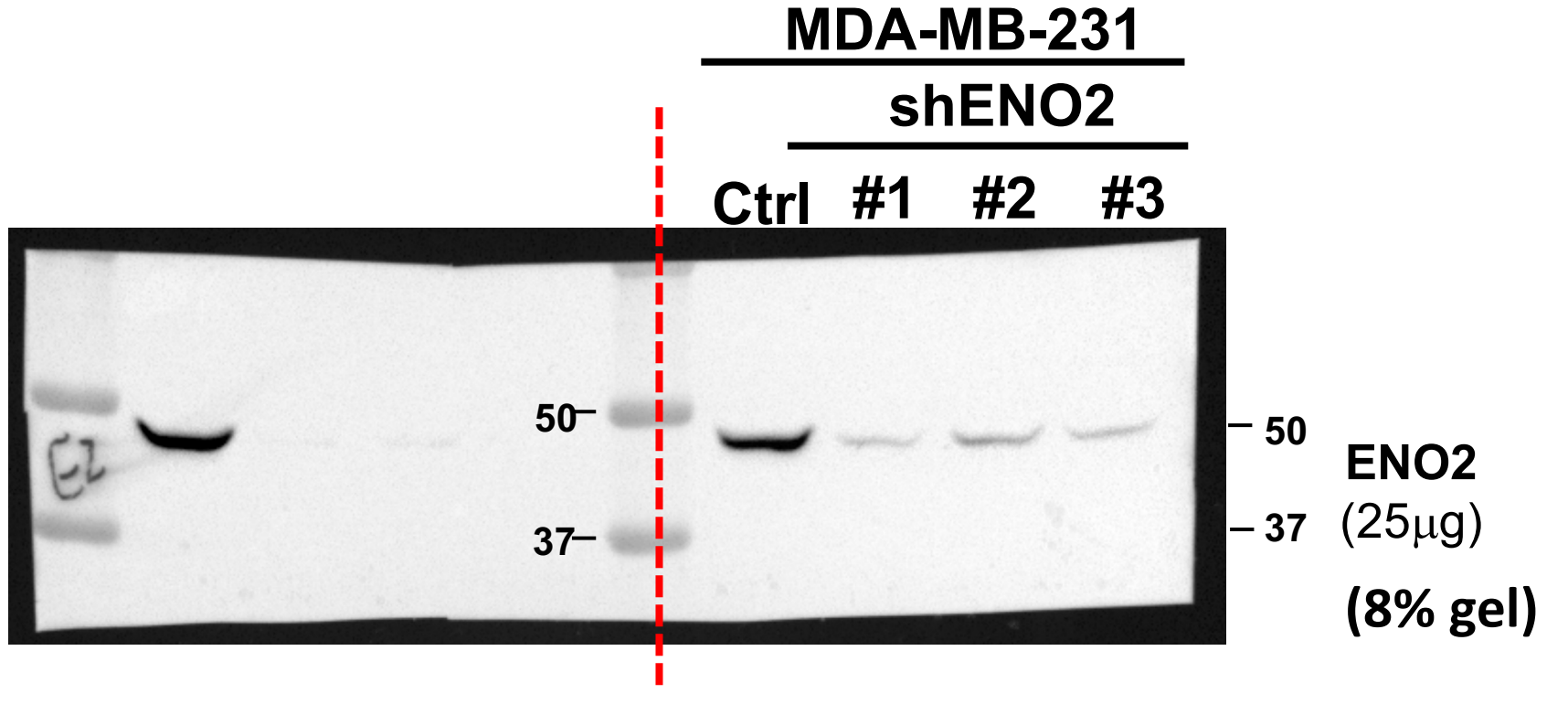

PAGE 3

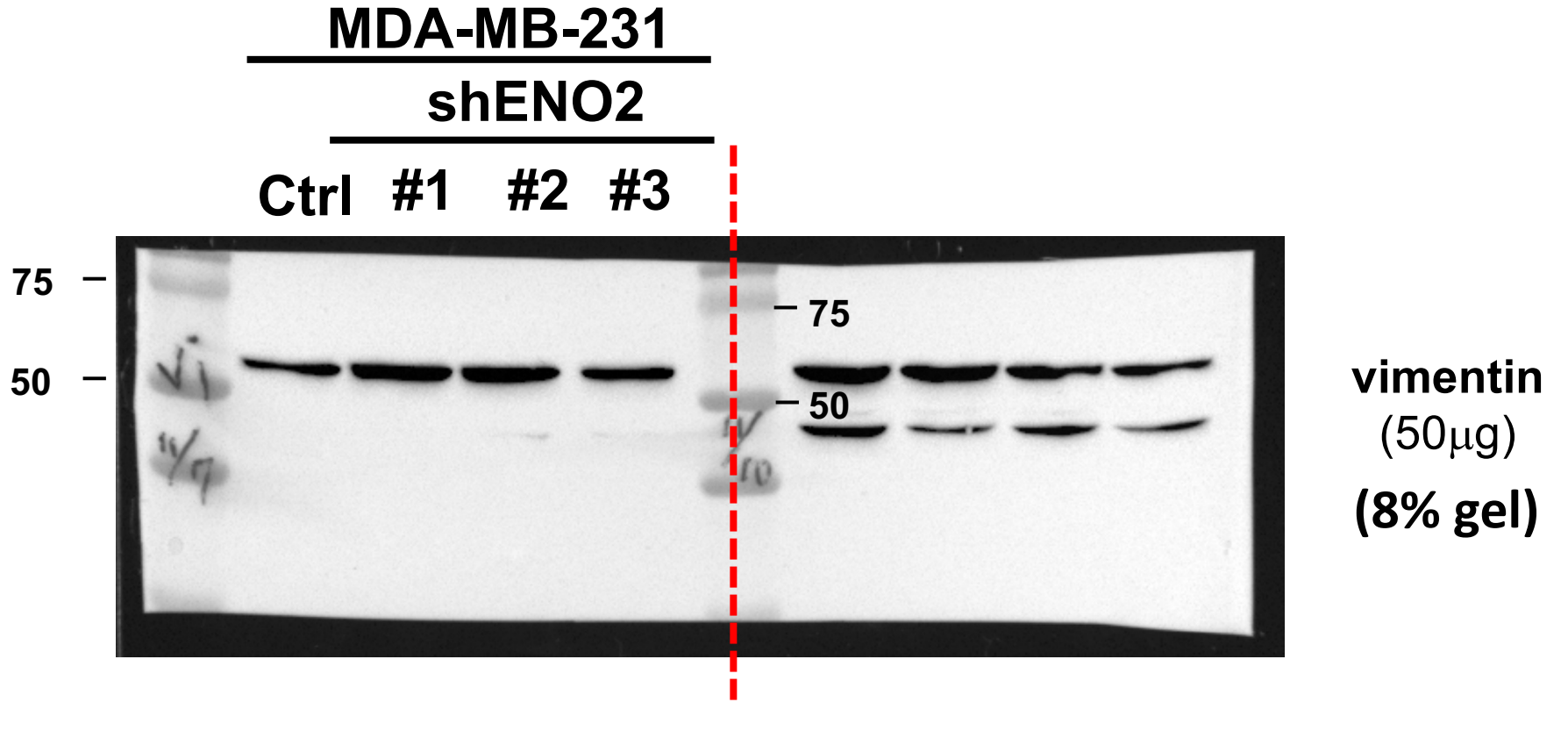

To produce data for Figure 3H, the samples were loading into PAGE 1, PAGE 2 and PAGE 3 with different protein amount (25 μg for PAGE 1 and PAGE 2, 50 μg for PAGE3). PAGE 1, PAGE 2 and PAGE3 were put into the same tank, and all the PAGEs were subjected to electrophoresis simultaneously. After protein transfer, membranes are sliced along the red dashed line and hybridized with indicated antibodies.

Uncropped images

Figure. 3J

PAGE 1

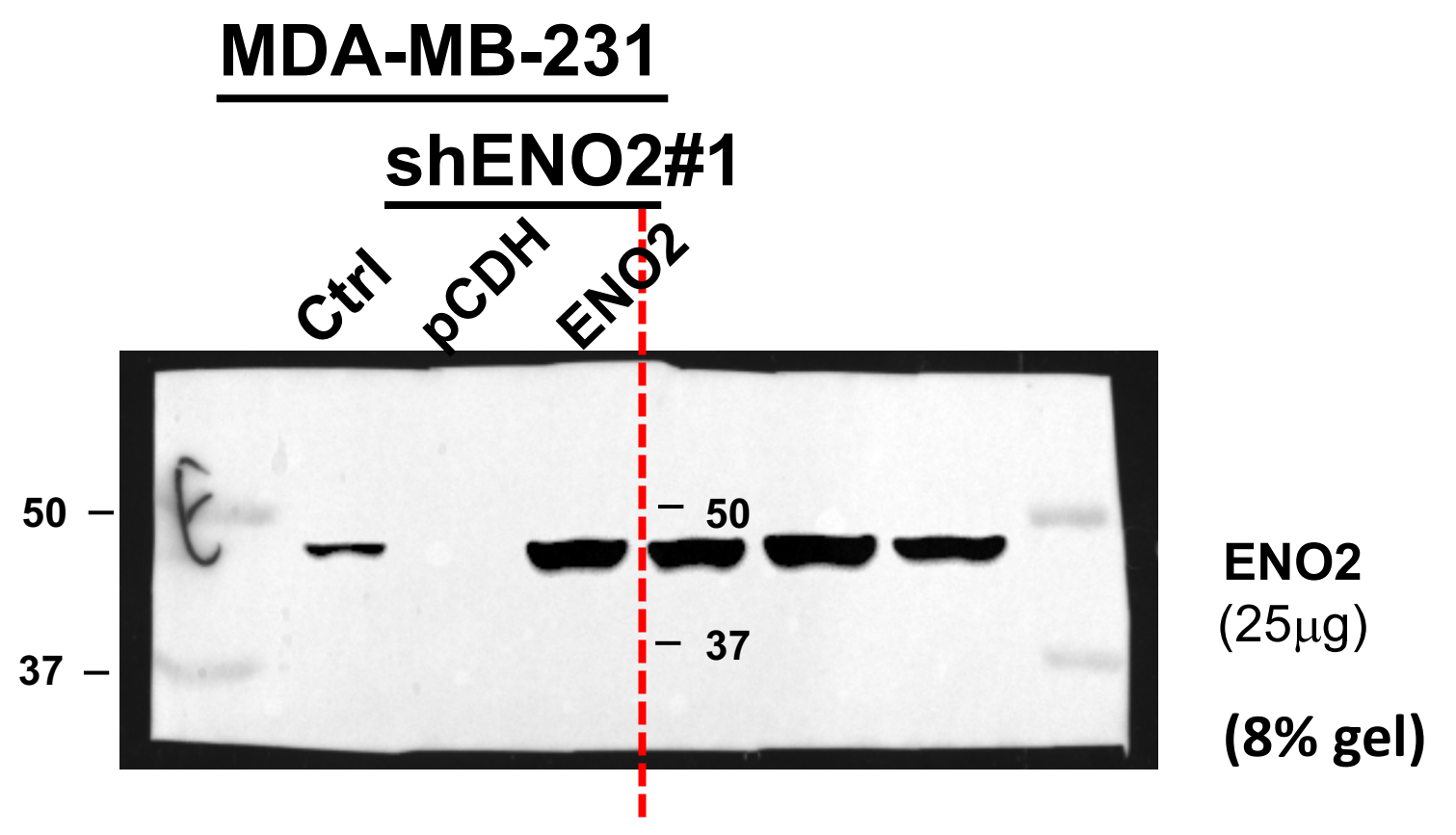

PAGE 2

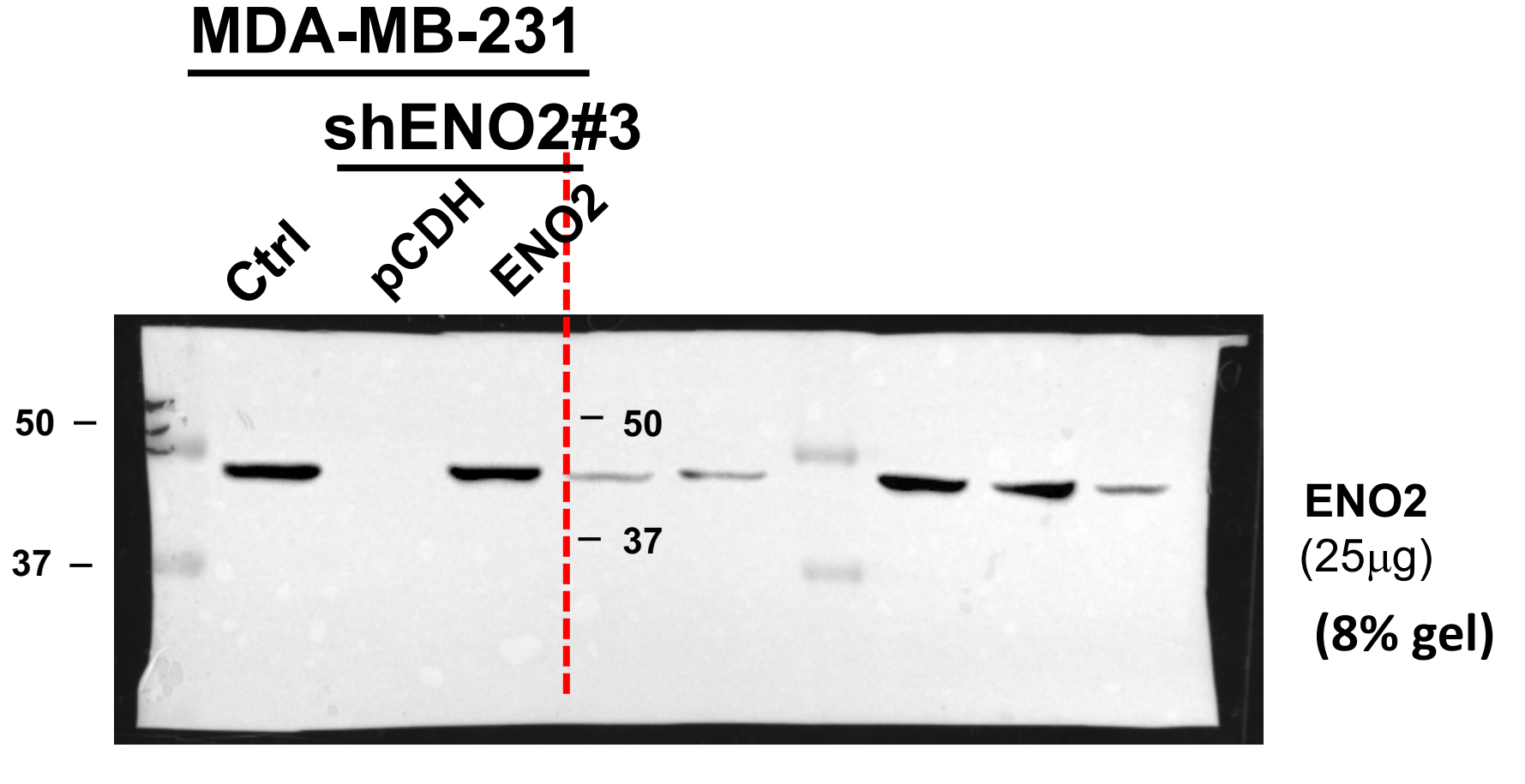

To produce data for Figure 3J, 25 μg samples were loading into PAGE 1 and PAGE 2. PAGE 1 and PAGE 2 were put into the same tank, and all the PAGEs were subjected to electrophoresis simultaneously. After protein transfer, membranes are sliced along the red dashed line and hybridized with indicated antibodies.

Uncropped images

Figure. 4K

PAGE 1

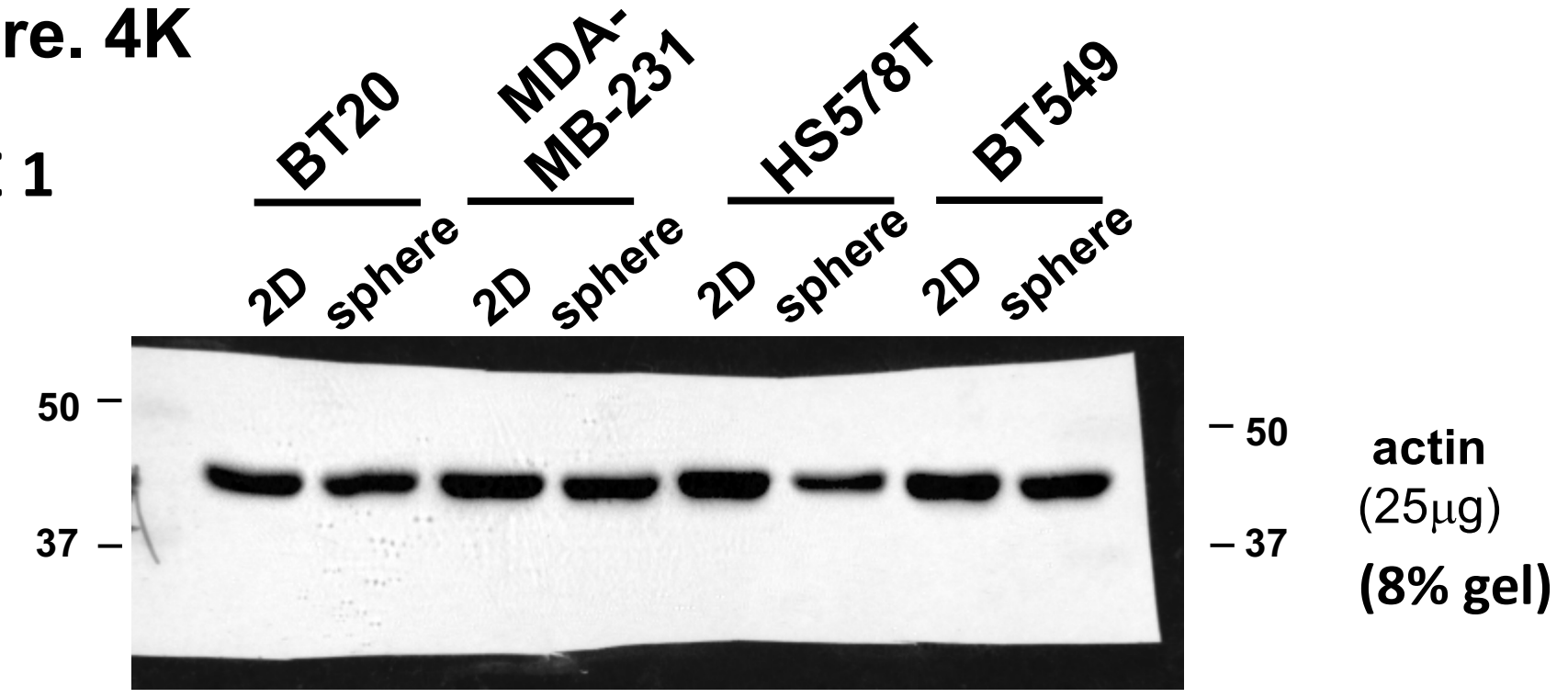

PAGE 2

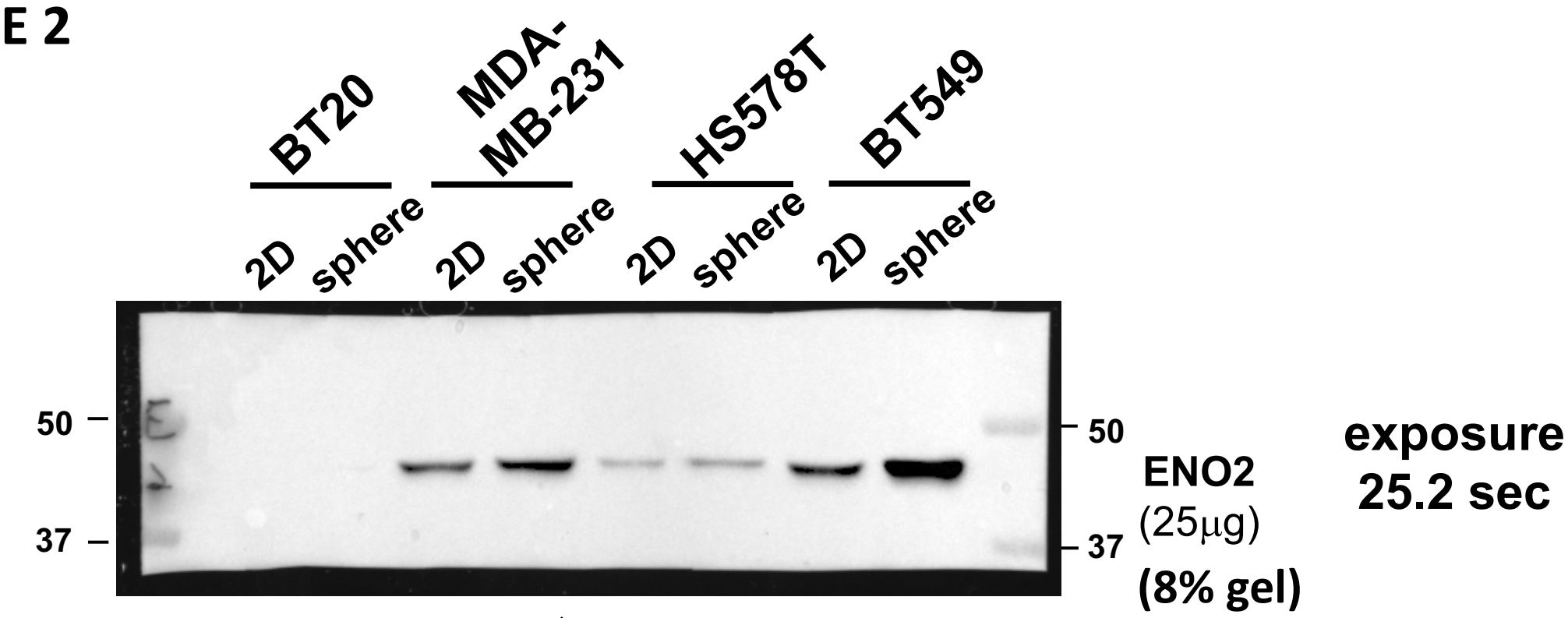

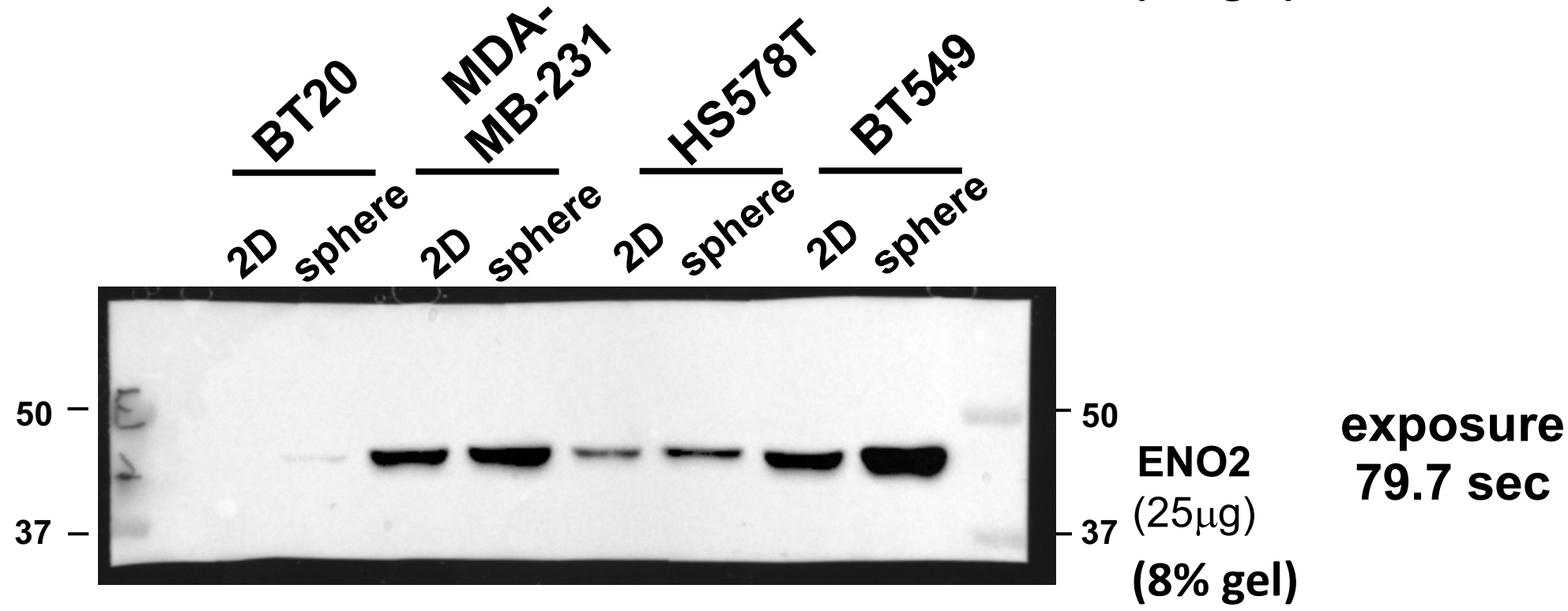

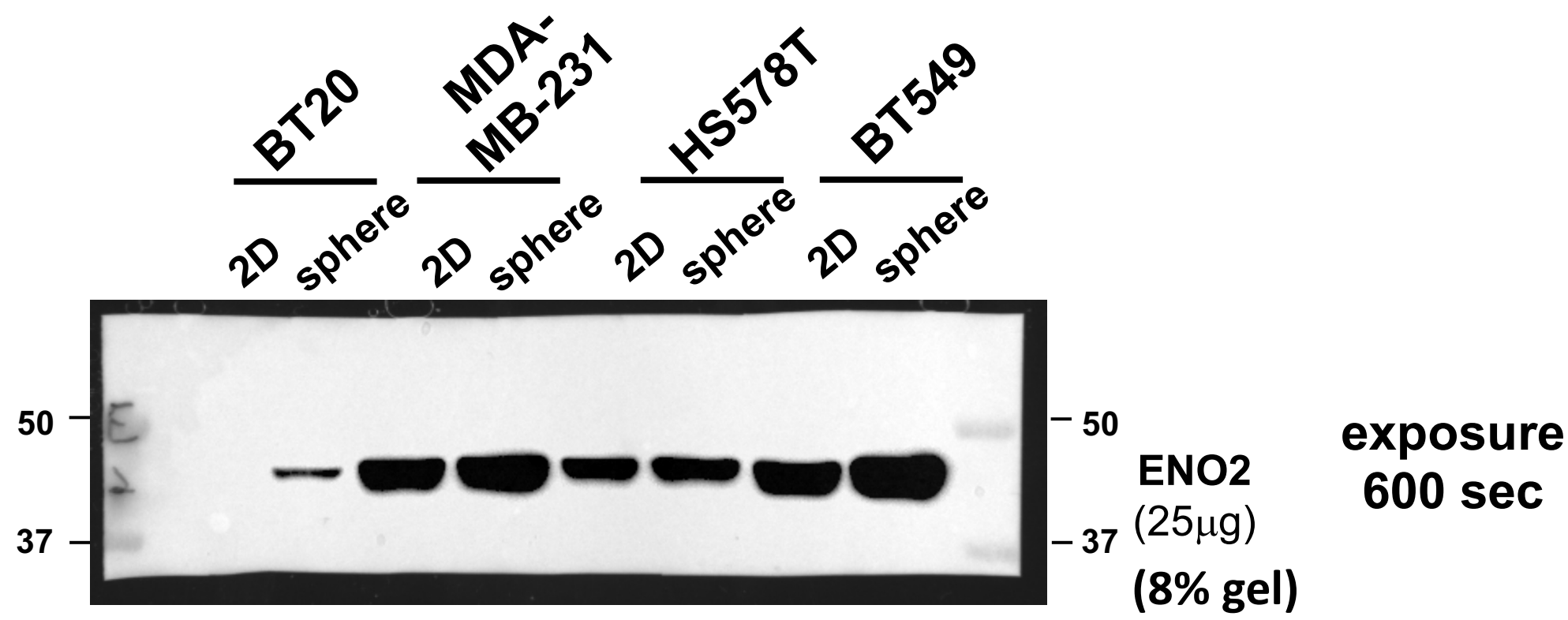

To produce data for Figure 4K, 25 μg samples were loading into PAGE 1 and PAGE 2. PAGE 1 and PAGE 2 were put into the same tank, and all the PAGEs were subjected to electrophoresis simultaneously. After protein transfer, membranes are sliced and hybridized with indicated antibodies.

Uncropped images

Figure. 4K

PAGE 1

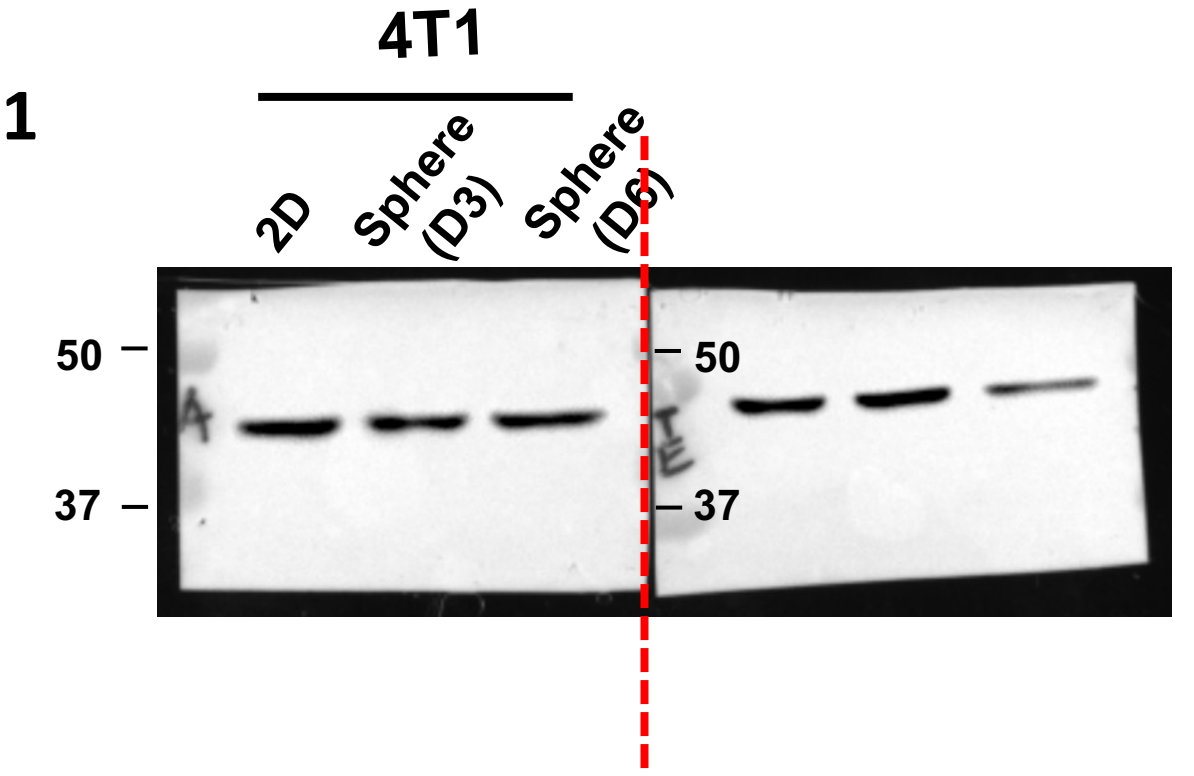

actin
(25μg)

(8% gel)

PAGE 2

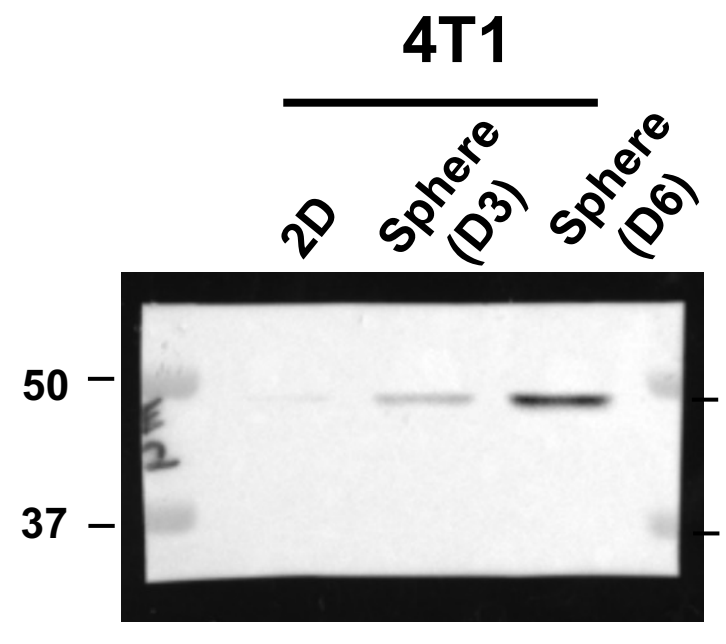

ENO2
(25μg)
(8% gel)

exposure
61.5 sec

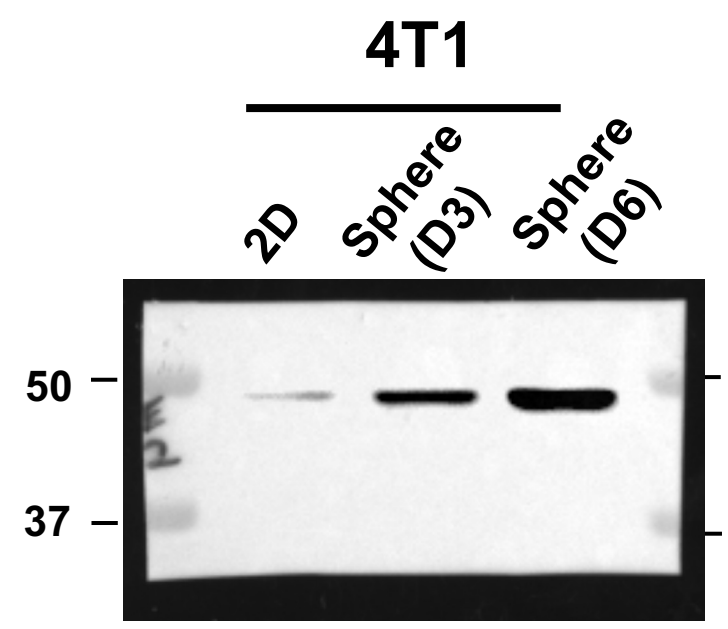

ENO2
(25μg)
(8% gel)

exposure
404.2 sec

To produce data for Figure 4K, 25 μg samples were loading into PAGE 1 and PAGE 2. PAGE 1 and PAGE 2 were put into the same tank, and all the PAGEs were subjected to electrophoresis simultaneously. After protein transfer, membranes are sliced along the red dashed line and hybridized with indicated antibodies.

Uncropped images

Figure. 5K

PAGE 1

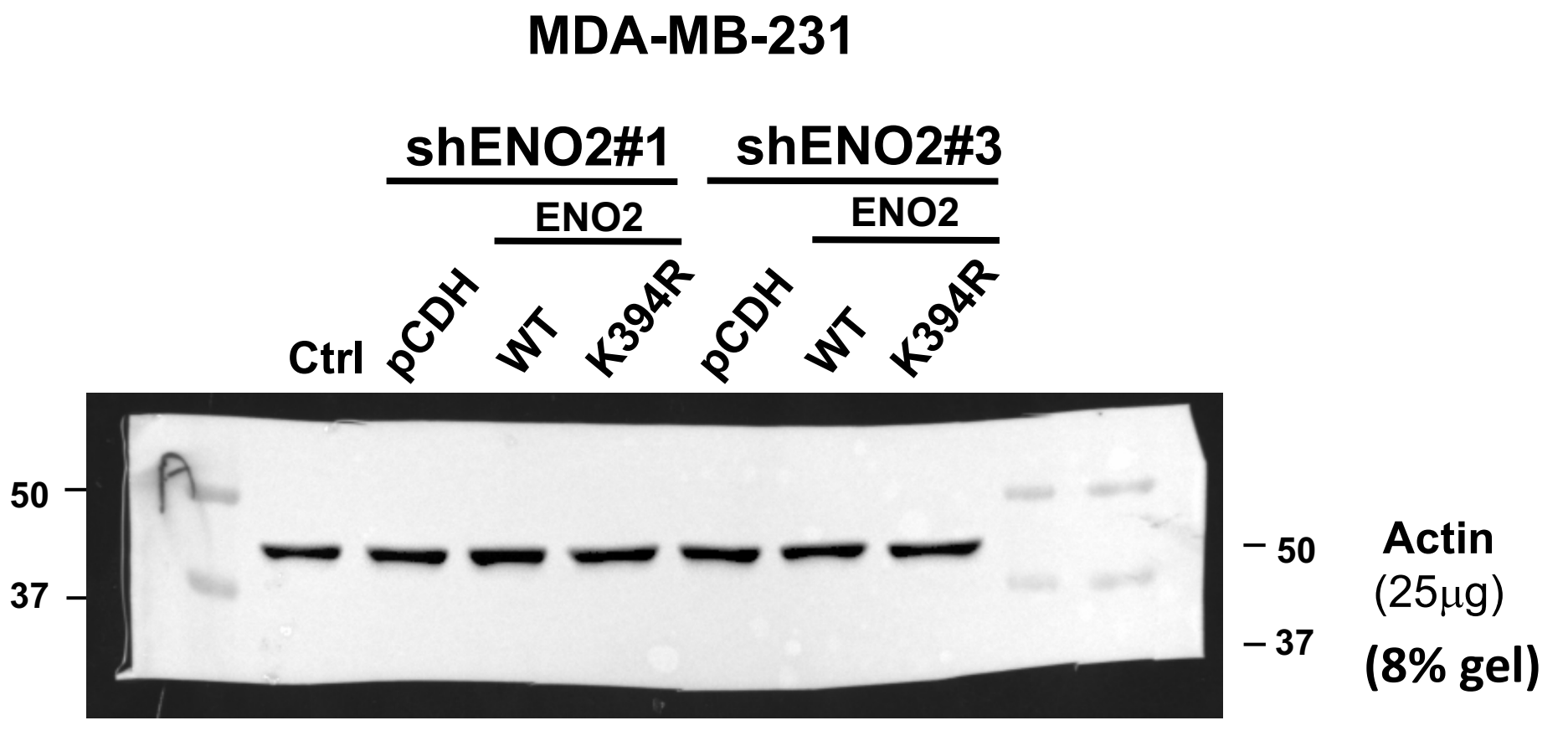

PAGE 2

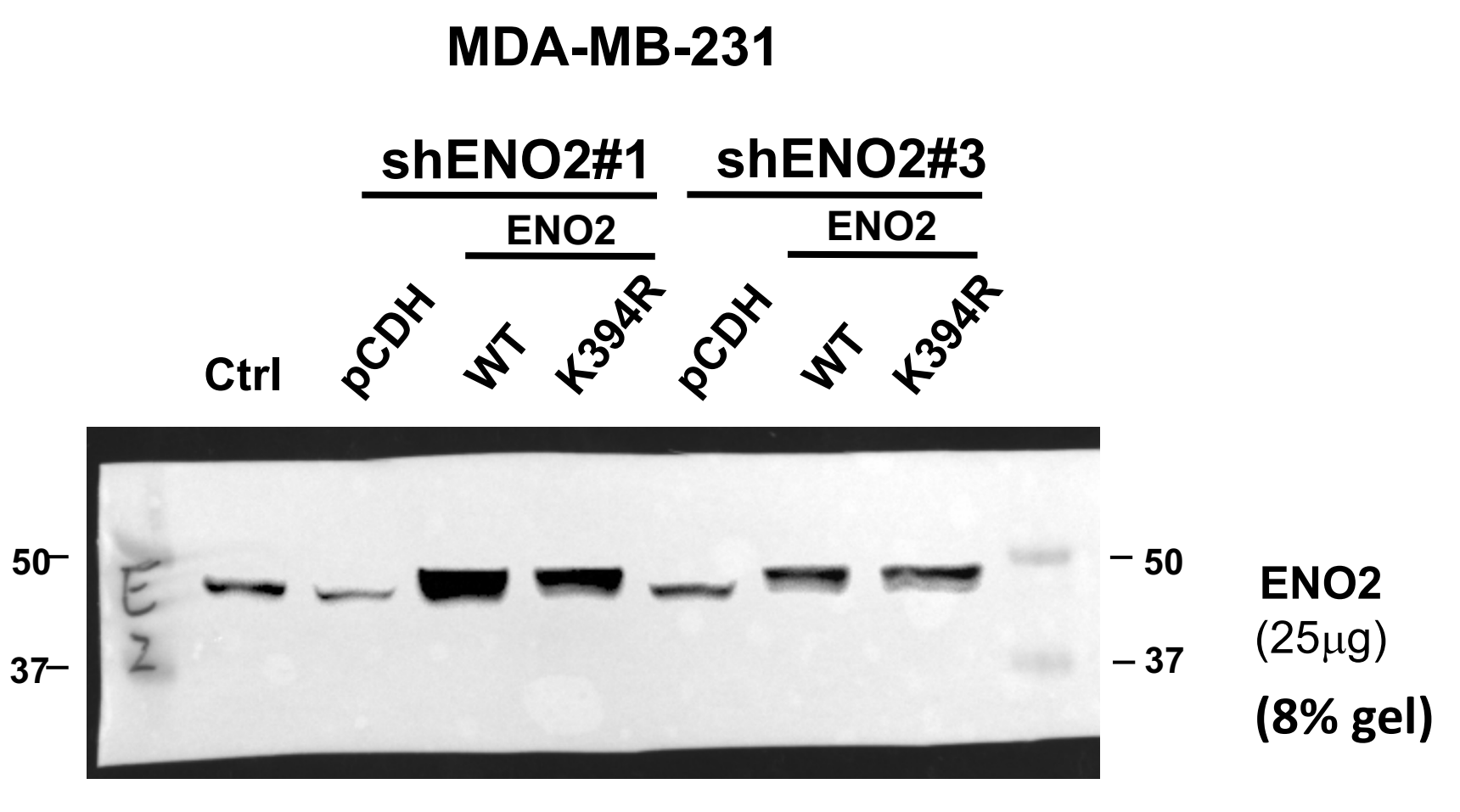

To produce data for Figure 5K, 25 μg samples were loading into PAGE 1 and PAGE 2. PAGE 1 and PAGE 2 were put into the same tank, and all the PAGEs were subjected to electrophoresis simultaneously. After protein transfer, membranes are sliced and hybridized with indicated antibodies.

Uncropped images

Figure. 6I

PAGE 1

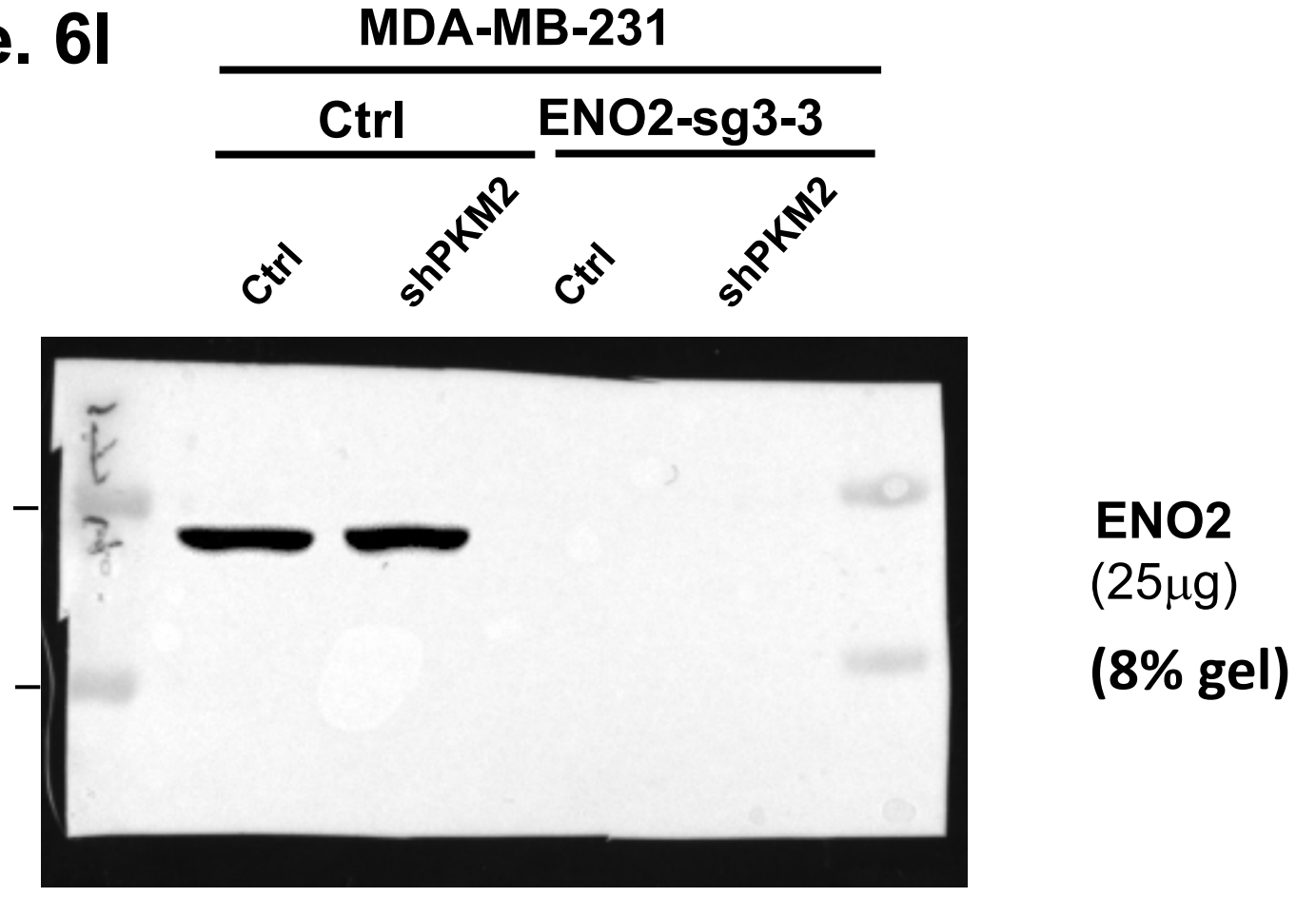

PAGE 2

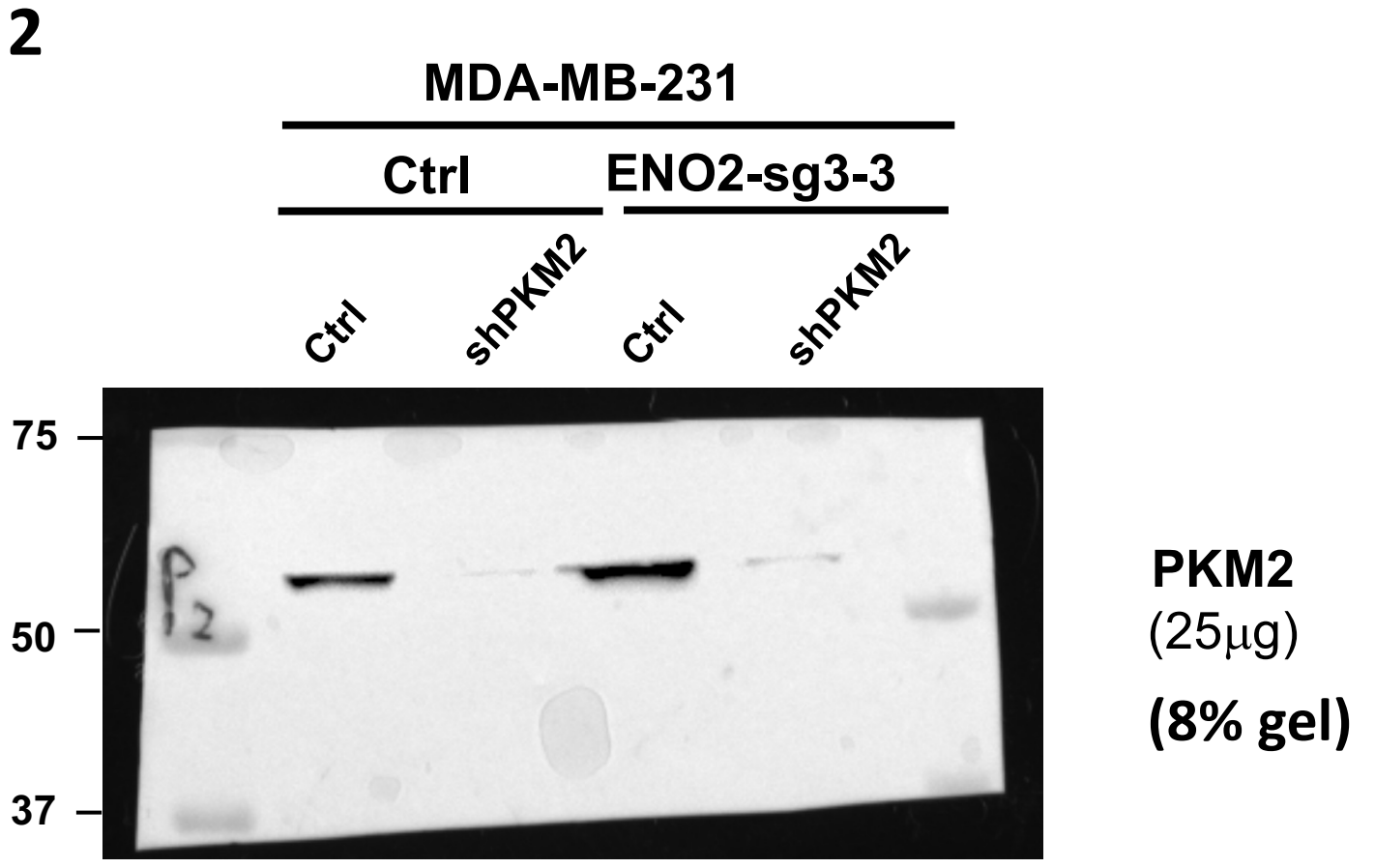

PAGE 3

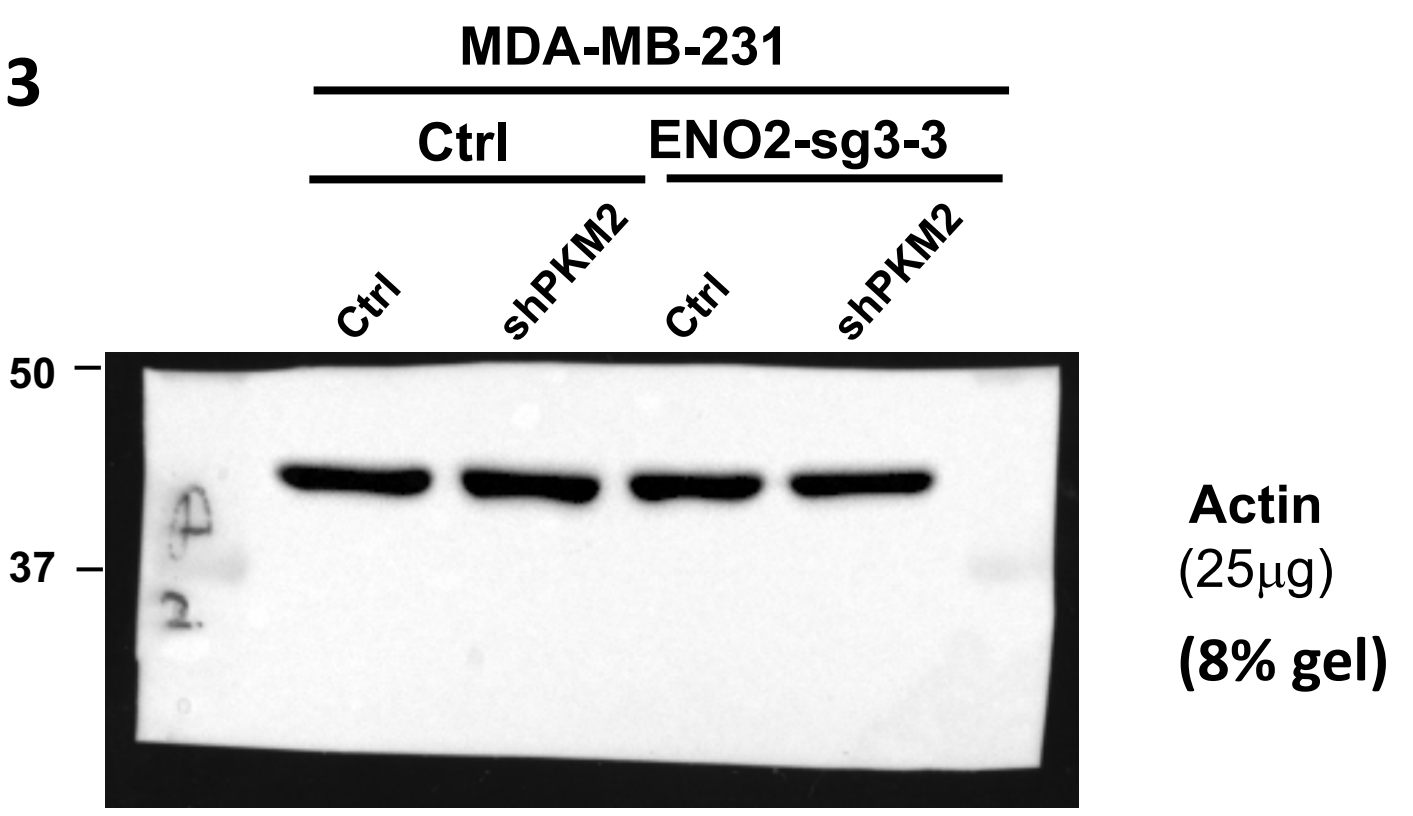

To produce data for Figure 6I, 25 μg samples were loading into PAGE 1 ,PAGE 2 and PAGE 3 . All PAGEs were put into the same tank, and were subjected to electrophoresis simultaneously. After protein transfer, membranes are sliced and hybridized with indicated antibodies.

Uncropped images

Figure. 6M

PAGE 1

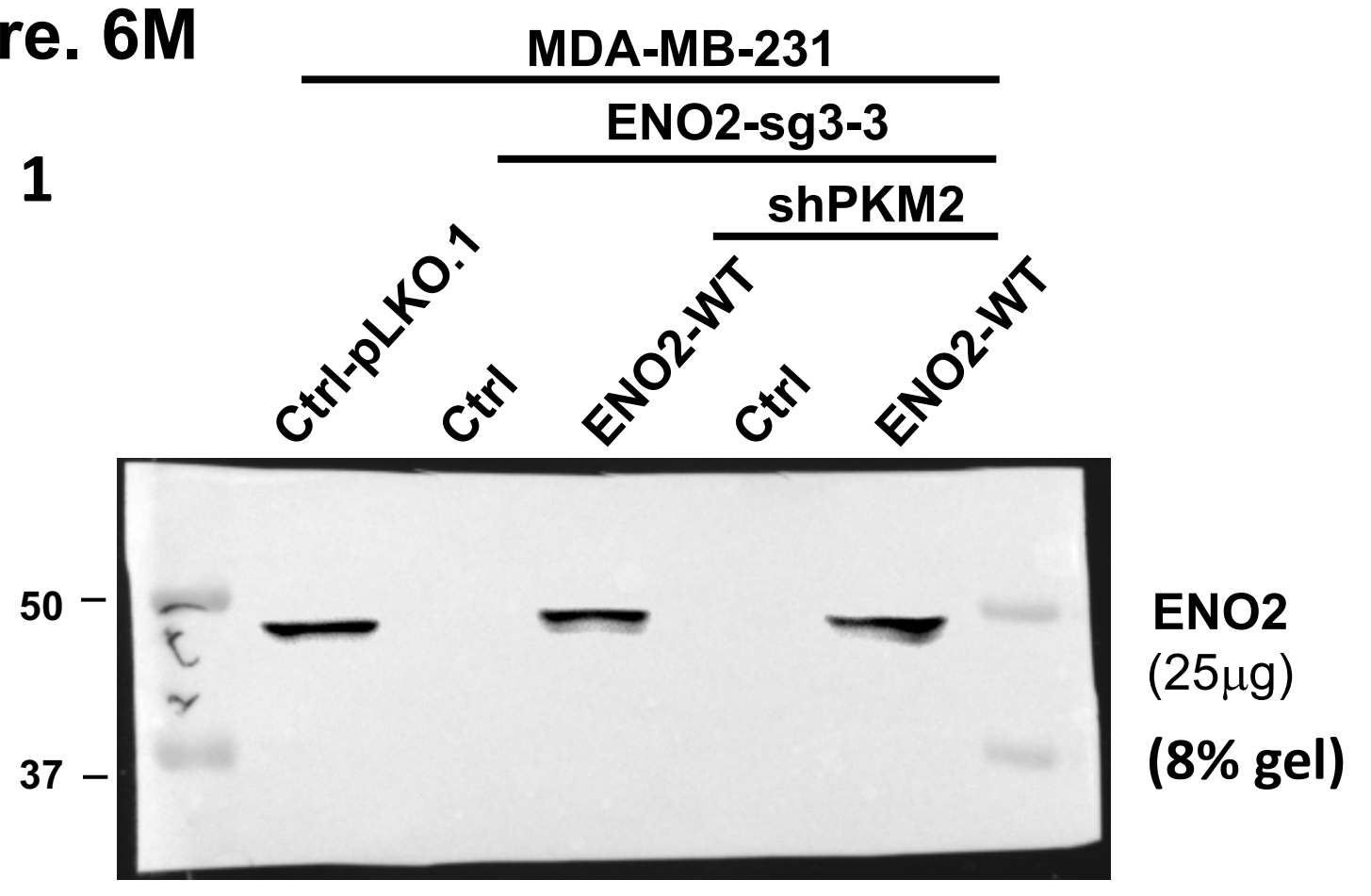

PAGE 2

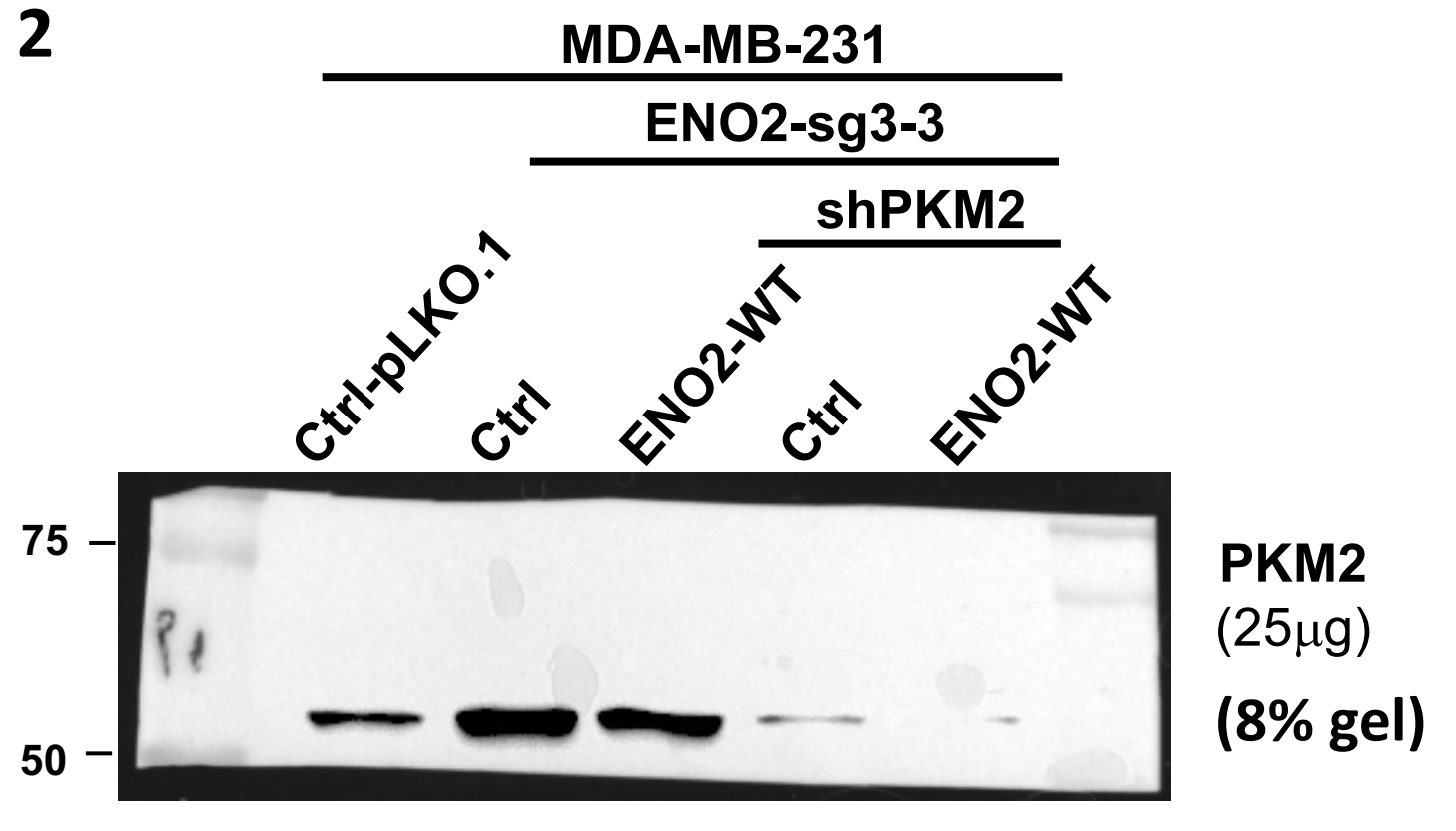

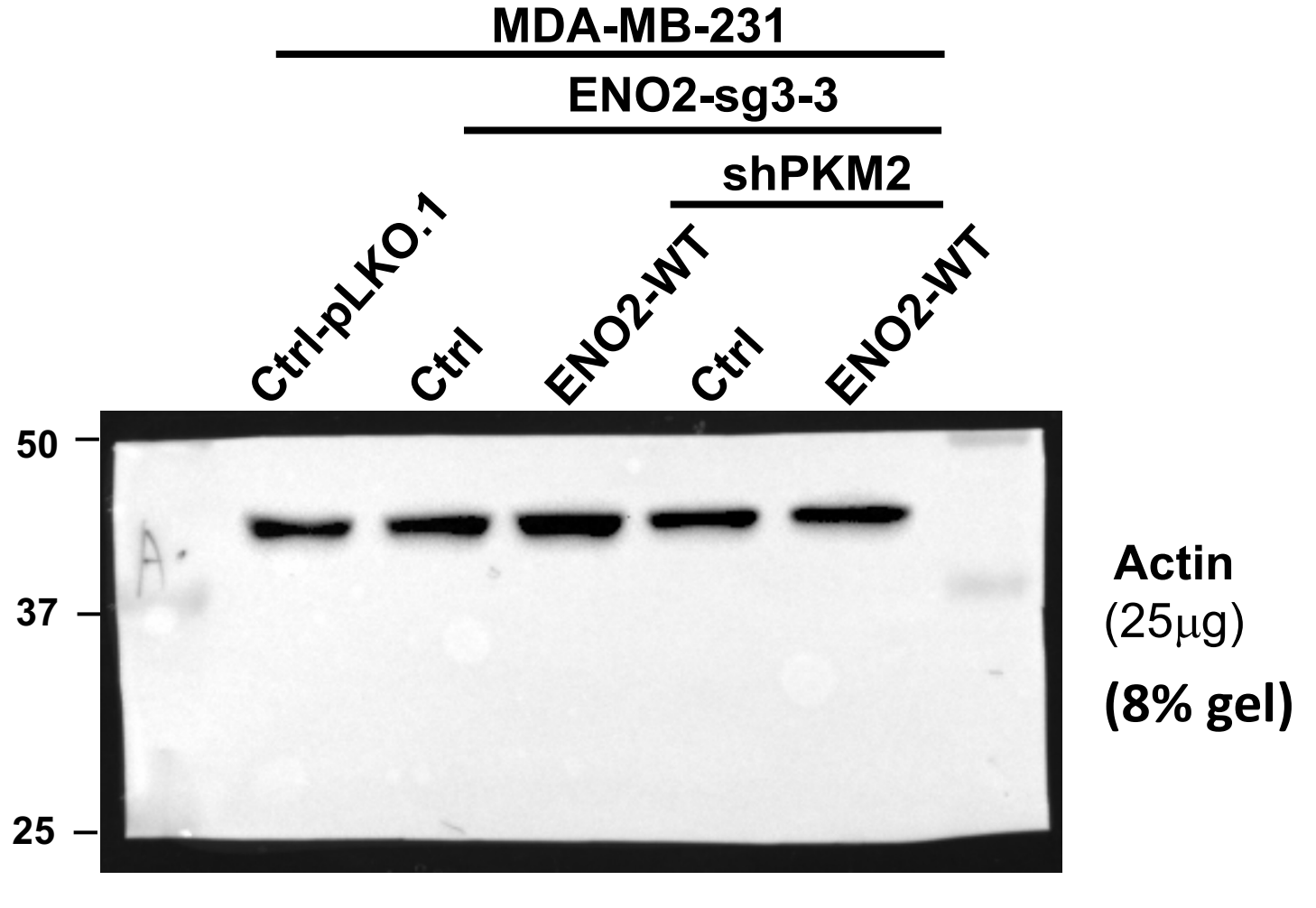

To produce data for Figure 6M, 25 μg samples were loading into PAGE 1 and PAGE 2. PAGE 1 and PAGE 2 were put into the same tank, and all the PAGEs were subjected to electrophoresis simultaneously. After protein transfer, membranes are sliced and hybridized with indicated antibodies.

Uncropped images

Supplementary Figure S1

PAGE 1

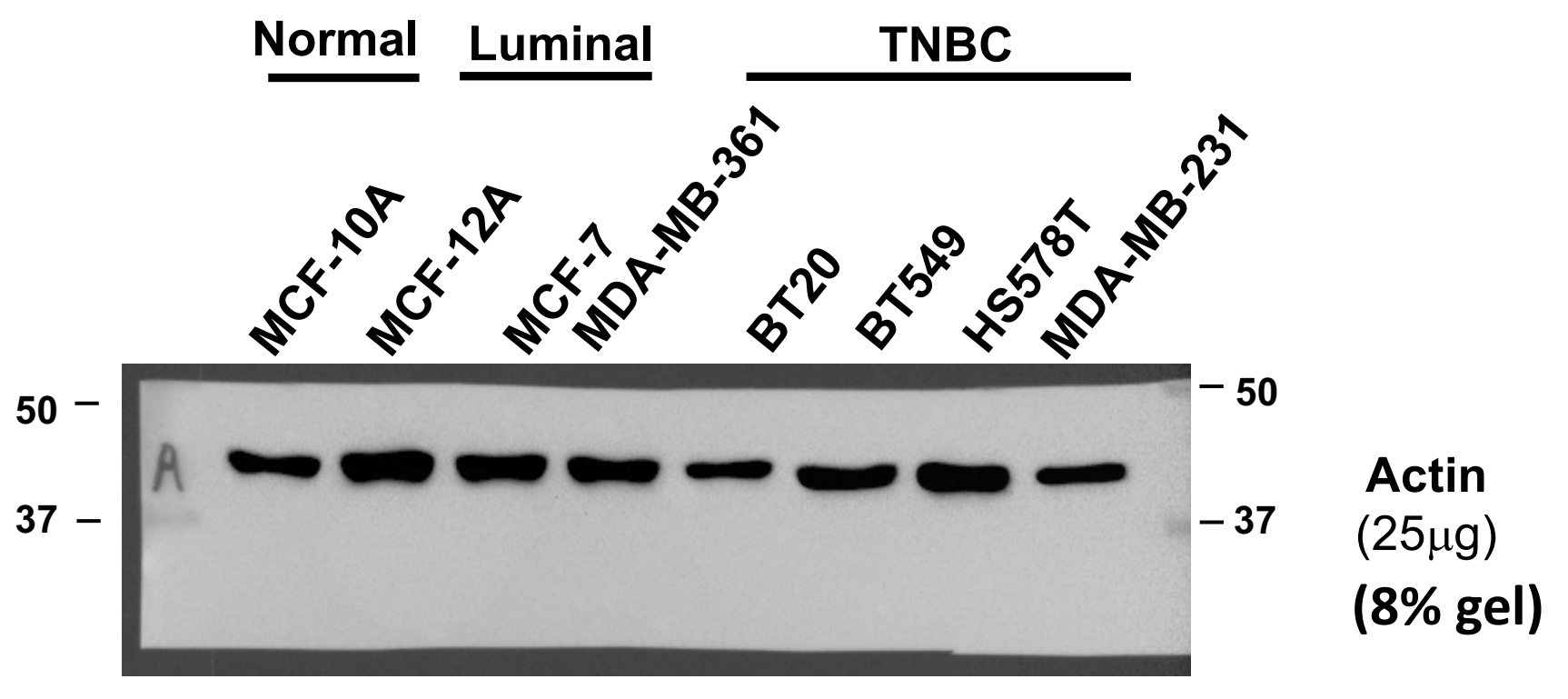

PAGE 2

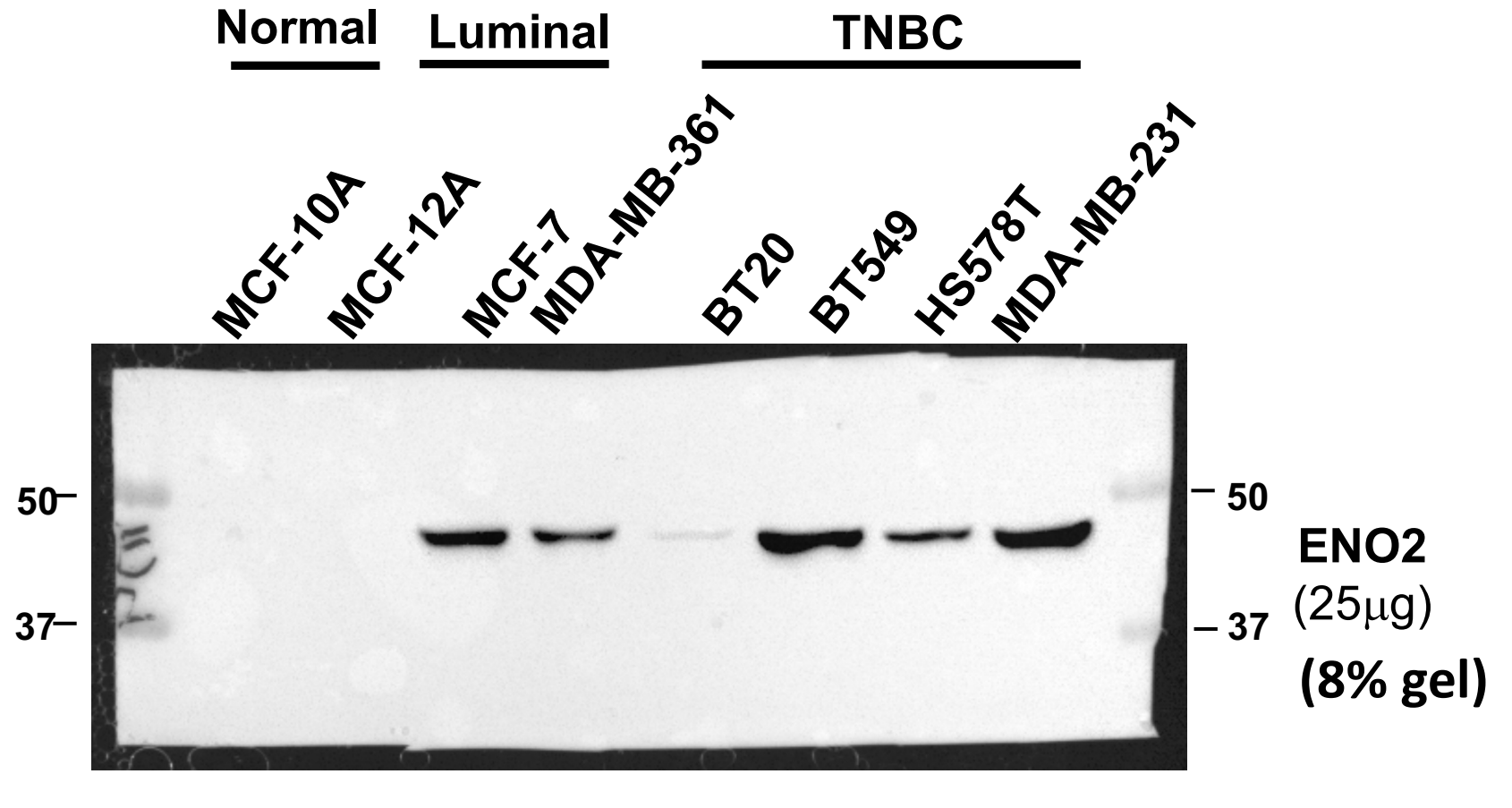

To produce data for Supplementary Figure S1, 25 μg samples were loading into PAGE 1 and PAGE 2. PAGE 1 and PAGE 2 were put into the same tank, and all the PAGEs were subjected to electrophoresis simultaneously. After protein transfer, membranes are sliced along the red dashed line and hybridized with indicated antibodies.

Uncropped images

Supplementary Figure S8

PAGE 1

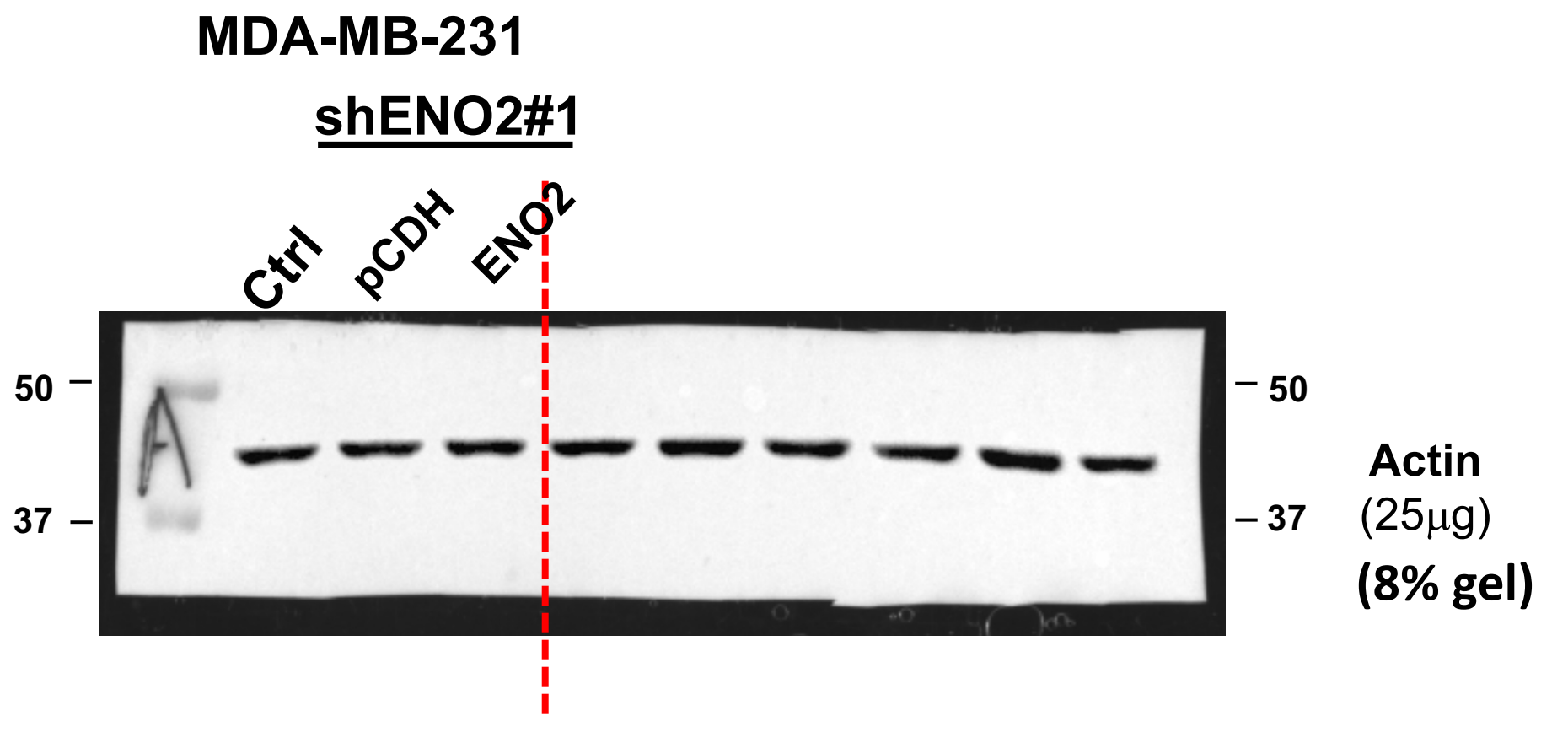

PAGE 2

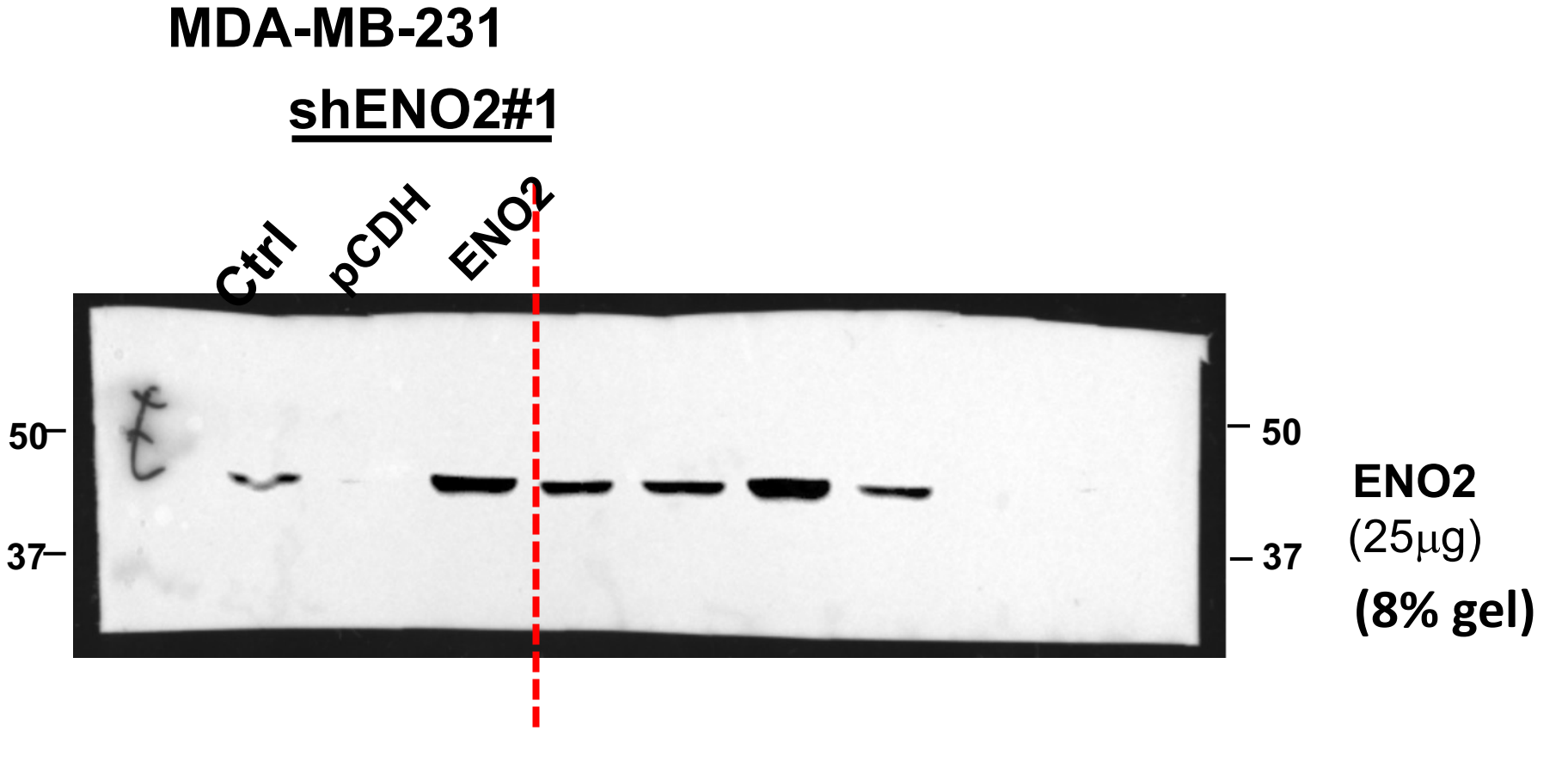

To produce data for Supplementary Figure S8, 25 µg samples were loading into PAGE 1 and PAGE 2. PAGE 1 and PAGE 2 were put into the same tank, and all the PAGEs were subjected to electrophoresis simultaneously. After protein transfer, membranes are sliced along the red dashed line and hybridized with indicated antibodies.

Uncropped images

Supplementary Figure S10

PAGE 1

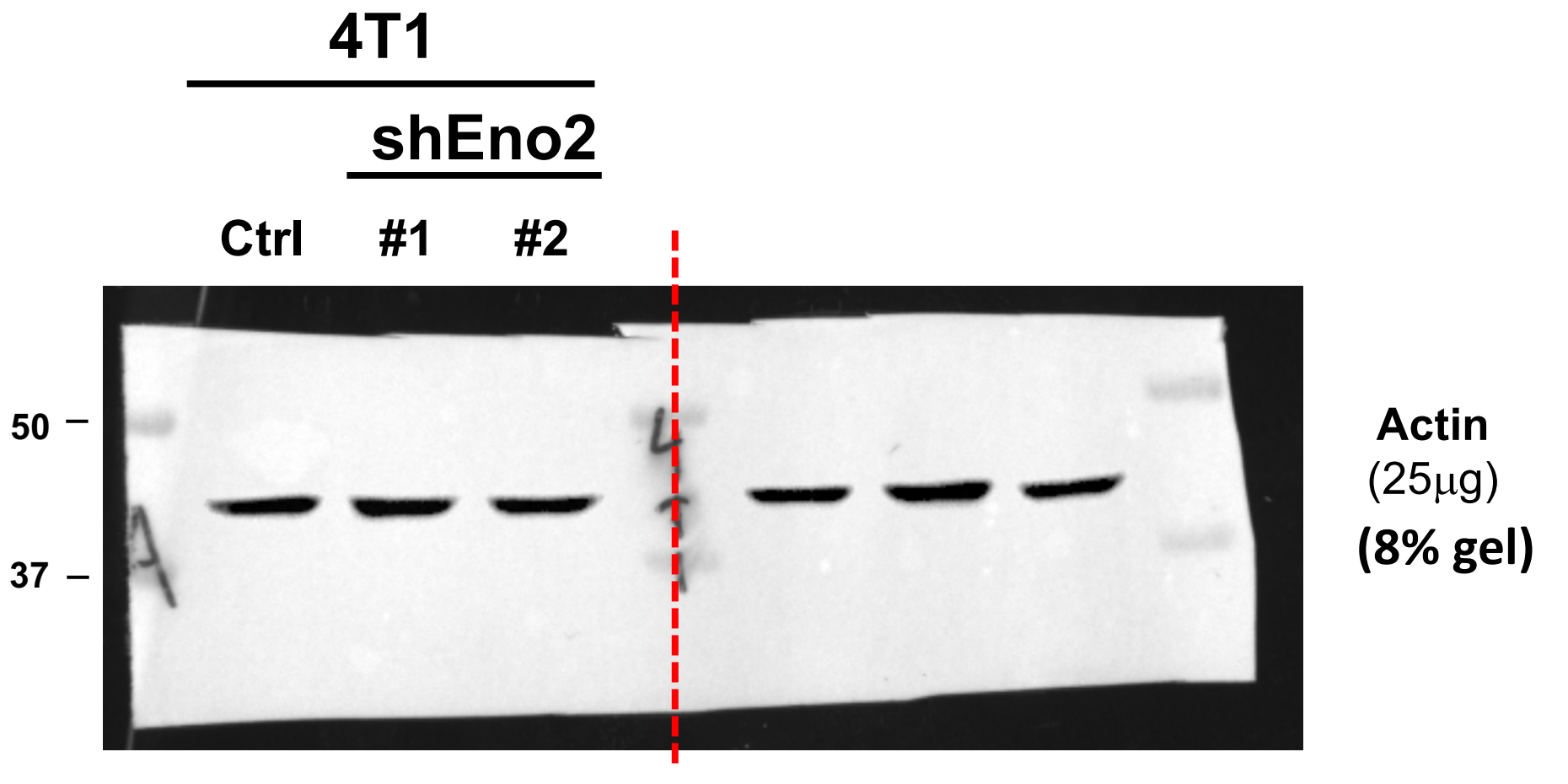

PAGE 2

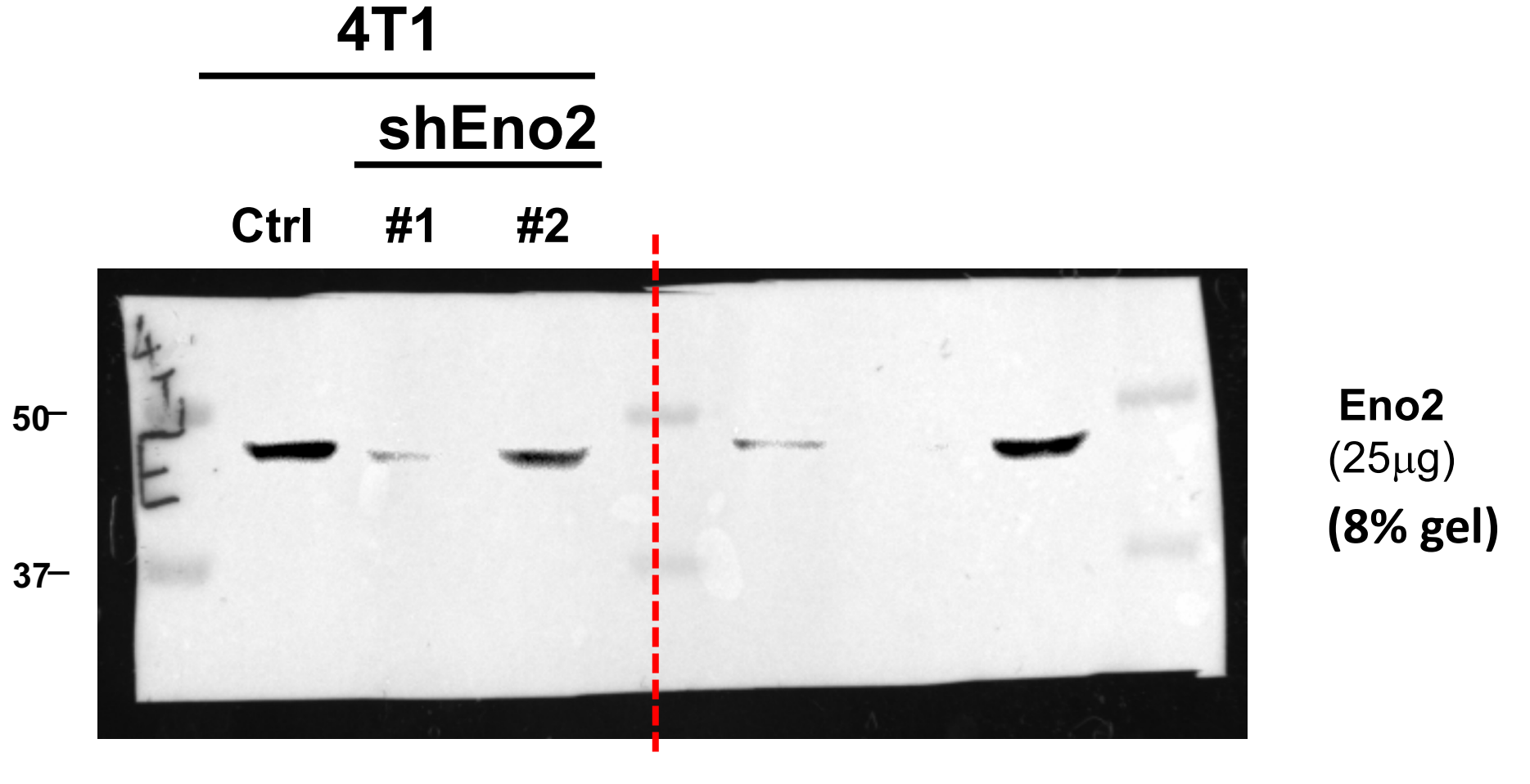

To produce data for Supplementary Figure S10, 25 µg samples were loading into PAGE 1 and PAGE 2. PAGE 1 and PAGE 2 were put into the same tank, and all the PAGEs were subjected to electrophoresis simultaneously. After protein transfer, membranes are sliced along the red dashed line and hybridized with indicated antibodies.

Uncropped images

Supplementary Figure S15A

PAGE 1

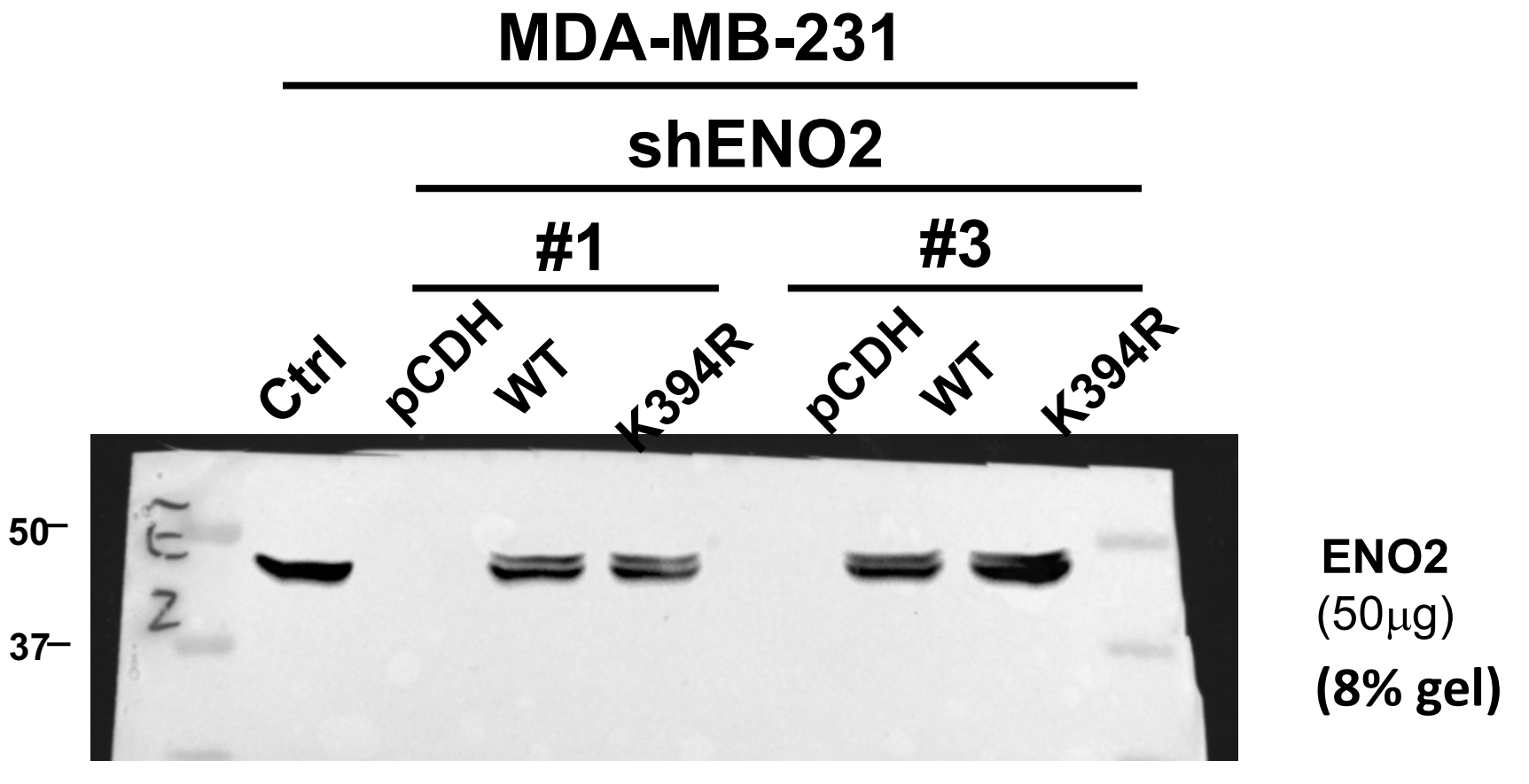

To produce data for Supplementary Figure S15A, 25 μg samples were loading into PAGE 1 and the PAGE was subjected to electrophoresis. After protein transfer, membrane is sliced and hybridized with indicated antibodies.

Uncropped images

Supplementary Figure S15B

PAGE 1

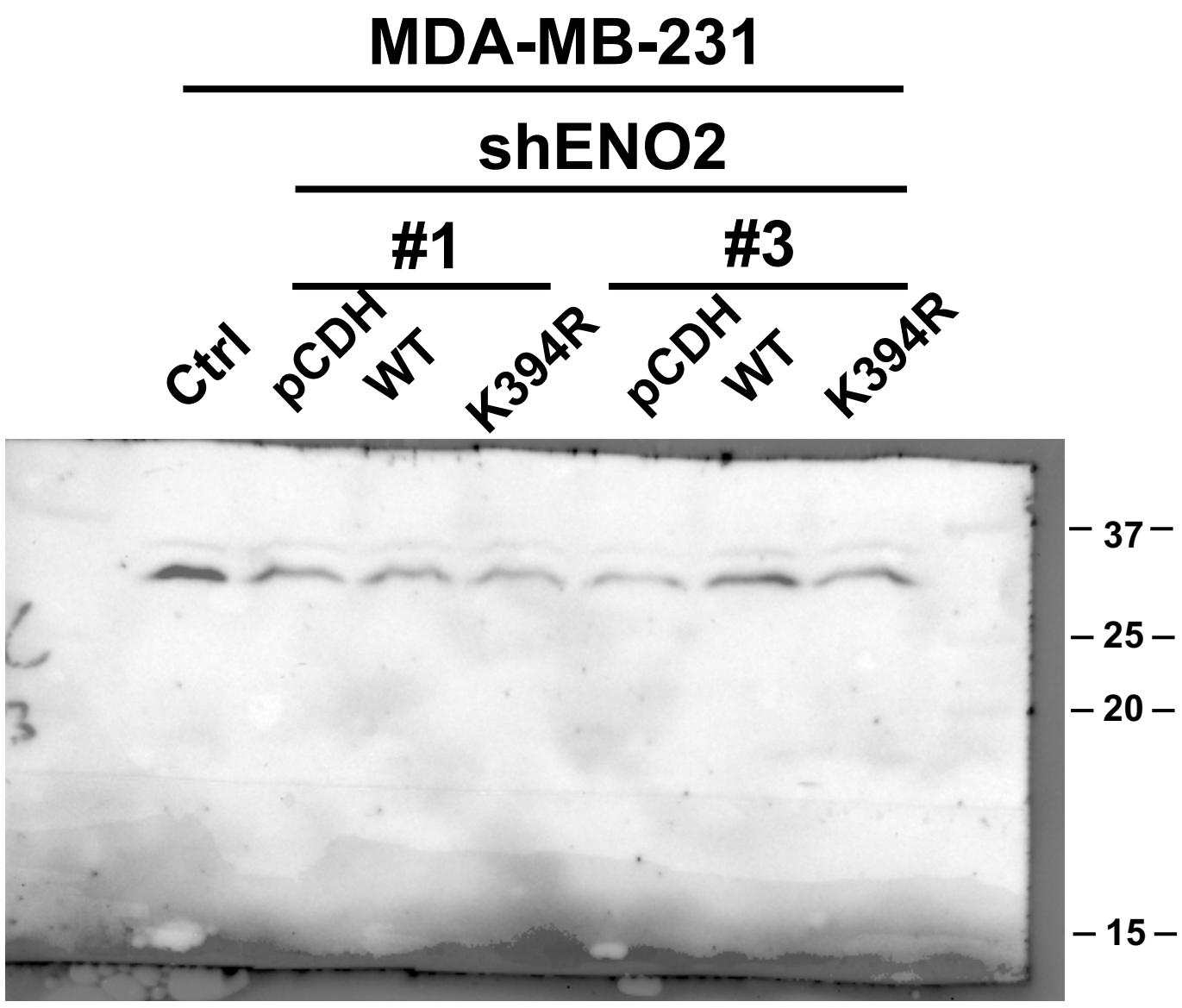

Shorter exposure (510 sec)

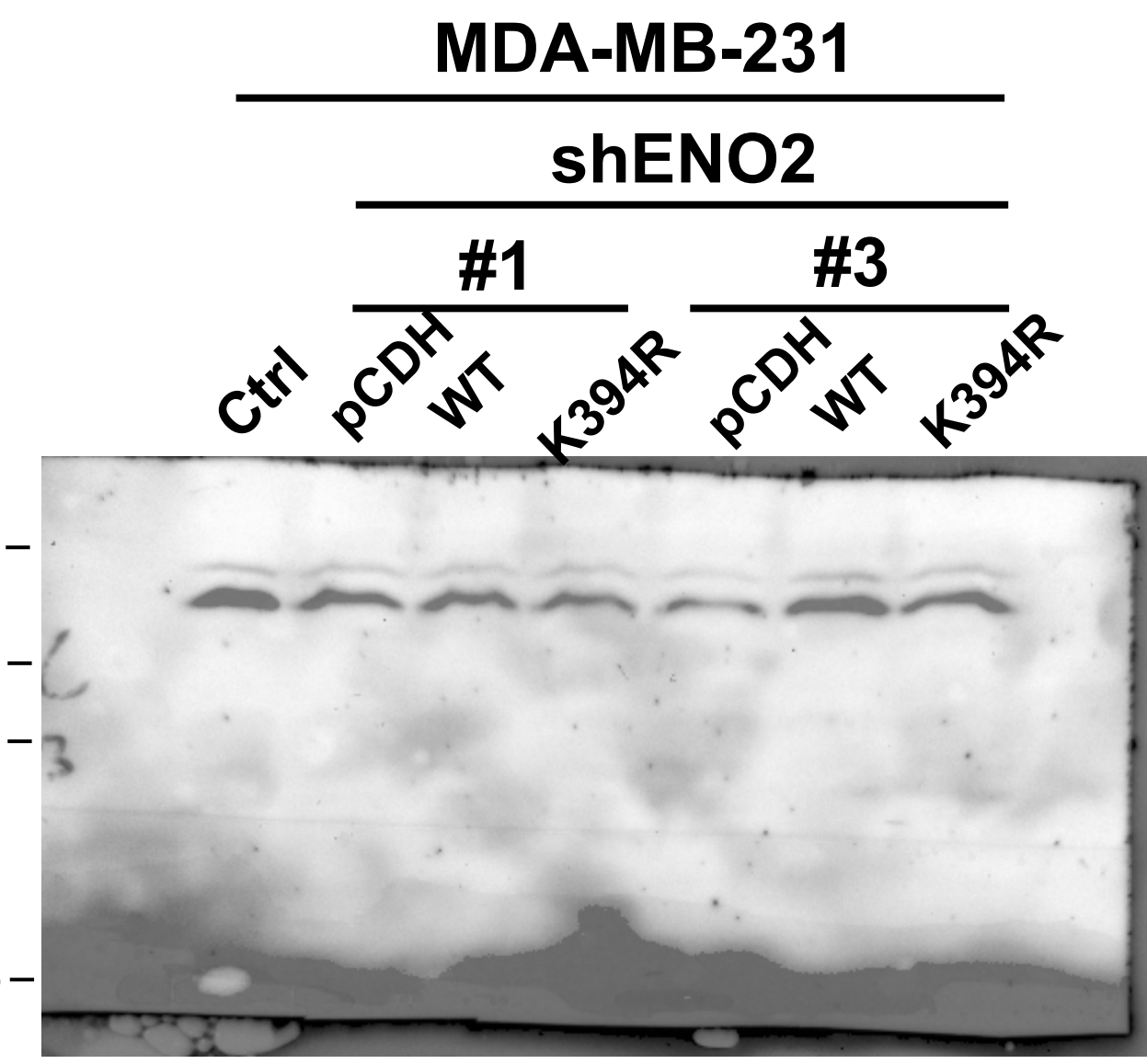

Over exposure (1019 sec)

Caspase 3
(100μg)
(12% gel)

PAGE 2

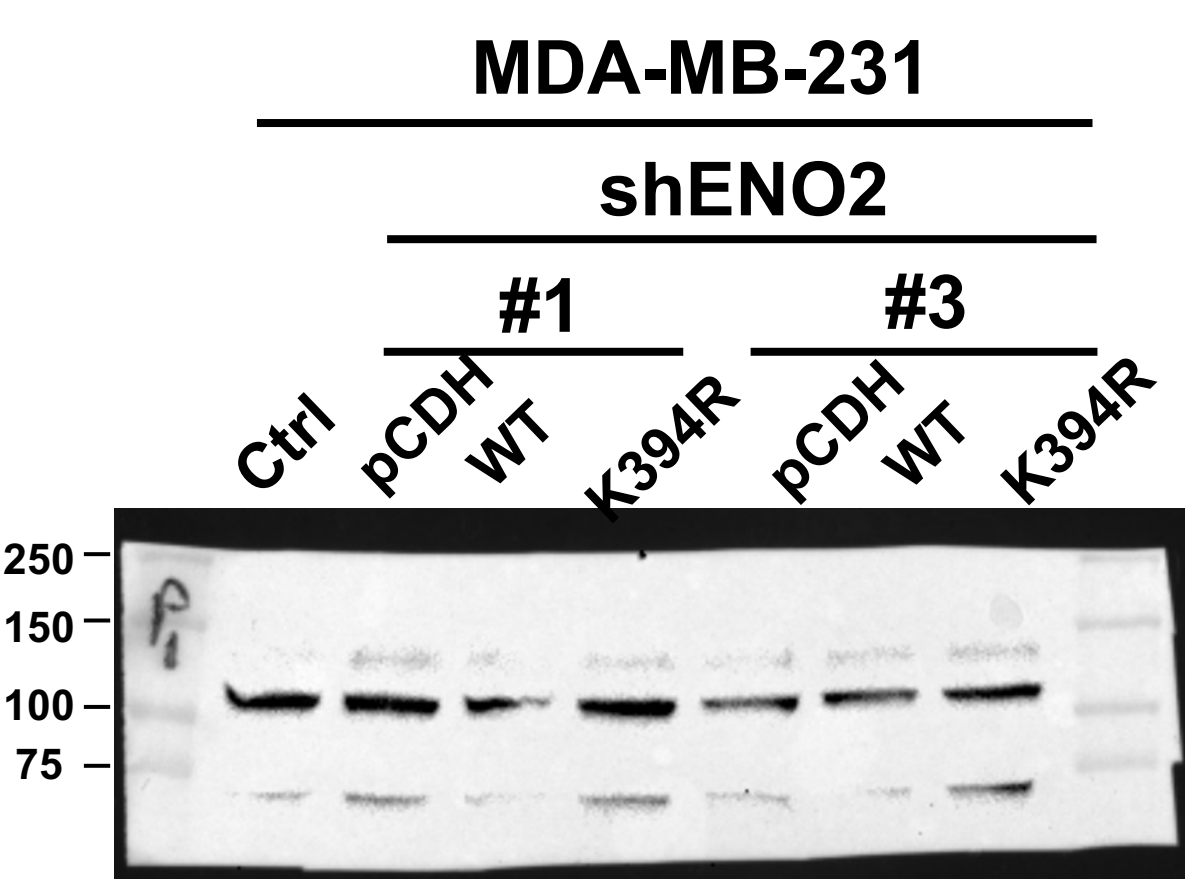

Shorter exposure (194.9 sec)

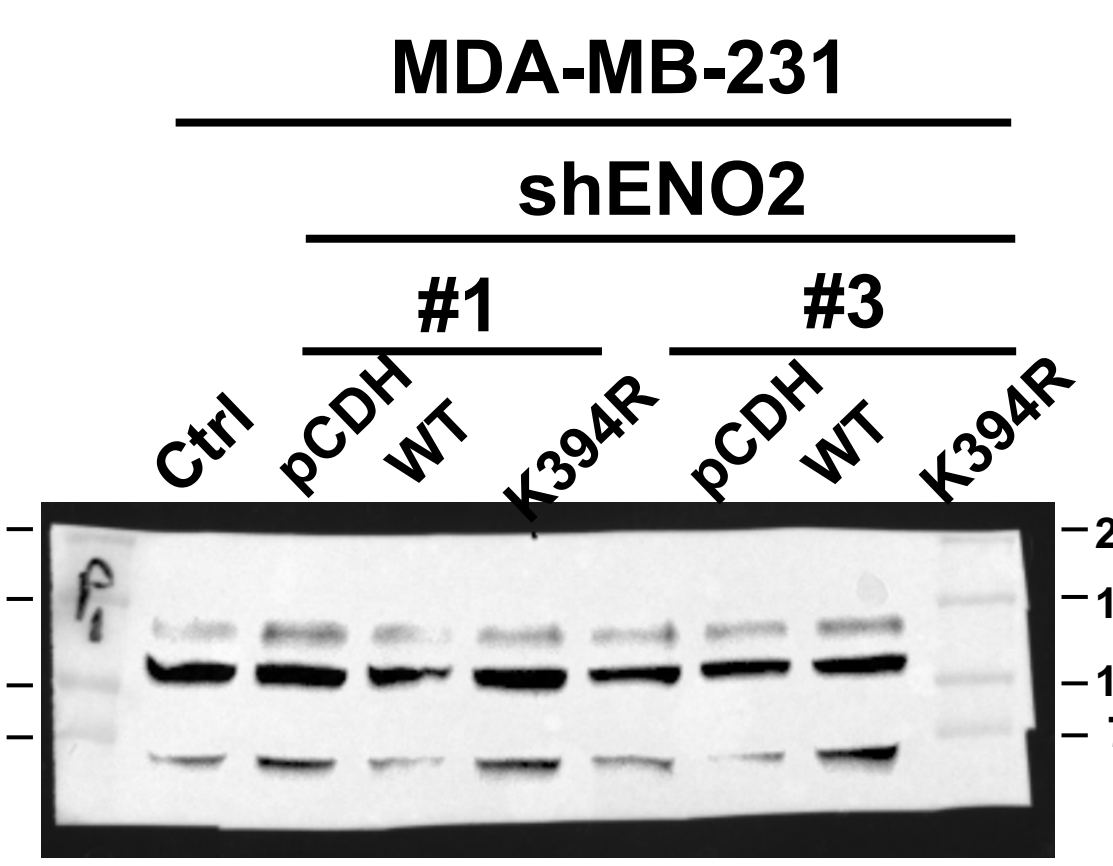

Over exposure (2036.5 sec)

PARP-1
(100μg)
(8% gel)

PAGE 3

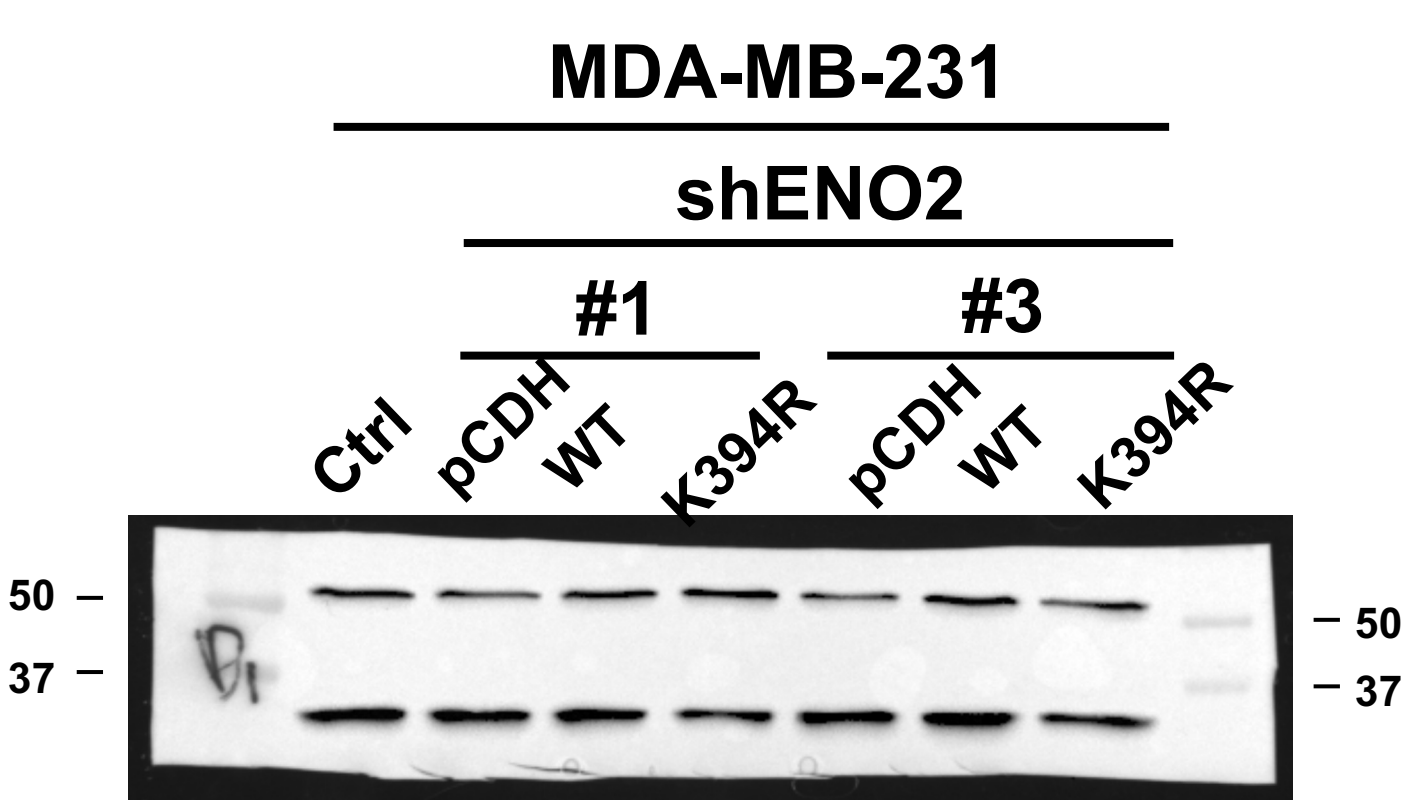

Cyclin D1
(50μg)
(12% gel)

Uncropped images

Supplementary Figure S15B

PAGE 4

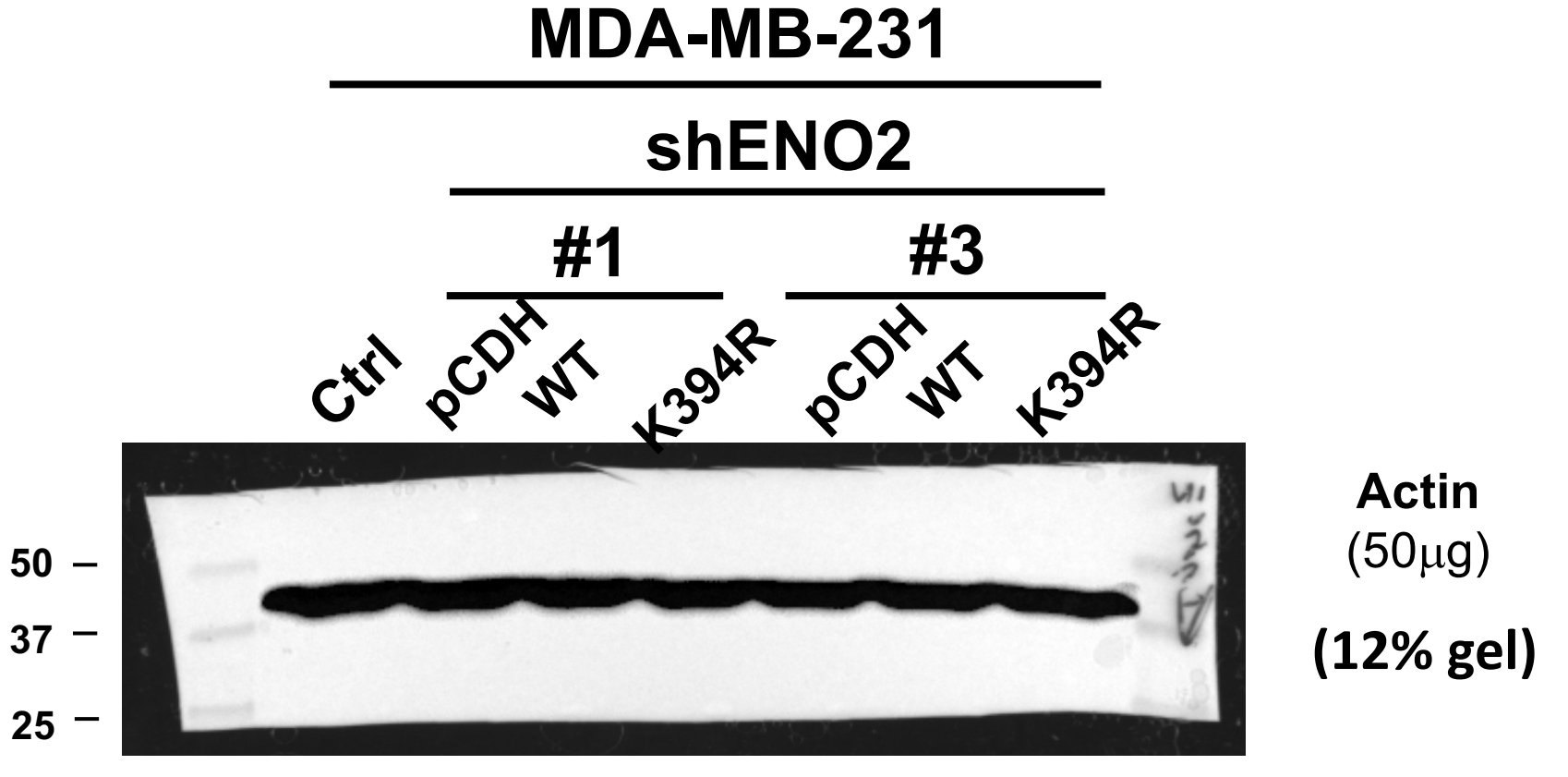

PAGE 5

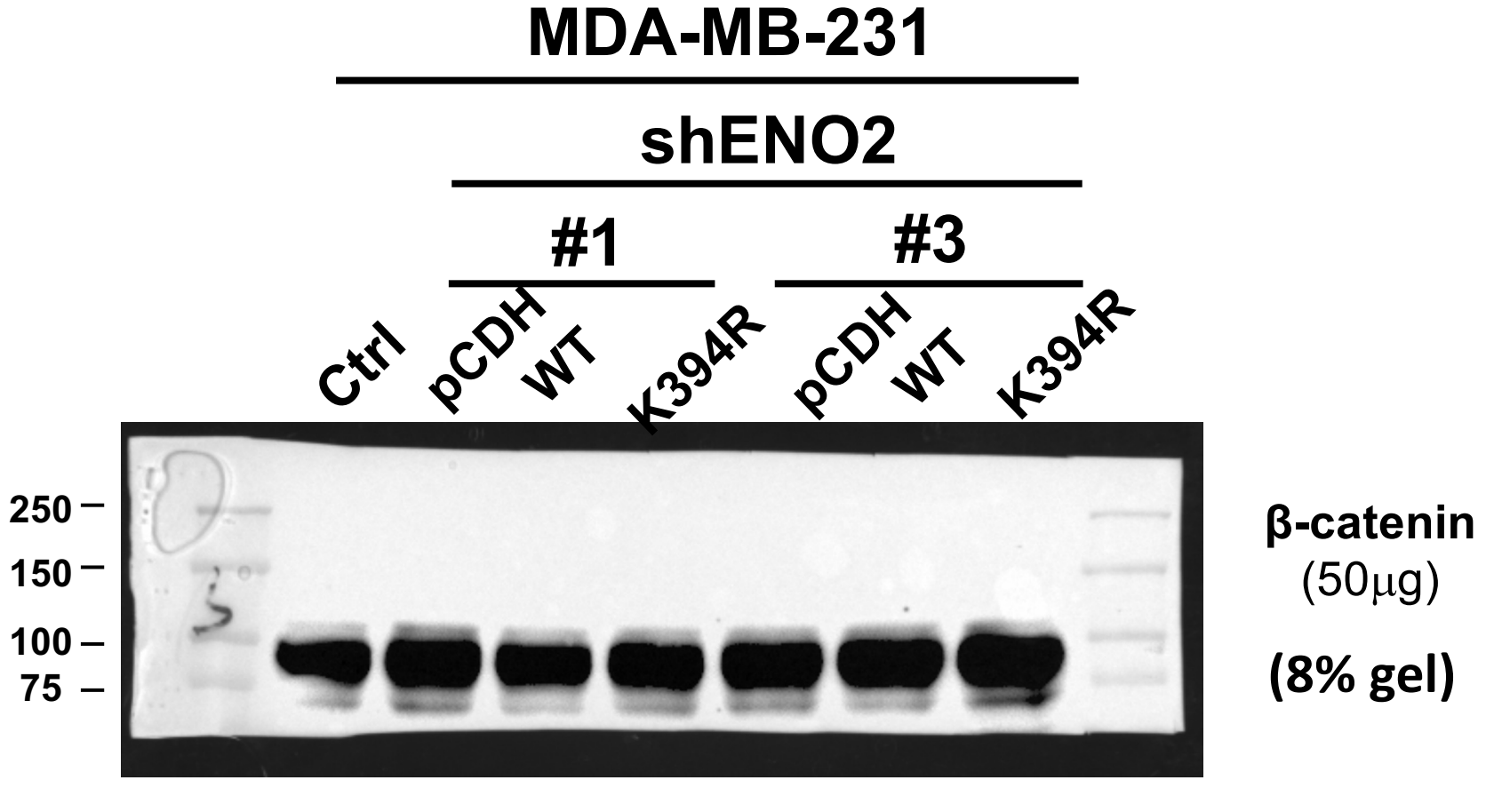

To produce data for Supplementary Figure S15B, 100 μg samples were loading into PAGE 1 and PAGE 2, 50 μg samples were loading into PAGE 3, 4 and 5 . PAGE 1, 2, 3, 4 were put into the same tank, PAGE 5 run with Supplementary Figure S15A independently. After protein transfer, membranes are sliced and hybridized with indicated antibodies.

Uncropped images

Supplementary Figure S16

PAGE 1

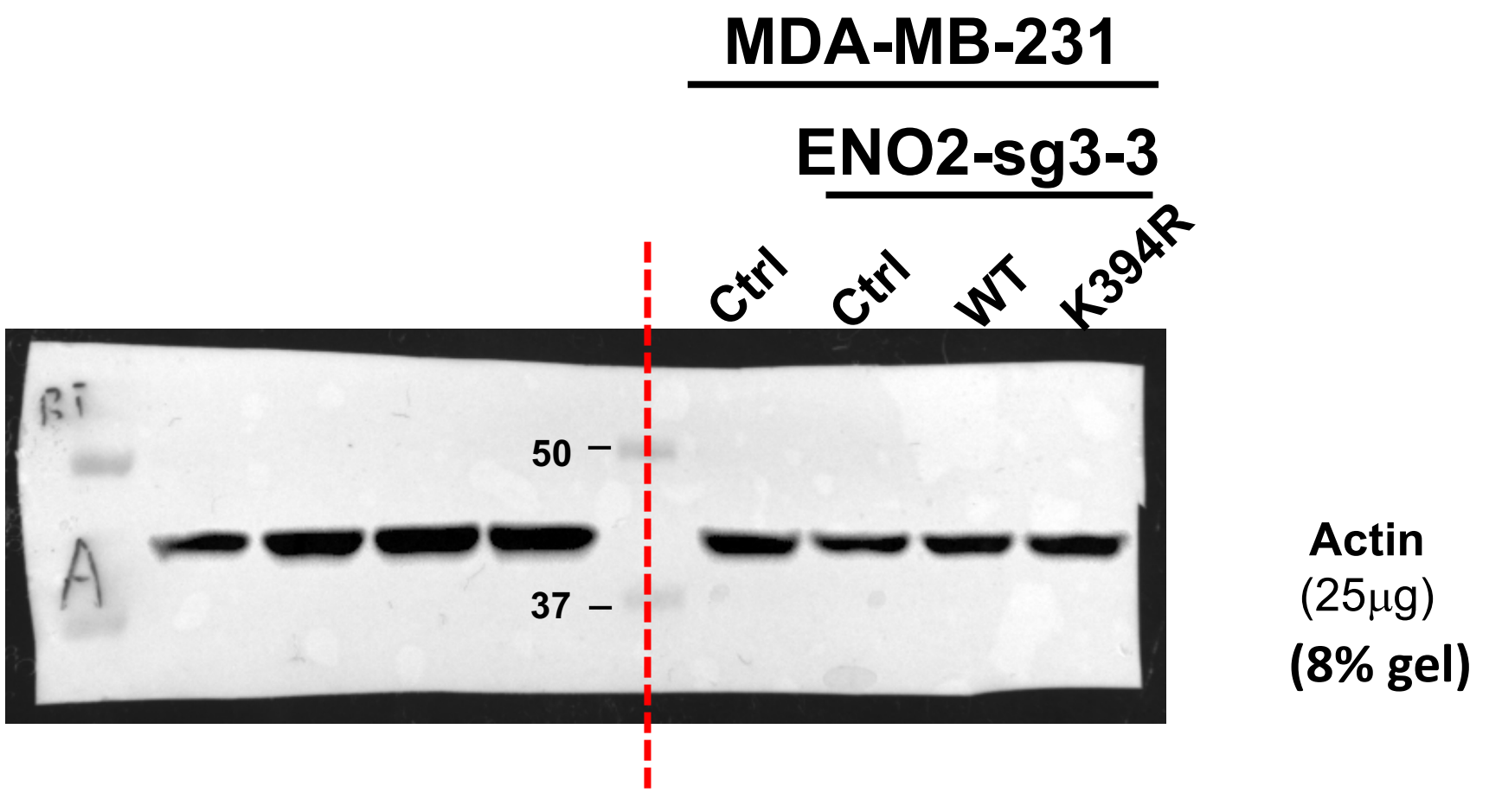

PAGE 2

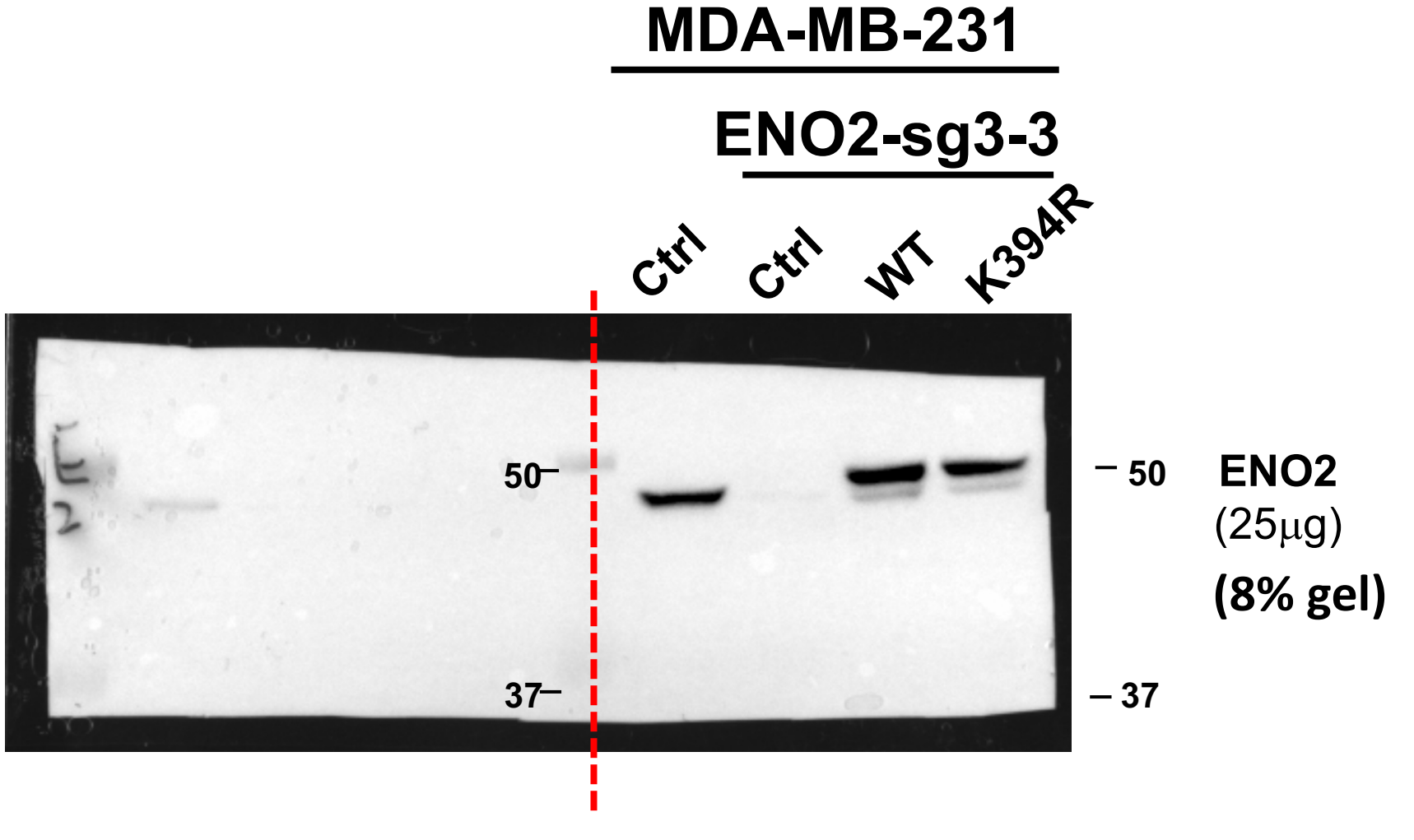

To produce data for Supplementary Figure S16, 25 μg samples were loading into PAGE 1 and PAGE 2. PAGE 1 and PAGE 2 were put into the same tank, and all the PAGEs were subjected to electrophoresis simultaneously. After protein transfer, membranes are sliced along the red dashed line and hybridized with indicated antibodies.
